# Highly Enantioselective Binaphthyl-Based Chiral Phosphoramidite Stabilized-Palladium Nanoparticles for Asymmetric Suzuki C–C Coupling Reactions

**DOI:** 10.1021/acs.inorgchem.3c00079

**Published:** 2023-03-06

**Authors:** Simay Ince, Özlem Öner, Mustafa Kemal Yılmaz, Mustafa Keleş, Bilgehan Güzel

**Affiliations:** † Institute of Science, Department of Nanotechnology and Advanced Materials, Mersin University, Mersin 33343, Turkey; ‡ Science Faculty, Department of Chemistry, Mersin University, Mersin 33343, Turkey; § Faculty of Arts and Sciences, Department of Chemistry, Osmaniye Korkut Ata University, Osmaniye 80000, Turkey; ∥ Faculty of Arts and Sciences, Department of Chemistry, Çukurova University, Adana 01330, Turkey

## Abstract

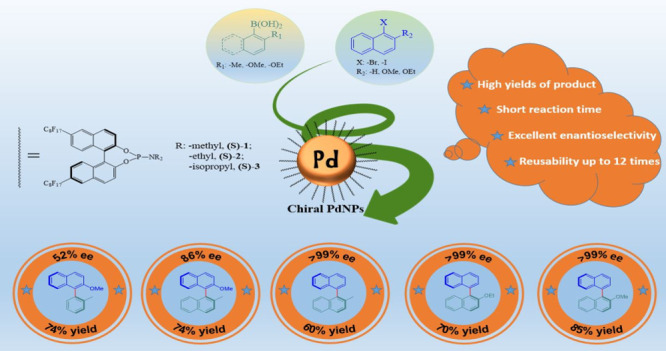

The optically pure binaphthyl-based phosphoramidite ligands and their perfluorinated analogs have been first used for the preparation of chiral palladium nanoparticles (PdNPs). These PdNPs have been extensively characterized by X-ray diffraction, X-ray photoelectron spectroscopy, transmission electron microscopy, ^31^P NMR, and thermogravimetric analysis techniques. The circular dichroism(CD) analysis of chiral PdNPs exhibited negative cotton effects. Perfluorinated phosphoramidite ligands provided smaller (2.32–3.45 nm) and well-defined nanoparticles, in comparison with the nonfluorinated analog (4.12 nm). The catalytic behavior of binaphthyl-based phosphoramidite stabilized chiral PdNPs has been investigated in the asymmetric Suzuki C–C coupling reactions for the formation of sterically hindered binaphthalene units, and high isolated yields (up to 85%) were achieved with excellent enantiomeric excesses (>99% ee). Recycling studies revealed that chiral PdNPs could be reused over 12 times without significant loss in activity and enantioselectivity (>99% ee). The nature of the active species was also investigated with a combination of poisoning and hot filtration tests and found that catalytically active species is the heterogeneous nanoparticles. These results indicate that the use of phosphoramidite ligands as a stabilizer for developing efficient and unique chiral nanoparticles could open up a field for many other asymmetric organic transformations promoted by chiral catalysts.

## Abstract 2

The catalytic behavior of binaphthyl-based phosphoramidite stabilized chiral PdNPs has been investigated in the asymmetric Suzuki C−C coupling reactions for the formation of sterically hindered binaphthalene units, and high isolated yields (up to 85#) were achieved with excellent enantiomeric excesses (>99# ee).

## 1 Introduction

Asymmetric Suzuki C–C coupling reaction is one of the most powerful and practical methods for the formation of axially chiral biaryl units which are an important class of atropisomeric compounds because of the need to use readily available and easy to handle both organoboron compounds and aryl halides as starting materials.^1−4^ Nonetheless, tremendous progress in this area has been focused on the use of well-defined soluble palladium complexes including popular and privileged chiral ligand systems such as bulky electron-rich phosphines and *N*-heterocyclic carbenes.^5,6^ In this field, despite the growing success of the homogenous catalysts in large-scale synthesis, lack of their reusability and recyclability seems to be major disadvantages in terms of economical and practical viewpoints.^1,7^ To overcome these challenges, homogenous catalytic systems have been changed with stabilized hybrid nanostructures sometimes called “semi-heterogeneous” or supported heterogeneous catalysts for many years. Especially, catalytic systems containing stabilized nanoparticles (i.e., with mainly organic ligands) offer many advantages such as unique size- and shape-dependent higher catalytic behavior and different electronic properties as well as their reusability and applicability after catalytic applications. For decades, thiols, ethylene glycol, polyols, amines, *N*-heterocyclic carbenes, and phosphine ligands have been widely used to fabricate stabilized metal nanoparticles.^6,8−12^ However, the aforementioned scenario includes only a few reports for the heterogeneous asymmetric transformations using chiral ligands. Furthermore, almost all of the chiral phosphine ligands such as binap, bipemp, segphos, etc., used to date in the asymmetric Suzuki C–C coupling reactions are commercial and binaphthyl-based phosphoramidite ligands, which have been known for their excellent enantioselectivity in a large number of homogenous asymmetric transformations,^7,13^ have not been reported to be a stabilizing agent on the palladium surface and a more efficient catalyst on any heterogeneous asymmetric coupling reaction. Similarly, although numerous chiral phosphine ligands have been both used as efficient stabilizers for palladium nanoparticles (PdNPs) and developed as catalysts for enantioselective organic reactions,^14−16^ Fujihara et al. reported the first and only example of the asymmetric Suzuki C–C coupling reaction catalyzed by chiral PdNPs stabilized with enantiomericially pure phosphine ligands ((*S*)-binap, (*S*)-tbinap, (*S*)-mop, (*S*)-etp, (*S*)-segphos, and (*S*)-diop), achieving up to 96% yield and moderate enantioselectivity (ee, <74%).^17^ The authors concluded that chiral phosphine stabilized-PdNPs have a small core (1.2–1.7 nm) with narrow size distribution, and they gave very different reactivities and enantioselectivities promoted by the metallic surface of the PdNPs. Since only one example of the asymmetric Suzuki C–C coupling reaction was reported using chiral phosphine stabilized-PdNPs, we aimed to use chiral binaphthyl-based phosphoramidites as a new stabilizing agent for this reaction. Surprisingly, the chiral ligand system that we chose served three functions of imparting chirality to the palladium surface, forming remarkably stable PdNPs with a very small core size and acting as a strong stabilizing agent to prevent agglomeration after numerous catalytic cycles.

## 2 Experimental Section

### 2.1 Materials and Instruments

Ligand synthesis and preparation of nanoparticles were carried out under an argon atmosphere using standard Schlenk techniques. Catalytic reactions were performed under air. Organic solvents and triethyl amine (Et_3_N) were purified and dried prior to use according to the standard procedures.^18^ Unless otherwise mentioned, all other solvents and reagents were used as received. (*S*)-6,6′-Diperfluorooctyl-1,1′-binaphthyl-2,2′-diol (perfluorinated BINOL)^19^ and dimethyl-(Me_2_NPCl_2_), diethyl-(Et_2_NPCl_2_), and diisopropylphosphoramidous dichloride (^*i*^Pr_2_NPCl_2_) were synthesized according to the literature.^20^ Flash column chromatography was carried out on conventional silica gel 60 (230–240 mesh, Merck). ^1^H, ^13^C, ^31^P, and ^19^F NMR spectra were recorded on a Bruker Avance 400 spectrometer and the chemical shifts (δ) were expressed in ppm relative to Me_4_Si as the internal standard at 400.2, 101.6, 162.0, and 376.5 MHz, respectively. Spin multiplicities were designated by the following symbols: s, singlet, bs, broad singlet, d, doublet; t, triplet; q, quartet; dd, doublet of doublets with coupling constants (*J*, Hz) or m, multiplet. The melting points (*M*_p_) of all synthesized phosphoramidite derivatives were determined by an MP90 digital melting point apparatus (Mettler Toledo) and uncorrected. The optical rotations were recorded on Rudolph Research Analytical (Autopol V Plus Automatic Polarimeter). A Waters SYNAPT G1 MS analyzer recorded high-resolution mass spectra (HRMS) of organic compounds. Chiral high-performance liquid chromatography (HPLC) analyses were performed on a Shimadzu LC-20AT Prominence Liquid Chromatograph comprising an LC-20AT VP pump with a Daicel Chiralcel OD-H or AD-H or OJ-H column (column temperature 25 °C). Racemic analogous of the coupling products described below was prepared under similar conditions in the presence of different palladium complexes or salts such as Pd(PPh_3_)_4_, Pd_2_(dba)_3_, or Pd(OAc)_2_. The enantiomeric excesses (ee) are an average of two runs with error margins ≤1%. X-ray diffraction (XRD) patterns of nanoparticles were recorded on a Panalytical Empyrean diffractometer with Cu-Kα radiation (40 kV, 15 mA, 1.54051 A) over a 2θ range from 20 to 90° at room temperature. Thermogravimetric analyses were performed on a TGA 3+ model Mettler Toledo thermogravimetric balance. Circular dichroism (CD) spectra were recorded on a Jasco J-815 spectrophotometer. The spectra were measured with a scan speed of 100 nm min^–1^ and the spectral range of 250–400 nm. XPS analyses were measured on a Specs-Flex marka FlexPS spectrometer with a monochromatic Al X-ray source.

### 2.2 Synthesis of (*S*)-6,6′-Diperfluorooctyl-1,1′-binaphthyl-2,2’-diyl-*N,N*-dimethylphosphorus Amidite ((*S*)-1)

To a solution of perfluorinated (*S*)-BINOL (1.0 g, 0.88 mmol) and trimethylamine (495 μL, 3.52 mmol) in dry THF (20 mL) was added dimethylphosphoramidous dichloride (208 μL, 1.76 mmol), and the suspension was stirred under argon atmosphere at room temperature for half an hour. After this time, the resulting precipitate was filtered off. The solvent was evaporated, and the residue was purified by flash chromatography on a plug of silica as quickly as possible, using degassed *n-*hexane as the eluent, to give (*S*)-1 (0.967 g, 92%). White solid; *M*_p_: 70–71 °C; *R*_f_: 0.7 (hexane/ethyl acetate, 5:1); [α]_*D*_^20^ = −23.1 (*c* = 0.11, THF); ^1^H NMR (400.2 MHz, CDCl_3_): δ (ppm) 8.12 (d, *J* = 5.6 Hz, 2H, ArH), 8.01 (d, *J* = 8.8 Hz, 1H, ArH), 7.94 (d, *J* = 8.8 Hz 1H, ArH), 7.53 (d, *J* = 8.8 Hz, 1H, ArH), 7.42 (d, *J* = 8.8 Hz, 1H, ArH), 7.35 (s, 2H, ArH), 7.31 (s, 2H, ArH), 2.48 (d, *J*_PH_ = 9.1 Hz, 6H, N–CH_3_); ^13^C NMR (101.6 MHz, CDCl_3_): δ (ppm) 151.2 (d, *J*_PC_ = 5.1 Hz, C–O), 150.6 (s), 133.0 (d, *J* = 19.7 Hz), 130.4 (d, *J* = 31.0 Hz), 129.2 (s), 128.60 (s), 127.3 (dd, *J* = 14.1, 6.4 Hz), 126.2 (d, *J* = 14.9 Hz), 124.3 (dd, *J* = 45.8, 23.9 Hz), 122.5 (d, *J* = 7.3 Hz), 122.1 (d, *J* = 5.2 Hz), 121.3 (d, *J* = 1.8 Hz), 117.6 (t, *J* = 32.5 Hz), 114.7 (t, *J* = 34.3 Hz), 34.9 (d, *J*_PC_ = 21.2 Hz, CH_3_); ^19^F NMR (376.5 MHz, CDCl_3_): δ (ppm) -80.93 (t, *J*_FF_ = 9.9 Hz, 6F, CF_3_), −110.09 (dd, *J*_FF_ = 24.4, 12.3 Hz, 3F, α-CF_2_ (2F) ve α*’*-CF_2_ (1F)), −110.33 (t, *J*_FF_ = 14.3 Hz, 1F, α*’*-CF_2_), −121.25 (bs, 4F, CF_2_), −121.43 (bs, 2F, CF_2_), −121.57 (bs, 2F, CF_2_), −121.94 (bs, 8F, CF_2_), −122.78 (bs, 4F, CF_2_), −126.20 (bs, 4F, CF_2_); ^31^P NMR (162.0 MHz, CDCl_3_): δ (ppm) 150.10 (s); HRMS (TOF-MS-ESI) calculated for C_38_H_16_F_34_NO_2_P ([M + H]^+^): 1196.0454; found: 1196.0616.

### 2.3 Synthesis of (*S*)-6,6′-Diperfluorooctyl-1,1′-binaphthyl-2,2’-diyl-*N,N*-diethylphosphorus Amidite ((*S*)-2)

The same preceding procedure was applied using perfluorinated (*S*)-BINOL (1.0 g, 0.88 mmol) and diethylphosphoramidous dichloride (264 μL, 1.76 mmol) to give compound (*S*)-2 (0.99 g, 92%). White solid; *M*_p_: 112–113 °C; *R*_f_: 0.75 (hexane/ethyl acetate, 5:1); [α]_*D*_^20^ = −20.6 (*c* = 0.15, THF); ^1^H NMR (400.2 MHz, CDCl_3_): δ (ppm) 8.12 (d, *J* = 2.1 Hz, 2H, ArH), 8.02 (d, *J* = 8.8 Hz, 1H, ArH), 7.95 (d, *J* = 8.8 Hz 1H, ArH), 7.54 (d, *J* = 8.8 Hz, 1H, ArH), 7.41 (d, *J* = 8.8 Hz, 1H, ArH), 7.36 (s, 2H, ArH), 7.31 (s, 2H, ArH), 3.03–2.90 (m, 2H, N–CH_2_), 2.85–2.72 (m, 2H, N–CH_2_), 0.99 (t, *J*_PH_ = 7.0 Hz, 6H, CH_3_); ^13^C NMR (101.6 MHz, CDCl_3_): δ (ppm) 152.3 (d, *J*_PC_ = 5.3 Hz, C–O), 151.7 (s), 134.03 (d, *J* = 17.8 Hz), 131.3 (d, *J* = 41.3 Hz), 130.2 (s), 129.5 (s), 128.3 (dd, *J* = 11.9, 6.7 Hz), 127.3 (d, *J* = 13.9 Hz), 125.6 (s), 125.4–125.2 (m), 125.1 (d, *J* = 3.7 Hz), 124.8 (s), 123.7 (s), 123.6 (d, *J* = 5.1 Hz), 123.5 (s), 123.0 (t, *J* = 3.9 Hz), 122.0 (d, *J* = 1.9 Hz), 118.6 (dd, *J* = 4.0, 2.6 Hz), 116.0 (t, *J* = 3.5 Hz), 38.4 (d, *J*_PC_ = 21.7 Hz, CH_2_), 14.6 (d, *J*_PC_ = 2.4 Hz, CH_3_); ^19^F NMR (376.5 MHz, CDCl_3_): δ (ppm) -80.82 (t, *J*_FF_ = 9.9 Hz, 6F, CF_3_), −110.09 (dd, *J*_FF_ = 24.4, 12.3 Hz, 3F, α-CF_2_ (2F) ve α*’*-CF_2_ (1F)), −110.27 (t, *J*_FF_ = 14.2 Hz, 1F, α*’*-CF_2_), −121.21 (bs, 4F, CF_2_), −121.37 (bs, 2F, CF_2_), −121.56 (bs, 2F, CF_2_), −121.88 (bs, 8F, CF_2_), −122.72 (bs, 4F, CF_2_), −126.13 (bs, 4F, CF_2_); ^31^P NMR (162.0 MHz, CDCl_3_): δ (ppm) 151.00 (s); HRMS (TOF-MS-ESI) calculated for C_40_H_20_F_34_NO_2_P ([M + H]^+^): 1224.0767; found: 1224.0870.

### 2.4 Synthesis of (*S*)-6,6′-Diperfluorooctyl-1,1′-binaphthyl-2,2’-diyl-*N,N*-diisopropylphosphorus Amidite ((*S*)-3)

The same preceding procedure was applied using perfluorinated (*S*)-BINOL (1.0 g, 0.88 mmol) and diisopropylphosphoramidous dichloride (341 μL, 1.76 mmol) to give compound (*S*)-3 (0.99 g, 90%). White solid; *M*_p_: 64–65 °C; *R*_f_: 0.77 (hexane/ethyl acetate, 5:1); [α]_*D*_^20^***= –***24.5 (*c* = 0.11, THF); ^1^H NMR (400.2 MHz, CDCl_3_): δ (ppm) 8.11 (s, 2H, ArH), 8.01 (d, *J* = 8.8 Hz, 1H, ArH), 7.95 (d, *J* = 8.8 Hz 1H, ArH), 7.54 (d, *J* = 8.8 Hz, 1H, ArH), 7.47 (d, *J* = 8.8 Hz, 1H, ArH), 7.36–7.23 (m, 2H, ArH), 3.31 (qd, *J*_PH_ = 13.5, 6.8 Hz, 2H, N–CH), 1.14 (dd, *J*_PH_ = 14.67, 6.7 Hz, 12H, CH_3_); ^13^C NMR (101.6 MHz, CDCl_3_): δ (ppm) 152.6 (d, *J*_PC_ = 7.2 Hz, C–O), 152.3 (s), 134.0 (d, *J* = 7.1 Hz), 131.5 (s), 130.6 (s), 130.2 (s), 129.3 (s), 128.2 (d, *J* = 6.5 Hz), 127.4 (d, *J* = 3.5 Hz), 125.3 (s), 124.9 (s), 123.9 (s), 123.6 (d, *J* = 5.1 Hz), 122.9 (dd, *J* = 10.6, 5.7 Hz), 121.3 (d, *J* = 1.0 Hz), 118.6 (dd, *J* = 4.2, 2.5 Hz), 116.0 (s), 115.7 (d, *J* = 2.6 Hz), 45.0 (d, *J*_PC_ = 12.8 Hz, CH), 24.4 (d, *J*_PC_ = 8.5 Hz, CH_3_); ^19^F NMR (376.5 MHz, CDCl_3_): δ (ppm) −80.82 (t, *J*_FF_ = 9.9 Hz, 6F, CF_3_), −110.09 (dd, *J*_FF_ = 24.4, 12.3 Hz, 3F, α-CF_2_ (2F) ve α*’*-CF_2_ (1F)), −110.27 (t, *J*_FF_ = 14.2 Hz, 1F, α*’*-CF_2_), −121.21 (bs, 4F, CF_2_), −121.37 (bs, 2F, CF_2_), −121.56 (bs, 2F, CF_2_), −121.88 (bs, 8F, CF_2_), −122.72 (bs, 4F, CF_2_), −126.13 (bs, 4F, CF_2_); ^31^P NMR (162.0 MHz, CDCl_3_): δ (ppm) 153.88 (s); HRMS (TOF-MS-ESI) calculated for C_42_H_24_F_34_NO_2_P ([M + H]^+^): 1252.1080; found: 1252.1085.

### 2.5 General Preparation of Phosphoramidite Stabilized-Palladium Nanoparticles

The tetrahydrofuran (10 mL) solution of chiral phosphoramidite ligands (0.298 g, 0.25 mmol) was introduced in a Schlenk tube and stirred vigorously under a nitrogen atmosphere. The methanol solution of K_2_PdCl_4_ (40.5 mg, 0.125 mmol) was added to the stirring solution of ligands at room temperature. After about an hour of stirring, the resulting complex solution was cooled in an ice bath and reduced by the addition of NaBH_4_ (37.8 mg, 0.625 mmol). A color change from light yellow to black was observed immediately, indicating nanoparticle formation. After an additional 2 h of stirring, the black powder was isolated by centrifugation (6000 rpm, 15 min), washing with deionized water, THF, and ethanol, respectively, and then dried under reduced pressure.

### 2.6 General Procedure for the Asymmetric Suzuki C–C Coupling Reaction and Recycling Studies

A Schlenk tube containing solid materials, i.e., boronic acid (1.5 mmol), base (3.0 mmol), and phosphoramidite stabilized-PdNP was purged under the argon atmosphere without using any solvent for 20 min. A solution of aryl halide (1.0 mmol) in solvent (4.0 mL) was injected, and the mixture was stirred at desired temperature (in a previously heated oil bath) within a specified period. Then, water was added to the reaction mixture and extracted with chloroform. The layers were separated, and the aqueous layer was further extracted with chloroform. The combined organic extracts were dried over Na_2_SO_4_, filtered, and the solution was concentrated and purified by flash column chromatography on silica gel to afford the coupling products. The reaction yield and enantiomeric excess were determined by HPLC analysis. The recycle experiment was carried out using the catalyst (*S*)-2@PdNP or NF(*S*)-2@PdNP (3.0 mg) under the optimal conditions. At the end of each reaction, the catalyst was separated from the reaction mixture by simple centrifugation, washed thoroughly with water followed by ethanol, dried under vacuum and reused.

## 3 Results and Discussion

### 3.1 Ligand Design as a Stabilizer and Characterization

**Scheme 1:**
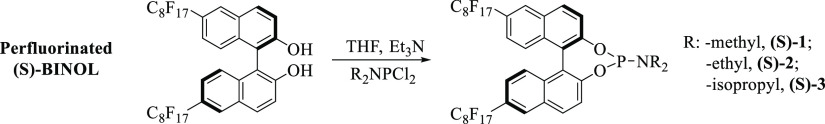
Synthesis of Binaphthyl-Based Chiral Phosphoramidite Ligands as a Protecting Agent

The importance of binaphthyl-based chiral phosphoramidite ligands to the continuing development of enantioselective reactions has been the major driving force behind our group’s interest in the investigation as both a novel stabilizer and an efficient approach to create an optically active chiral palladium surface. Moreover, their strong donor properties, good stability, and especially extensive interaction surfaces should be make them more attractive for protection to the metal. On the other hand, easily accessible perfluorinated amines or thiols or some perfluoro-tagged compounds have been widely employed a stabilizing agent as they can prevent agglomeration and coalescence by forming a protective surrounding shell around the metallic particles due to their strong van der Waals interactions.^21−25^ We combined these two perspectives in order to provide additional protection to the palladium nanoparticle and designed various binaphthyl-based phosphoramidite ligands bearing perfluorooctyl groups at 6,6′- positions of the binaphthyl unit (Scheme 1). Also, phosphoramidite ligands were substituted with -methyl, -ethyl, and -isopropyl groups based on secondary amine, and the effect of these substituents upon the yield and enantioselectivity in the asymmetric Suzuki C–C coupling reaction were tested. We would like to strongly emphasize that these triple combinations resulted in the formation of nearly monodispersed palladium nanoparticles in the range of 2.32–3.45 nm and revealed a catalyst that is both superb enantioselective and can be used repeatedly for the asymmetric Suzuki C–C coupling reactions.

Perfluorinated (*S*)-BINOL was prepared using (*S*)-enantiomer of 1,1′-bi-2-naphthol according to previously method of Sinou and co-workers.^19^ Enantiopure phosphoramidite ligands ((*S*)-1-3) were obtained subsequent reaction of perfluorinated (*S*)-BINOL with dialkyl phosphoramidous dichlorides in the presence of Et_3_N.^26^ It should be emphasized that no racemization was observed during the ligand synthesis when comparing the HPLC chromatograms of each enantiomer (Figures S16–S19). The structural characterization of phosphoramidite ligands has been determined by NMR (nuclear magnetic resonance) spectroscopy and HRMS (high-resolution mass spectroscopy) analyses, and in addition, optical rotations were also identified by polarimetric measurements. The ^31^P NMR spectrum showed resonance signals at δ 150.1 ppm for (*S*)-1, δ 151.0 ppm for (*S*)-2, and δ 153.8 ppm for (*S*)-3, and these are significantly shifted to lower frequencies compared to the respective dialkyl phosphoramidous dichlorides, δ 165.9 ppm for Me_2_NPCl_2_,^27^ δ 162.9 ppm for Et_2_NPCl_2_,^20^ and δ 169.4 ppm for ^*i*^Pr_2_NPCl_2_.^28^ In the ^1^H NMR spectrum of the ligands, the doublet or doublet of doublet signals for the methyl protons were found at δ 2.48 ppm (d: *J*_PH_ = 9.1 Hz, for (*S*)-1), δ 0.99 ppm (d: *J*_PH_ = 7.0 Hz, for (*S*)-3), and δ 1.14 ppm (dd: *J*_PH_ = 14.6 and 6.7 Hz, for (*S*)-3) due to the phosphorus-hydrogen coupling, respectively. Meanwhile, signals for CH_2_ and CH protons in (*S*)-2 and (*S*)-3 appeared at δ 3.03–2.90 (m, 2H, N–CH_2_), 2.85–2.72 (m, 2H, N–CH_2_), and 3.31 (qd, *J*_PH_ = 13.5, 6.8 Hz, 2H, N–CH) ppm, and the aromatic protons exhibited in the range of δ 8.12–7.23 ppm. ^19^F and ^13^C NMR chemical shifts and HRMS results were also consistent with the proposed structures of the phosphoramidite ligands.^29−31^

### 3.2 Synthesis and Characterization of Binaphthyl-Based Phosphoramidite Stabilized-PdNPs

The use of phosphine ligands as a protecting agent for the formation of PdNPs has been reported by several authors as they have a strong interaction between phosphorus atoms and metal surfaces (Table 1). However, there are only a few reports on the catalytic applications of these PdNPs. For example, Hyeon and coworkers reported trioctylphosphine (top) as a surfactant to stabilize PdNPs and then used this monodisperse nanoparticles (3.5–7.0 nm) in order to produce different phosphine stabilized nanoparticles (5.0 nm) including chiral and water-dispersible ones via ligand exchange reactions. They also investigated the coordination chemistry of the phosphine ligands on the nanoparticle surface by using ^31^P NMR spectroscopy.^32,33^ Tamura and Fujihara reported the first synthesis of optically active (*R*)- and (*S*)-binap-protected PdNPs (2.0 nm), and they used these chiral catalysts in asymmetric hydrosilylation of styrene and subsequent oxidation of the C–Si bond, which is an important reaction for the preparation of chiral secondary alcohols.^14^ Chaudret et al. reported the preparation of PdNP using the chiral xylofuranoside diphosphite ligand as a stabilizer. This ligand was chosen because it has phosphorus and oxygen atoms that can be tightly and weakly coordinated on the metal surface, respectively. This chiral PdNP was found to be active in the asymmetric allylic alkylation of *rac*-3-acetoxy-1,3-diphenyl-1-propene with dimethyl malonate, and no significant decomposition of the catalyst was observed even after 168 h of the reaction.^16^ In 2006, Fujihara and co-workers reported the use of 6-octyl-substituted bisphosphine (C8-binap) stabilized-PdNPs, which exhibited good activities for the Stille and Suzuki coupling reactions. The alkyl chain on the binaphthyl unit of this ligand provided additional stability with a small core size and narrow size distribution (1.2 ± 0.2 nm) for the PdNPs and could be recycled two times without loss of activity for the Stille reaction of 2-iodobenzoate with 2-(tributylstannyl)thiophene.^15^ The use of commercially available optically active mono- and bisphosphines as a protecting agent to stabilize the palladium surface was also reported by the same group in 2008. Using these chiral PdNP systems (1.2–1.7 nm), moderate to good conversions and enantioselectivities were achieved in the asymmetric Suzuki coupling reactions.^17^ Although there are a number of studies on organic transformations catalyzed by phosphine stabilized-PdNPs, the use of chiral phosphine ligands in heterogeneous asymmetric catalysis is still limited. For this purpose, we are interested in phosphoramidite ligands used for the preparation of heterogeneous chiral PdNPs for the first time in the literature, and to expand the scope of chiral phosphine ligands in this field, we aimed to evaluate the reactivity, enantioselectivity, and reusability of phosphoramidite stabilized-PdNPs in the asymmetric Suzuki C–C coupling reaction.

**Table 1:** Nonsupported Phosphine Stabilized-Palladium Nanoparticles and Catalytic Applications^a^

stabilizer	mean size of PdNPs	catalytic application	catalyst loading	temp. and reac. time	yield (%) and ee (%)	ref.
top	3.5–7.0 nm					(32)
(R) or (*S*)-binap	2.0 ± 0.5 nm	asymmetric hydrosilylation		25 °C (5 h)	after single run >99% ee and 81% yield	(14)
xylofuranoside diphosphite	4.25 ± 0.04 nm	asymmetric allylic alkylation	1.0 mol % + excess ligand	25 °C (24–168 h)	after single run 94–96% ee and 56–61% yield	(16)
tfp, tpp, tcp, dppe, dppb, bdpf, biphep, (-)-diop, (-)-binap, (*R*,*R*)-norphos, tppds, bdsppb	5 nm					(33)
rac-C_8_-binap	1.2 ± 0.2 nm	stille and Suzuki coupling	0.06 and 0.02 mol %	25 °C (5 h) and 25 °C (24 h)	1st run: 91% yield, 2nd run: 90% yield and after single run 83% yield	(15)
(*S*)-binap, (*S*)-tbinap, (*S*)-segphos, (4*S*,5*S*)-diop, (*S*)-mop, (*S*)-etp	1.2–1.7 nm	asymmetric Suzuki coupling	0.1 mol %	25 °C (3–72 h)	after single run 10–74% ee and 25–96% yield	(17)
binaphthyl-based phosphoramidite ligands	2.32–3.45 nm	asymmetric Suzuki coupling	1.0 mol %	80 °C (1 h)	reused up to 12 cycles (up to 85% yield and >99% ee)	this work

a binap, 2,2′-bis(diphenylphosphino)-1,1′-binaphthyl; C_8_-binap, 6-Me(CH_2_)_7_SCH_2_–2,2′-bis(diphenylphosphino)-1,1′-binaphthyl; top, trioctylphosphine; tfp, trifurylphosphine; tpp, triphenylphosphine; tcp, tricyclohexylphosphine; dppe, 1,2-bis(diphenylphosphino)ethane; dppb, 1,2-bis(diphenylphosphino)butane; bdpf, 1,1′-ferrocenediyl-bis(diphenylphosphine); biphep, 2,2′-bis(diphenylphosphino)-1,1′-biphenyl; (-)-diop, (-)-2,3-O-isopropylidene-2,3-dihydroxy-1,4-bis(diphenylphosphino)butane; (*R*,*R*)-norphos, 2,3-bis(diphen-ylphosphanyl) bicycle[2.2.1]hept-5-ene; tppds, 3,3′-phenylphosphinediylbenzenesulfonic acid disodium salt; bdsppb, 1,2-bis(di-4-sulfonatophenylphosphino)benzene tetrasodium salt; (*S*)-tbinap, (*S*)-2,2′-bis(di-p-tolylphosphino)-1,1′-binaphthalene; (*S*)-segphos, (4,4′-bi-1,3-benzodioxole)-5,5′-diylbis(diphenylphosphine); (*S*)-mop, (*S*)-2-(diphenylphosphino)-2′-methoxy-1,1′-binaphthyl; (*S*)-etp, (*S*)-2-(diphenylphosphino)-2′-ethyl-1,1′-binaphthalene.

Herein, we synthesized chiral PdNPs stabilized by perfluorinated binaphthyl-based phosphoramidites as new capping ligands. To answering and carrying out some comparative studies on the role of the perfluorinated chain attached to the binaphthyl unit about the size distribution, controllability of the particle size, catalytic efficiency, and reusability of the NPs, we also prepared the nonfluorinated analog of ethyl-substituted phosphoramidite (NF(*S*)-2) stabilized-palladium nanoparticles (NF(*S*)-2@PdNP). The (*S*)-1-3@PdNPs obtained were highly dispersible in the perfluorinated solvent such as perflorohexane (FC-72) but slightly dispersible in common polar organic solvents (MeOH, EtOH, CHCl_3_, THF etc.), thus simplifying the purification step by repeated precipitation in EtOH. Notably, (*S*)-1-3@PdNPs and NF(*S*)-2@PdNPs were stable for several months.

**Figure 1:**
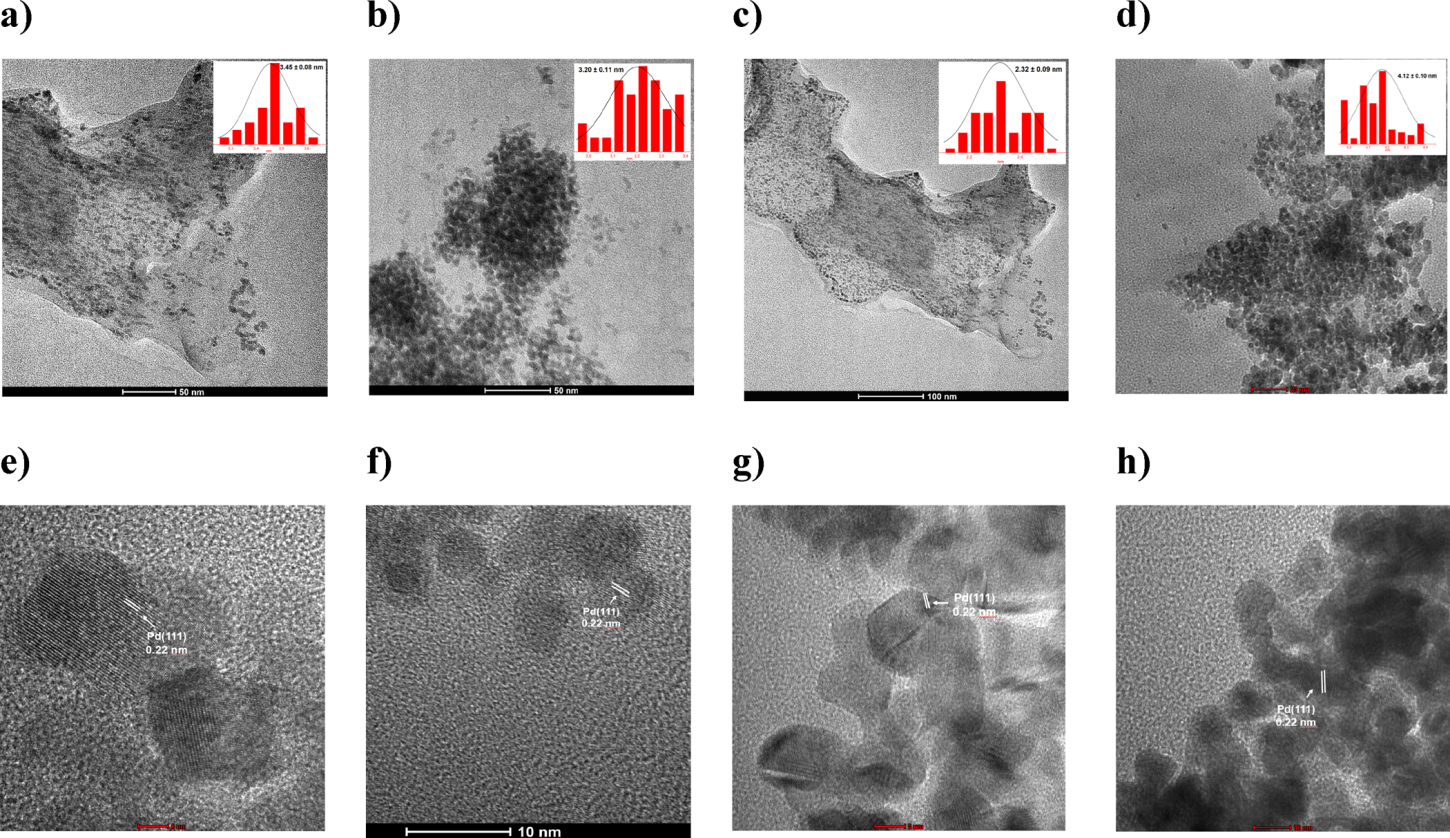
TEM images of the PdNPs stabilized with (*S*)-1 (a), (*S*)-2 (b), (*S*)-3 (c), and NF(*S*)-2 (d). TEM images show an average particle size of 3.45 ± 0.08 nm for (*S*)-1@PdNP, 3.20 ± 0.11 nm for (*S*)-2@PdNP, 2.32 ± 0.09 nm for (*S*)-3@PdNP, and 4.12 ± 0.10 nm for NF(*S*)-2@PdNP. Lattice distance values obtained from TEM images for (*S*)-1@PdNP (e), for (*S*)-2@PdNP (f), for (*S*)-3@PdNP (g), and for NF(*S*)-2@PdNP (h).

Transmission electron microscopy (TEM) images of all the (*S*)-1-3@PdNPs and NF-(*S*)-2@PdNPs formed from NaBH_4_ reduction are shown in Figure 1. The PdNPs showed spherical and a narrow size distribution with a mean size of 3.45 (±0.08) nm for (*S*)-1@PdNP, 3.20 (±0.11) nm for (*S*)-2@PdNP, 2.32 (±0.09) nm for (*S*)-3@PdNP, and 4.12 ± 0.10 nm NF-(*S*)-2@PdNP with no evidence of aggregation. TEM images showed PdNP formation for (*S*)-1-3@PdNPS in the size range of 3.45–2.32 nm depending on the phosphoramidite used so that the effect of ligands on the size of nanoparticles could also be clearly seen. More precisely, the presence of the *isopropyl* group attached to the nitrogen atom (for ligand (*S*)-3) enhances the σ-donor ability of the P atom, and the strong interaction it creates allows the yield of well-controlled small nanoparticles. Furthermore, the TEM images exhibited clear lattice fringes with a *d*-spacing of 0.22 nm, which corresponded to the Pd(111) lattice planes of face-centered cubic (fcc) crystal structure Pd.^34^ These results supported the presence of long perfluorinated chains, which induces steric stabilization, prevents agglomeration of nanoparticles, and gives them a special shape, so that PdNPs produced with nonfluorinated analogs could be obtained in larger sizes. The particle size of all nanoparticles obtained from TEM was in good agreement with the average crystallite size determined from the XRD.

**Figure 2:**
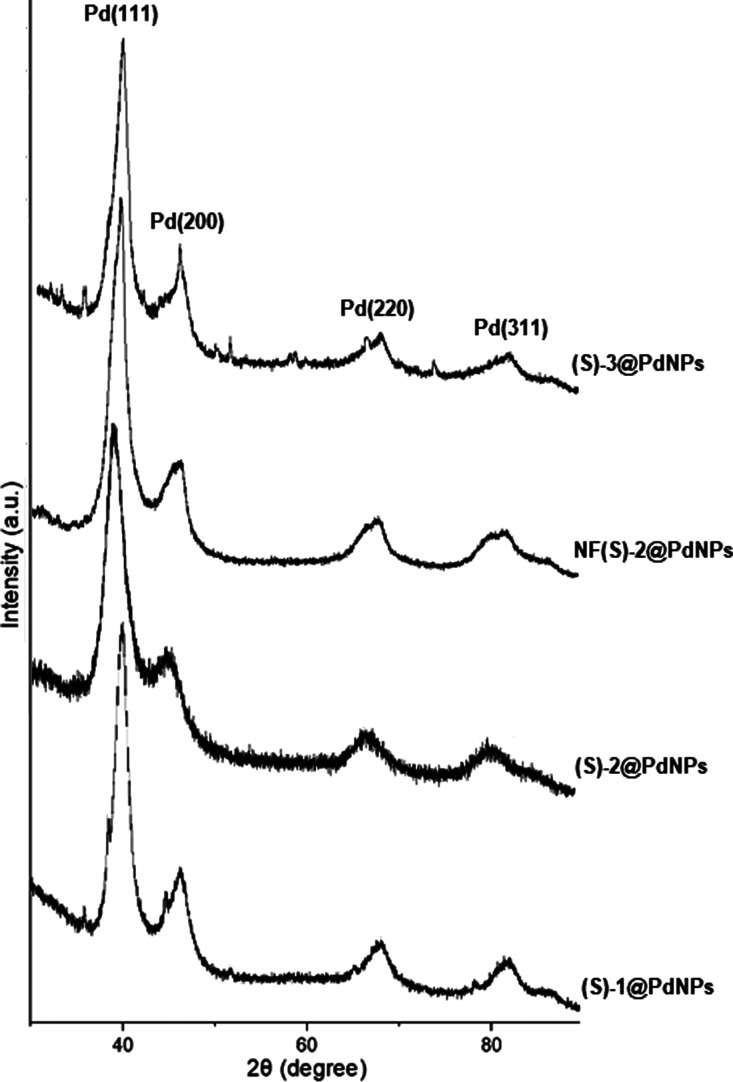
XRD patterns for (*S*)-1-3@PdNPs and NF(*S*)-2@PdNP.

The XRD patterns of phosphoramidite stabilized-palladium nanoparticles ((*S*)-1-3@PdNP and NF(*S*)-2@PdNP) showed broad peaks at around 2θ = 40°, which can be indexed to the characteristic reflection {111} plane for the face-centered-cubic (fcc) structure of zerovalent Pd (Figure 2). The diffraction peaks of palladium nanoparticles positioned at 2θ = 46.0, 68.0, and 81.0 that correspond to {200}, {220}, and {311} Bragg planes of Pd, can also be observed.^35−37^ All XRD peaks can be well indexed to a fcc lattice system according to the COD database (COD ID: 1011112).^38^ The crystallite size of (*S*)-1-3@PdNPs calculated from the line broadening of (111) reflection using the Scherer’s equation showed an average size of ∼3–4 nm, while ∼6 nm of average particle size was obtained for the NF(*S*)-2@PdNPs.

**Figure 3:**
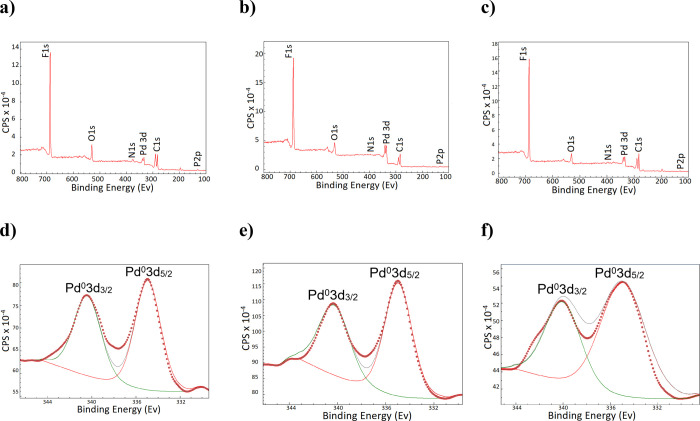
XPS spectra of (*S*)-1@PdNP (a), (*S*)-2@PdNP (b), and (*S*)-3@PdNP (c). High-resolution scans of Pd 3d XPS core-level spectra for (*S*)-1@PdNP (d), (*S*)-2@PdNP (e), and (*S*)-3@PdNP (f).

The XPS (X-ray photoelectron spectroscopy) spectra of (*S*)-1-3@PdNPs displayed the binding energies for the Pd(0) 3d doublets are 334.9 and 340.3 eV for (*S*)-1@PdNP, 334.9 and 340.2 eV for (*S*)-2@PdNP, and 335.0 and 340.1 eV for (*S*)-3@PdNP, respectively (Figure 3). These results indicate that palladium exist as metallic in all (*S*)-1-3@PdNPs rather than the oxide form due to the perfluorinated phosphoramidite wrapping of the Pd(0) surface during the nanoparticle preparation.^39^ However, binding energy peaks at 342.3 eV (Pd 3d3) and 337.8 eV (Pd 3d5), which are barely visible in the XPS spectrum for (*S*)-3@PdNPs, are attributed to Pd(II).^40^ Compared to (*S*)-1-2@PdNPs, this oxidation state for (*S*)-3@PdNP was related to the PdNP sizes; a decrease of their size resulted in a higher reactivity toward oxygen present in air.^41,42^ XPS analysis can also provide insights into the chemical identification of fluorine, phosphorus, nitrogen, and oxygen atoms on the nanoparticle surface. In this context, corresponding XPS spectra exhibited the strong characteristic signal of F 1s (687.0 eV for (*S*)-1@PdNP, 688.2 eV for (*S*)-2@PdNP, and 687.0 eV for (*S*)-3@PdNP) as well as weak N 1s (398.1 eV for (*S*)-1@PdNP, 398.0 eV for (*S*)-2@PdNP, and 399.1 eV for (*S*)-3@PdNP), O 1s (529.5 eV for (*S*)-1@PdNP, 531.7 eV for (*S*)-2@PdNP, and 531.7 eV for (*S*)-3@PdNP), and P 2p (132.4 eV for (*S*)-1@PdNP, 132.5 eV for (*S*)-2@PdNP, and 131.9 eV for (*S*)-3@PdNP), as expected.^35^ These findings suggest that all expected elements (F, P, N, and O) are preferentially located at the nanoparticle surface.

The presence of the phosphoramidites on the palladium nanoparticles was further investigated by solution-state ^31^P NMR analysis, which would both prove the presence of the ligand on the nanoparticle surface and provide useful information about the ligand’s coordination. However, not surprisingly, the phosphorus signals were not observed for the solution-state ^31^P NMR spectra of the nanoparticles recorded in THF-d.^8^ This expected result especially for the phosphine stabilized nanoparticles is explained in the literature as follows: ligands used as a stabilizer can become almost immobile as a result of their strong coordination with the nanoparticle surface and therefore cannot give any signals under conventional solution-state NMR conditions. In such a case, three different ways are generally used to prove the presence of the ligand on the nanoparticle surface. In the first, excess ligand is progressively added into the NMR solution containing the nanoparticles, and a rapid ligand exchange process occurs between free and attached ligands. In the second, an oxidant such as H_2_O_2_ is added to the NMR mixture of nanoparticles, and the ligand is released from the nanoparticle surface after oxidation, and in the third, the solid-state NMR techniques can be applied.^43−45^ Considering these three methods, we aimed to shed light on the behavior of the phosphoramidite ligands at the surface of the particles, a few drops of H_2_O_2_ were added separately into the mixture of nanoparticles and stirred vigorously overnight at room temperature since the phosphine oxide peak is not seen on the ^31^P NMR time scale. After this slow oxidation process, H_2_O_2_ led to the appearance of new phosphorus signals at around δ 12.86 ppm ((*S*)-1@PdNP), 13.40 ppm ((*S*)-2@PdNP), and 9.84 ppm ((*S*)-3@PdNP), which were attributed to oxidized phosphoramidite ligands, respectively. For comparison study, we also determined the ^31^P NMR spectra of oxide forms of the free phosphoramidite ligands (*S*)-1-3, which were easily prepared by the oxidation of the corresponding ligands with H_2_O_2_ in THF-d^8^ solution (Figures S20–S25). Thus, solution ^31^P NMR experiments provided clear evidence for the presence of phosphoramidite ligands at the nanoparticle surface with a strong interaction.

Thermogravimetric analysis (TGA) also confirmed that the presence of phosphoramidite ligands on the nanoparticle surface. On heating to 900 °C, (*S*)-1-3@PdNPs displayed %66.28, %66.38, and %69.32 mass losses which attributed to the reduction of organic matter, respectively. We also supported the palladium contents of each nanoparticle with ICP-MS analysis, and very similar results were obtained as compared to that obtained from TGA ((*S*)-1@PdNP, %33.6 Pd; (*S*)-2@PdNP, %33.5 Pd; (*S*)-3@PdNP, %30.8 Pd) (see the Supporting Information).

**Figure 4:**
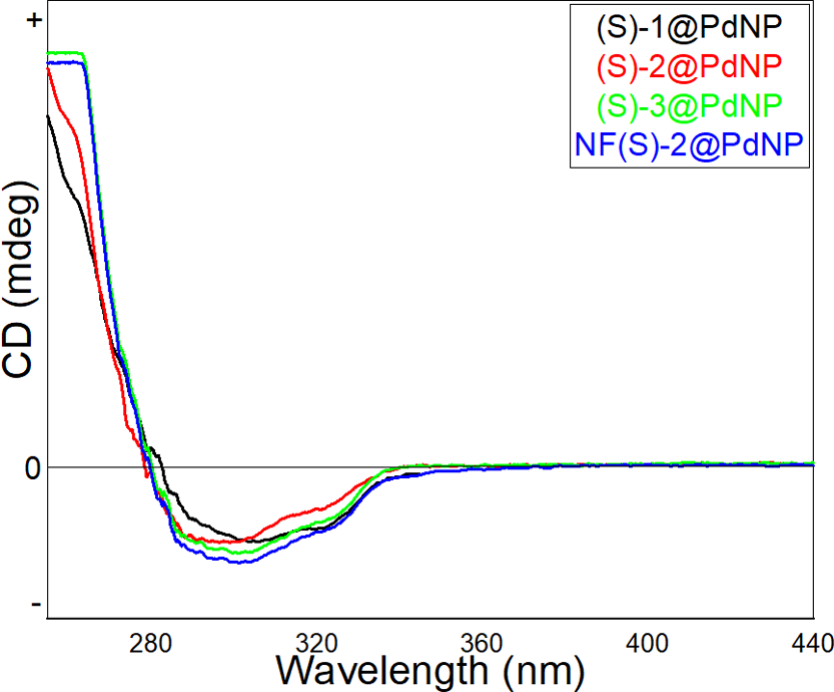
CD spectra of (*S*)-1-3@PdNPs and NF(*S*)-2@PdNP.

To explore the optical activity of the phosphoramidite stabilized-PdNPs, CD) spectra have been recorded. The CD spectra of (*S*)-1-3@PdNPs and NF(*S*)-2@PdNPs showed a negative Cotton effect, as shown in Figure 4. These results indicate that binaphthyl-based phosphoramidite stabilized-PdNPs were stable against racemization. Thus, it has been proven that new optically active PdNPs can be prepared by using enantiomerically pure phosphoramidite ligands in which the chiral center is very close to the surface of the palladium nanoparticles.

### 3.3 Asymmetric Suzuki C–C Coupling Reactions with Phosphoramidite Stabilized-PdNPs

The asymmetric synthesis of sterically hindered binaphthalenes is generally catalyzed by homogeneous chiral palladium complexes and often result in low enantioselectivity even at high temperatures, and prolonged reaction times are used. On the other hand, only one example was published (as mentioned above) for nonsupported chiral PdNP-catalyzed asymmetric synthesis of binaphthalene units. In that study, the best result (47–96% yield and 18–69% ee) was obtained with the use of (*S*)-binap-PdNPs (0.1 mol %) from a series of phosphine stabilized-palladium nanoparticles for the asymmetric coupling of 1-bromo-2-methoxynaphthalene and 1-naphthylboronic acid in the presence of Ba(OH)_2_ as a base and DME/H_2_O (9/1) as a solvent at room temperature for 3–24 h.^17^ Moreover, the authors did not report the reusability and recyclability studies of the catalysts, which have been known as essential properties for the heterogeneous catalytic systems. At this point, it should be strongly stated that although pioneering strides have been taken for the synthesis of the axially chiral biaryl compounds, the development of recyclable, reusable, and more importantly highly enantioselective heterogeneous catalyst systems with acceptable catalytic activities still remains a major challenge.

In this regard, to investigate the catalytic behavior of our phosphoramidite stabilized-palladium nanoparticles ((*S*)-1-3@PdNPs) in a comprehensive manner, the asymmetric Suzuki C-C coupling reaction between 2-ethoxynaphthaleneboronic acid with 1-iodonaphthalene promoted by chiral (*S*)-2@PdNP was chosen as the model reaction, and the optimum catalytic conditions were determined by the amount of the catalyst that was set as 3.0 mg (Table 2). The by-products of the reactions were not detected except for the condition using water as the cosolvent. A comparative catalytic study between nonfluorinated (NF(*S*)-2@PdNP) and perfluorinated phosphoramidite stabilized-PdNP ((*S*)-2@PdNP) was also described. As shown in entry 1–10, with the use of NaOAc and K_2_CO_3_ as bases in 1,4-dioxane, DME, or toluene solvent, which are commonly used in Pd-catalyzed coupling reactions, no cross-coupling product was observed within 1 h. However, in the presence of DMF, the yield of the cross-coupling product was significantly increased to 32 and 22% with 82 and 71% ee during the first hour of the reaction when K_2_CO_3_ and NaOAc bases were used, respectively (Entries 3 and 8, Table 2). Under these base-solvent combinations, further increasing the reaction time resulted in slightly higher yields (up to 53%) but lower ee values (Entries 4 and 9, Table 2). Surprisingly, the use of Cs_2_CO_3_ or KOH in DMF afforded the cross-coupling product with higher yields (41 and 66%) and more importantly >99% ee, while 69% yield could be obtained with the same enantioselectivity by using CsF as the base in 1 h (Entries 13, 17 and 19, Table 2). At this stage, the addition of water as a cosolvent to DMF, which might help dissolve CsF into the reaction mixture, produced lower catalytic activity (56% yield, entry 21, Table 2) probably due to the proto-deboronation of 2-ethoxynaphthaleneboronic acid during the reaction.^46^ On the other hand, no significant improvement in the yield of the cross-coupling product was obtained by further prolonging the reaction time from 1 to 3 h (Entry 20, Table 2). Similarly, by lowering the reaction temperature from 80 °C to room temperature decreased the product yield (Entry 22, Table 2). To further enhance the catalytic activity, the effect of the catalyst amount was also examined, and the yield of the cross-coupled product did not increase in the presence of higher catalyst loading either (Entry 23, Table 2). Hence, the combination of CsF and DMF indicated higher catalytic activity at 80 °C in the presence of 3.0 mg of the catalyst (approximately 1.0 mol % of the substrate) within only 1 h of the reaction (Entry 19, Table 2). It is noteworthy that full enantioselectivity was captured under these conditions. As an indication of the possible practical potential of this phosphoramidite stabilized-PdNP system, the reaction was scaled-up 25-fold under the optimized conditions, and upon purification of the 2-ethoxy-1,1′-binaphthalene using column chromatography, the resulting isolated yield (5.06 g, 68%) and enantiomeric purity (>99% ee) was found to be in good agreement with the HPLC analysis.

**Table 2:**
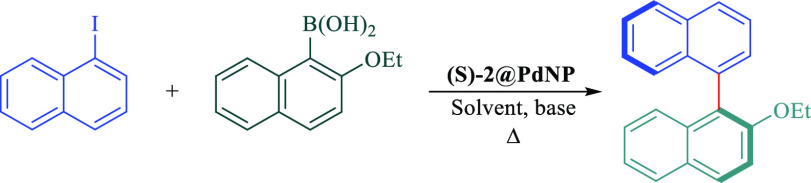
Optimization Studies of the Asymmetric Suzuki C–C Coupling Reaction between 1-Iodonaphthalene and 2-Ethoxynaphthaleneboronic Acid Using (*S*)-2@PdNPs^a^

entry	base	solvent	*T* (°C)	*t* (min.)	yield (%)^b^	ee (%)^c^
**1**	K_2_CO_3_	1,4-dioxane	80	60	<5	
**2**	K_2_CO_3_	DME	80	60	<5	
**3**	K_2_CO_3_	DMF	80	60	32	82
**4**	K_2_CO_3_	DMF	80	180	53	68
**5**	K_2_CO_3_	toluene	80	60	<5	
**6**	NaOAc	1,4-dioxane	80	60	<5	
**7**	NaOAc	DME	80	60	<5	
**8**	NaOAc	DMF	80	60	22	76
**9**	NaOAc	DMF	80	180	50	71
**10**	NaOAc	toluene	80	60	<5	
**11**	KOH	1,4-dioxane	80	60	42	86
**12**	KOH	DME	80	60	44	57
**13**	KOH	DMF	80	60	66	>99
**14**	KOH	DMF	80	180	52	>99
**15**	KOH	toluene	80	60	10	68
**16**	Ba(OH)_2_	DMF	80	60	31	80
**17**	Cs_2_CO_3_	DMF	80	60	41	>99
**18**	DIPEA	DMF	80	60	<5	
**19**	CsF	DMF	80	60	70	>99
**20**	CsF	DMF	80	180	71	>99
**21**	CsF	DMF + H_2_O (3:1)	80	60	56	>99
**22**	CsF	DMF	25	60	60	>99
**23**	CsF	DMF	80	60	72^d^	>99
**24**	CsF	DMF	80	60	70^e^	>99
**25**	CsF	DMF	80	60	<5^f^	
**26**	CsF	DMF	80	60	70^g^, 70^h^, 70^i^	>99^g^, >99^h^, >99^i^
**27**	CsF	DMF	80	60	57^j^, 35^k^	>99^j^, >99^k^

a Reaction conditions: 1-iodonaphthalene (1.0 mmol), 2-ethoxynaphthaleneboronic acid (1.5 mmol), base (3.0 mmol), solvent (4.0 mL), cat.: (*S*)-2@PdNP (3.0 mg).

b Yield of isolated product after column chromatography and from duplicated experiments.

c Determined by HPLC using the OD-H column as the mean of two runs.

d Cat. (10.0 mg).

e In the presence of >300 equiv Hg(0).

f In the presence of 0.5 equiv CS_2_.

g After the 2nd consecutive catalytic run.

h After the 5th consecutive catalytic run.

i After the 12th consecutive catalytic run.

j Cat.: NF(*S*)-2@PdNP (3.0 mg).

k After the 4th catalytic run when NF(*S*)-2@PdNP was used as the catalyst.

The heterogeneity of the catalyst (*S*)-2@PdNPs was checked by Hg(0) and CS_2_ poisoning experiments. These experiments were each started as if they were a standard condition of asymmetric Suzuki C–C coupling reaction between 2-ethoxynaphthaleneboronic acid with 1-iodonaphthalene. In a typical poisoning experiment, an excess of Hg(0) (300 equiv) or 0.5 equiv CS_2_ was added to the reaction mixture at the beginning of the reaction. However, the addition of excess Hg(0) to the reaction medium did not show any suppression of the catalytic activity, and 70% yield of the product and >99% ee was observed after 1 h (Entry 24, Table 2). This result clearly suggests that Hg(0) can only interact with ″naked″ species of Pd(0) which are not protected by strong ligands, by amalgamating or adsorbing to the metal surface; thereby, it can suppress the catalysis process. Furthermore, the reaction proceeded with full enantioselectivity that demonstrated the interesting superior ability of phosphoramidite ligands to protect the palladium surface. On the other hand, since the Hg(0) poisoning test alone will not be sufficient to determine whether the process is homogeneous or heterogeneous, the CS_2_ poisoning test is also performed. The addition of 0.5 equiv CS_2_ completely suppressed the formation of 2-ethoxy-1,1’-binaphthalene after 1 h (Entry 25, Table 2), suggesting that the catalyst has a heterogeneous nature. In addition, the hot filtration test was also performed in the middle of the reaction, and it was observed that no reaction proceeded in the filtrate, resulting from the homogeneous catalysis of the catalytically active species. Thus, clear evidence was obtained with both poisoning experiments and the hot filtration test, showing that phosphoramidite stabilized-PdNPs act as heterogeneous catalysts for asymmetric Suzuki coupling reactions.

The catalyst lifetime is an important point for the heterogeneous systems; therefore, the stability of the (*S*)-2@PdNPs was also investigated in detail. To test the reusability of the catalyst, a series of consecutive runs were performed using the same catalyst sample. After 12 times of repeated asymmetric coupling of 2-ethoxynaphthaleneboronic acid with 1-iodonaphthalene under the optimized conditions, no significant decline in activity (70%) and enantioselectivity (>99% ee) was found (Entry 26, Table 2). The leaching of palladium was determined by ICP-MS measurements at the 2nd, 4th and 12th cycles and found to be no more than %0.0064 (16.3 ppm), 0.00028% (7.05 ppm), and 0.00021% (48.9 ppm), respectively. Moreover, TEM micrographs recorded after the relevant cycles showed no change in the particle size, and no evidence of significant aggregation was observed, which clearly suggests that (*S*)-2@PdNPs are highly stable (Figure S27). To be able to compare between nonfluorinated and perfluorinated phosphoramidite stabilized-PdNPs, the NF(*S*)-2@PdNPs were also investigated for recoverability and reusability over four catalytic runs (Entry 27, Table 2). Excitingly, it was observed that full enantioselectivity (>99%) was still maintained after the fourth run, but the product yield is decreased from 57 to 35%. The partial loss of palladium may be mainly responsible for the non-negligible degradation in the catalytic activity of the NF(*S*)-2@PdNPs. We therefore conducted the hot filtration test, and subsequent ICP-MS analysis of the filtrates after every cycle showed that the leaching of palladium from the heterogeneous catalyst surface is not insignificant. However, the palladium content in the fourth-generation of the NF(*S*)-2@PdNPs detected by ICP-MS analysis was dropped to 10.6% compared to the original Pd content (32.7%, from TGA analysis, Figure S26). The above observations point out that the presence of perfluorinated ponytails on the chiral binaphthyl-based phosphoramidite stabilizer, providing further interaction on the nanoparticle surface during the catalytic reactions, contributes to the overall stability without hindering their enantioselectivity.

To illustrate the general catalytic behavior of our phosphoramidite stabilized chiral PdNPs, we also investigated the asymmetric Suzuki C–C coupling of several aryl and naphthyl boronic acids and naphthyl halides having different steric substituents (Table 3). The axially chiral phenylnaphthalene derivatives 2-methoxy-1-(o-tolyl)naphthalene and 2-ethoxy-1-(o-tolyl)naphthalene were prepared by the coupling of corresponding naphthylbromides with o-tolylboronic acid. In all cases, the coupling reactions proceeded with good yields (48–74%) and moderate (10–52% ee) enantioselectivities (Entries 1 and 2, Table 3). When 2-methylnaphthaleneboronic acid was used for cross-coupling with the same naphthylbromides, axially chiral binaphthalene products were isolated in 55–66% yield with 28–66% ee (Entries 3 and 4, Table 3). In Sawai’s work, chiral 2-methyl-1,1′-binaphthalene was obtained in 44% yield with 60% ee within 44 h of the reaction in the presence of the chiral (*S*)-binapPdNP catalyst.^17^ In this work, the same cross-coupling product could be prepared with a higher yield (47%) and excellent enantioselectivity (84% ee) in just 1 h of the reaction with catalyst (*S*)-1@PdNP (Entry 6, Table 3). In addition, when 1-iodonaphthalene, which has lower bond dissociation energy on C–X bonds was used as naphthyl halide, the reaction yield and ee were greatly improved. For example, by using (*S*)-1@PdNP and (*S*)-2@PdNP as the catalyst, a variety of chiral binaphthalenes was obtained with excellent yields (60–85%) and full enantioselectivity (>99% ee), while the same yield (59–85%) and high enantioselectivity (>96% ee) were obtained for (*S*)-3@PdNP (entries 7–9, Table 3). Similarly, higher yields and ees were obtained employing 1-iodo-2-methoxynaphthalene instead of 1-bromo-2-methoxynaphthalane as the substrate (compare entries 3 and 5, Table 3). These demonstrate that the iodo-substituted substrates are beneficial to achieve high enantioselectivities with significantly high catalytic activities in this reaction. The results thus indicate that binaphthyl based-phosphoramidite ligands are very efficient stabilizers for the preparation of highly active chiral PdNPs in asymmetric Suzuki coupling reactions, especially in terms of enantioselectivity and recycling performance.

**Table 3:**
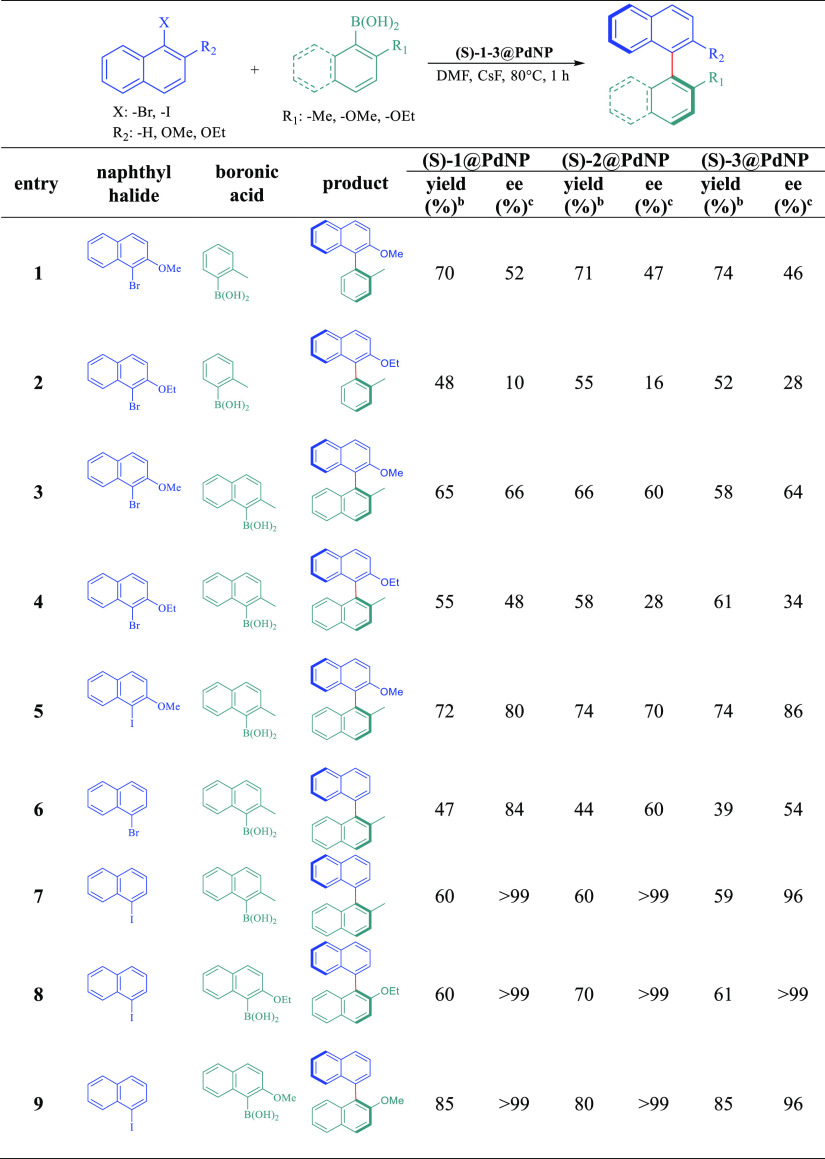
Typical Substrate Scope of Asymmetric Suzuki C–C Coupling Reactions Using (*S*)-1-3@PdNPs^a^

a Reaction conditions: Naphthyl halide (1.0 mmol), boronic acid (1.5 mmol), CsF (3.0 mmol), DMF (4.0 mL), catalyst (3.0 mg).

b Yield of isolated product after column chromatography.

c Determined by chiral HPLC analyses.

## 4 Con**c**lusions

In summary, we reported the first use of enantiopure binaphthyl-based phosphoramidite ligands as a stabilizer for the preparation of chiral PdNPs. The resulting chiral PdNPs with their new ensemble of high enantioselective entities catalyzed asymmetric Suzuki C–C coupling reactions with up to >99 ee for a very short time, acting as an efficient and excellent reusable catalyst for the synthesis of sterically hindered biaryls. The extremely long catalytic life and high enantioselectivity of these NPs point out their potentials for use as catalysts in many other asymmetric organic transformations.

## Supporting Information Available

NMR spectra; HRMS spectra; HPLC chromatograms; TEM images; and TGA spectra (PDF)
