# Supplementary material for: Highly Enantioselective Binaphthyl-Based Chiral Phosphoramidite Stabilized-Palladium Nanoparticles for Asymmetric Suzuki C–C Coupling Reactions
Source: Inorg Chem. 2023 Mar 6;62(11):4637–47. doi: 10.1021/acs.inorgchem.3c00079 (PMC10031557; doi:10.1021/acs.inorgchem.3c00079)
Supplement: Supplementary file 1 — ic3c00079_si_001.pdf [file ic3c00079_si_001.pdf]

Supporting Information

Highly Enantioselective Binaphthyl-based Chiral Phosphoramidite Stabilized-Palladium Nanoparticles for Asymmetric Suzuki C-C Coupling Reactions

Simay İnce [a], Özlem Öner [b], Mustafa Kemal Yılmaz [a,b,*], Mustafa Keleş [c], Bilgehan Güzel [d]

[a] Mersin University, Institute of Science, Department of Nanotechnology and Advanced Materials, 33343 Mersin, TURKEY

[b] Mersin University, Science Faculty, Department of Chemistry, 33343 Mersin, TURKEY

[c] Osmaniye Korkut Ata University, Faculty of Arts and Sciences, Department of Chemistry, 80000 Osmaniye, TURKEY

[d] Çukurova University, Faculty of Arts and Sciences, Department of Chemistry, 01330 Adana, TURKEY

* Corresponding author Tel.: +90 324 361 00 01; fax: 90 324 361 00 73
e-mail: mkyilmaz@mersin.edu.tr (Yılmaz MK)

Table of contents

1. NMR spectra of phosphoramidite ligands 3

2. HRMS data of phosphoramidite ligands 9

3. HPLC chromatograms for ligands 10

4. ^{31}P NMR spectra of PdNPs and oxide forms of ligands 11

5. TGA data 14

6. TEM images of PdNPs after recycling 16

7. HPLC chromatograms for the asymmetric Suzuki C-C coupling reactions 16

1. NMR spectra of phosphoramidite ligands

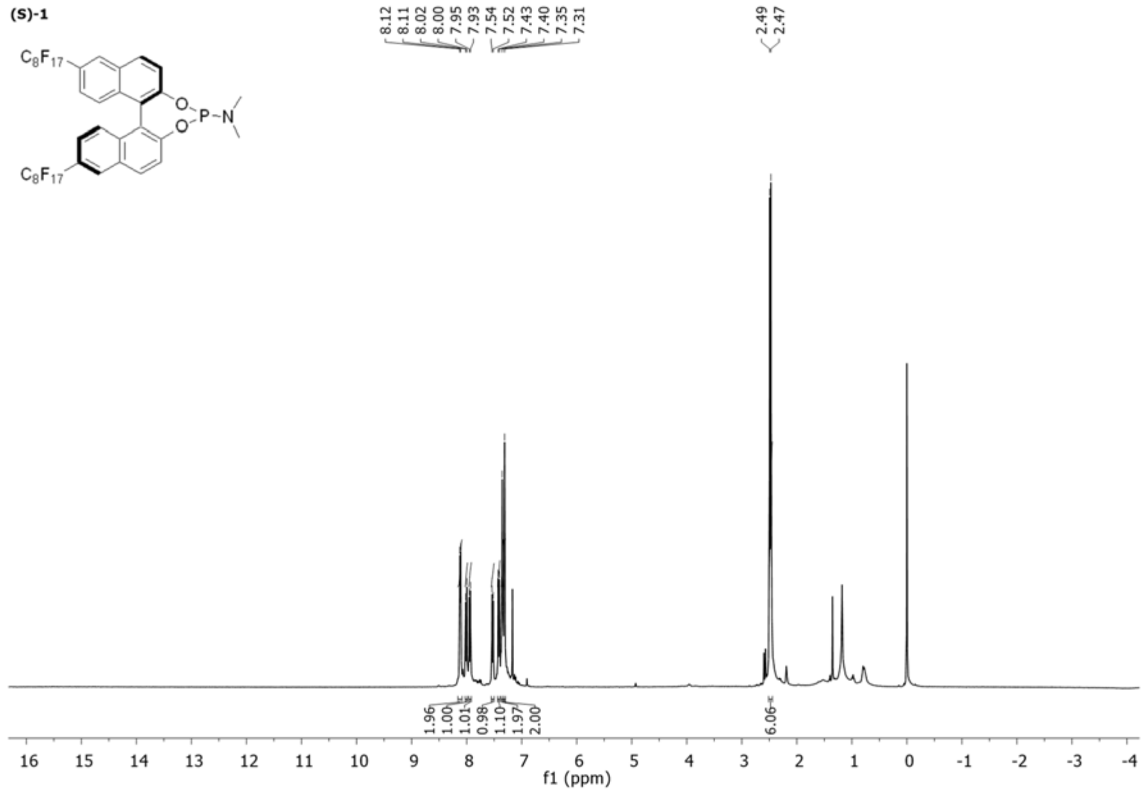

Figure S1: ^{1}H NMR spectra of ligand (S)-1

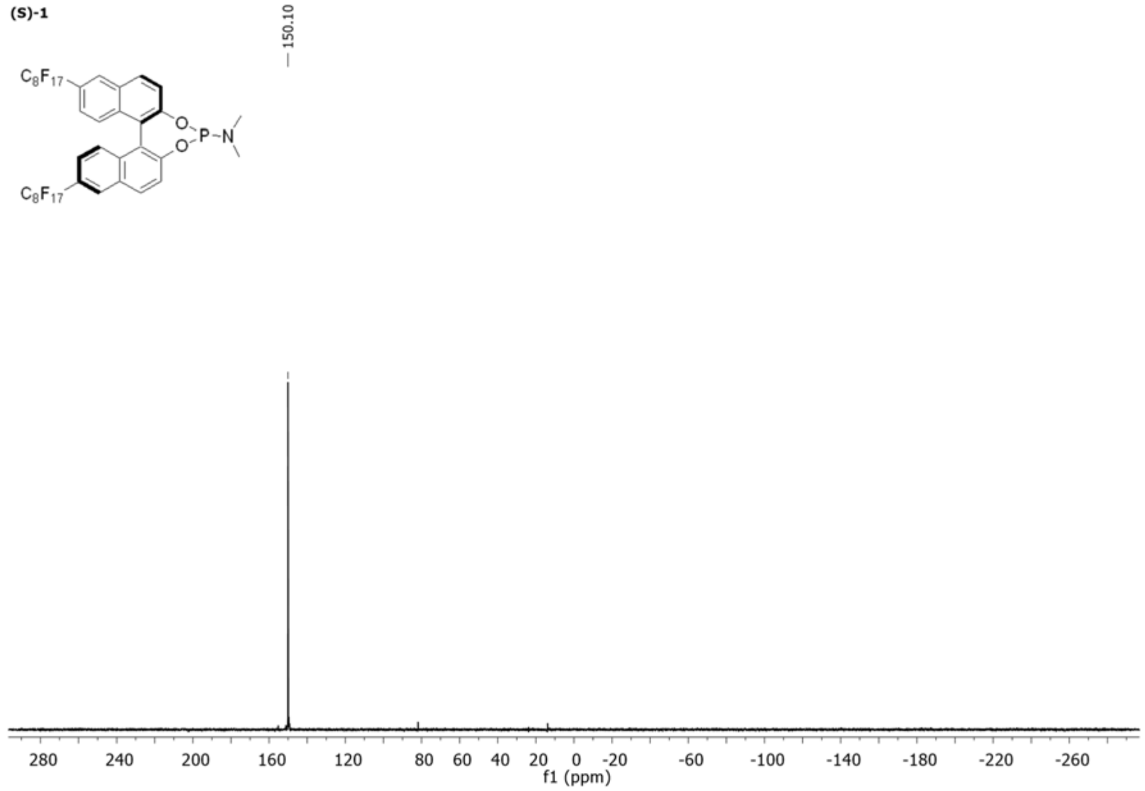

Figure S2: ^{31}P NMR spectra of ligand (S)-1

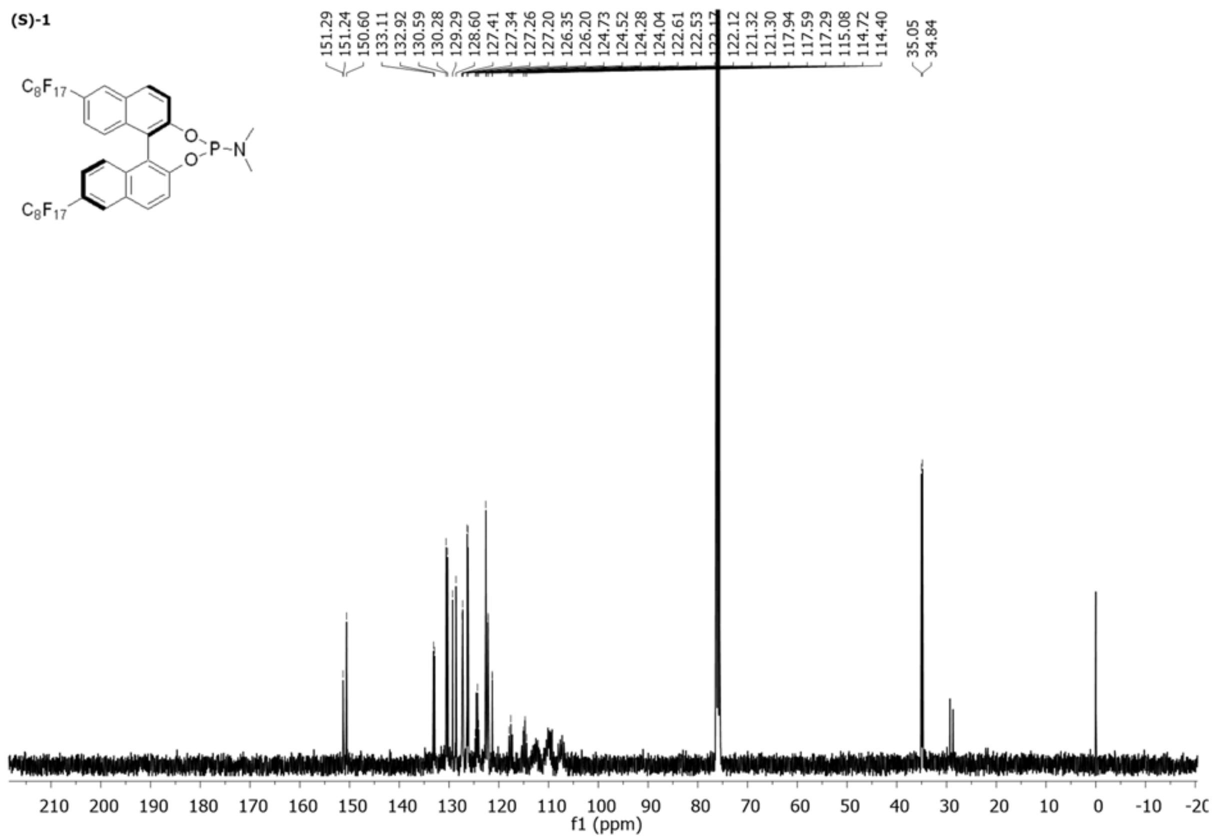

Figure S3: ^{13}C NMR spectra of ligand (S)-1

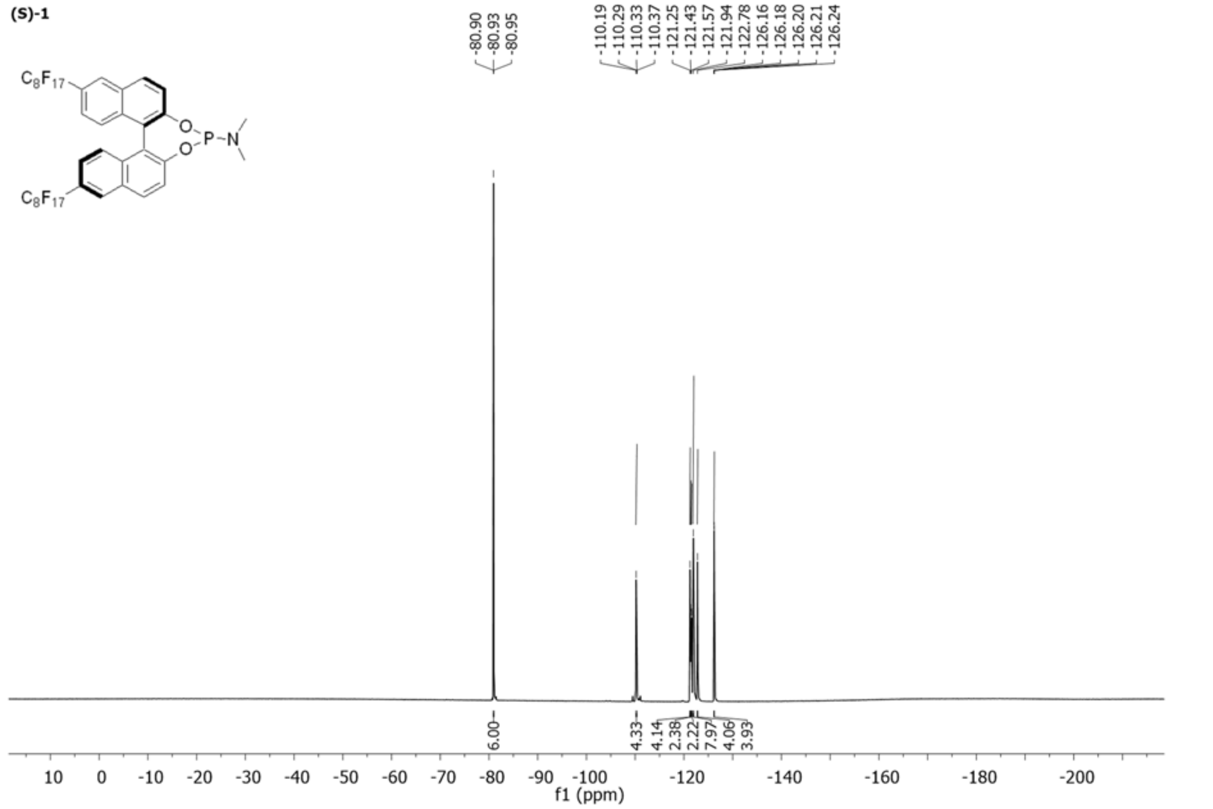

Figure S4: ^{19}F NMR spectra of ligand (S)-1

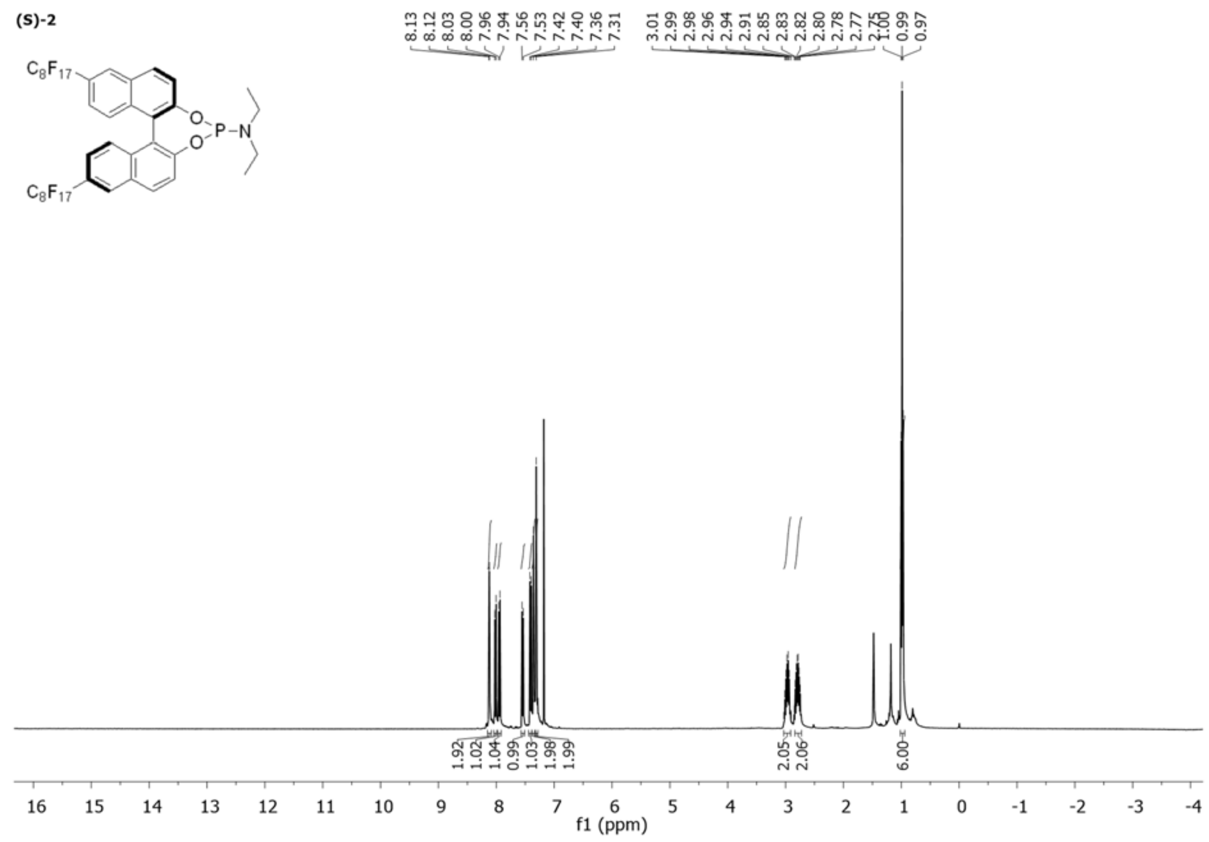

Figure S5: ^{1}H NMR spectra of ligand (S)-2

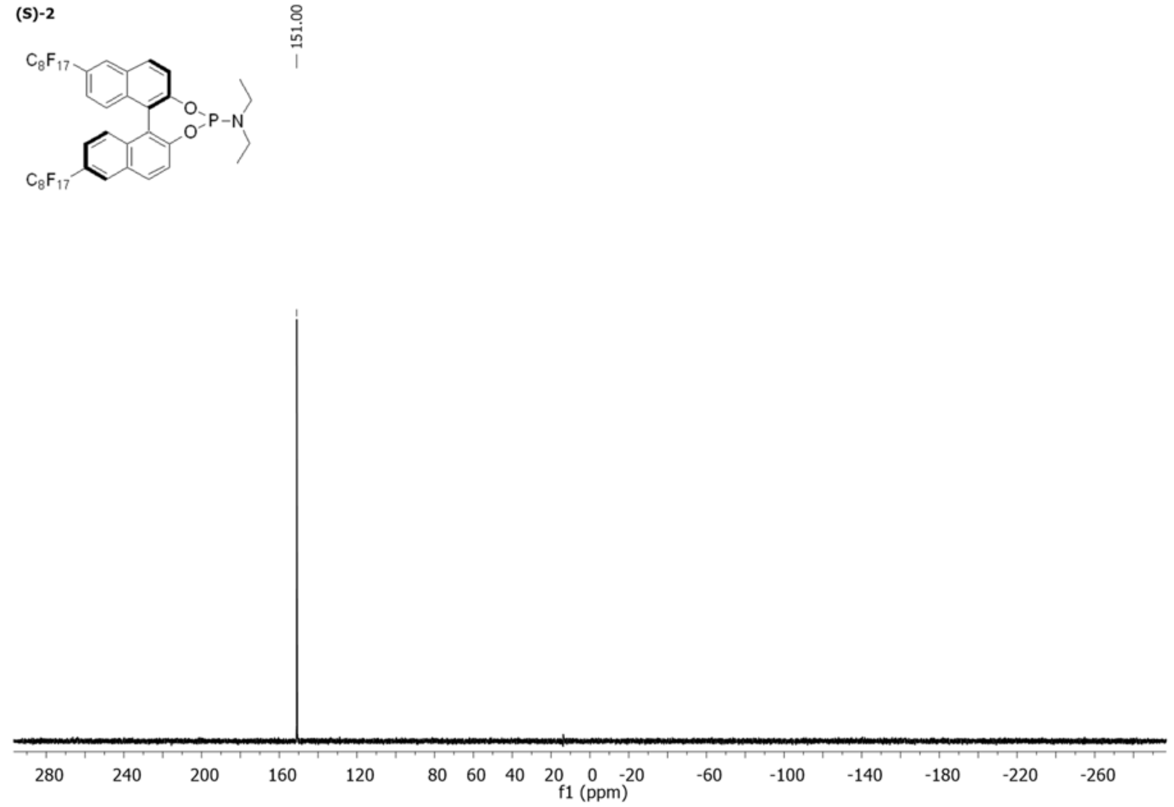

Figure S6: ^{31}P NMR spectra of ligand (S)-2

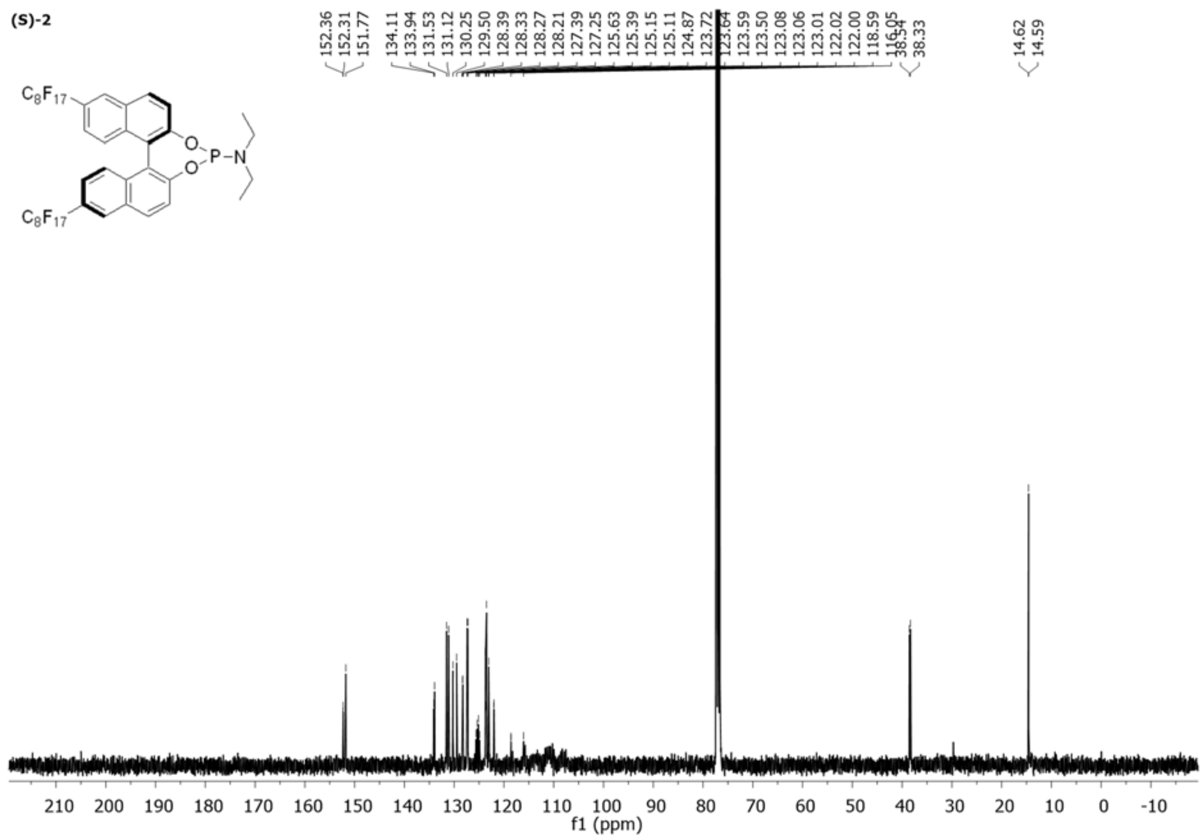

Figure S7: ^{13}C NMR spectra of ligand (S)-2

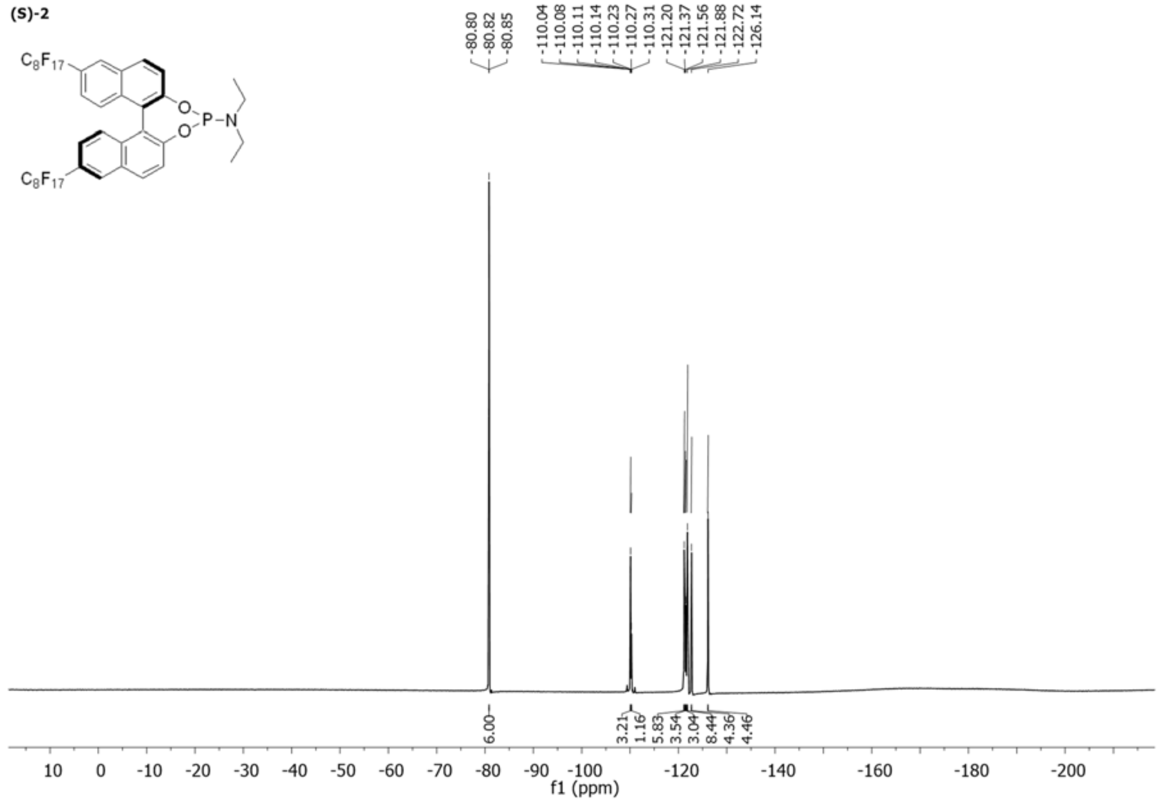

Figure S8: ^{19}F NMR spectra of ligand (S)-2

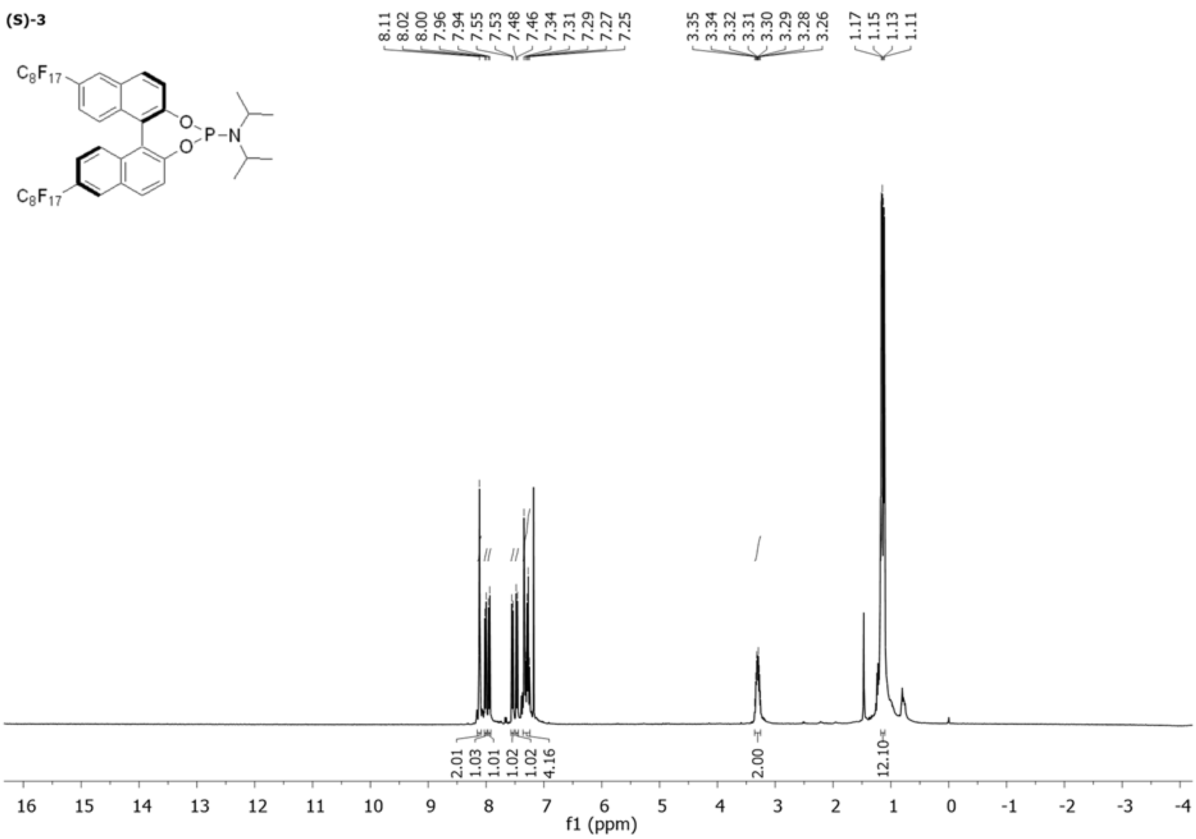

Figure S9: ^{1}H NMR spectra of ligand (S)-3

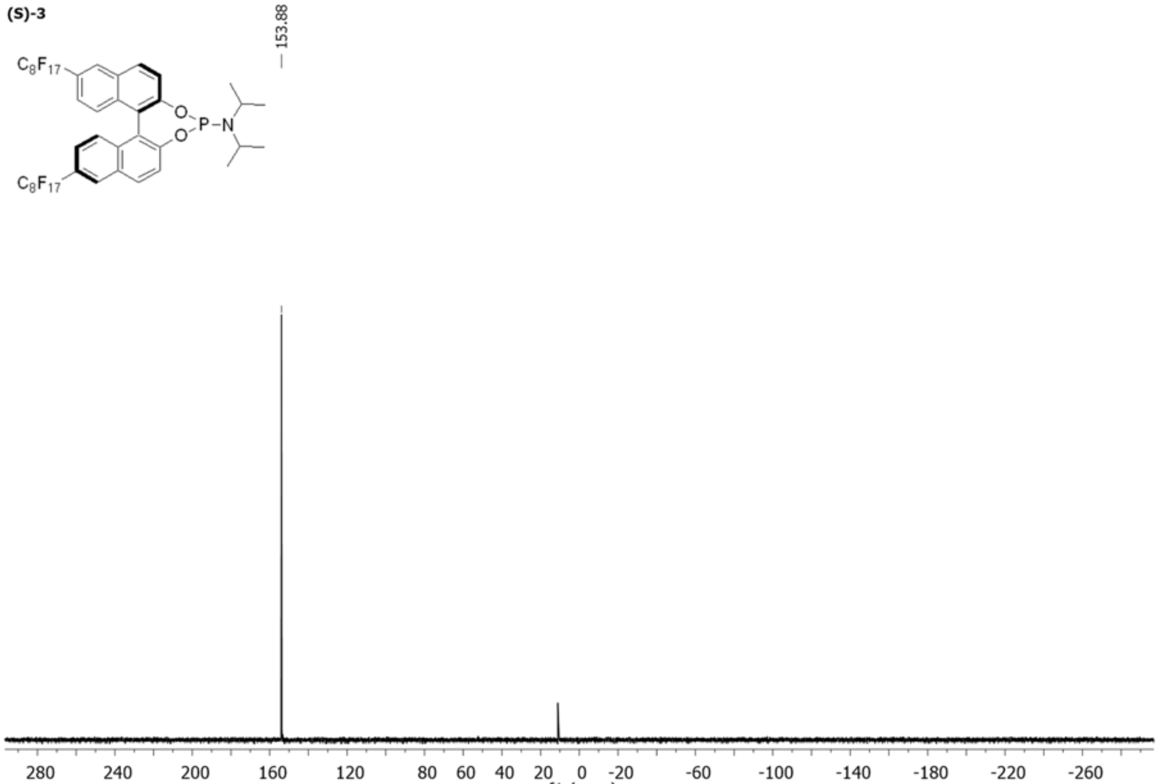

Figure S10: ^{31}P NMR spectra of ligand (S)-3

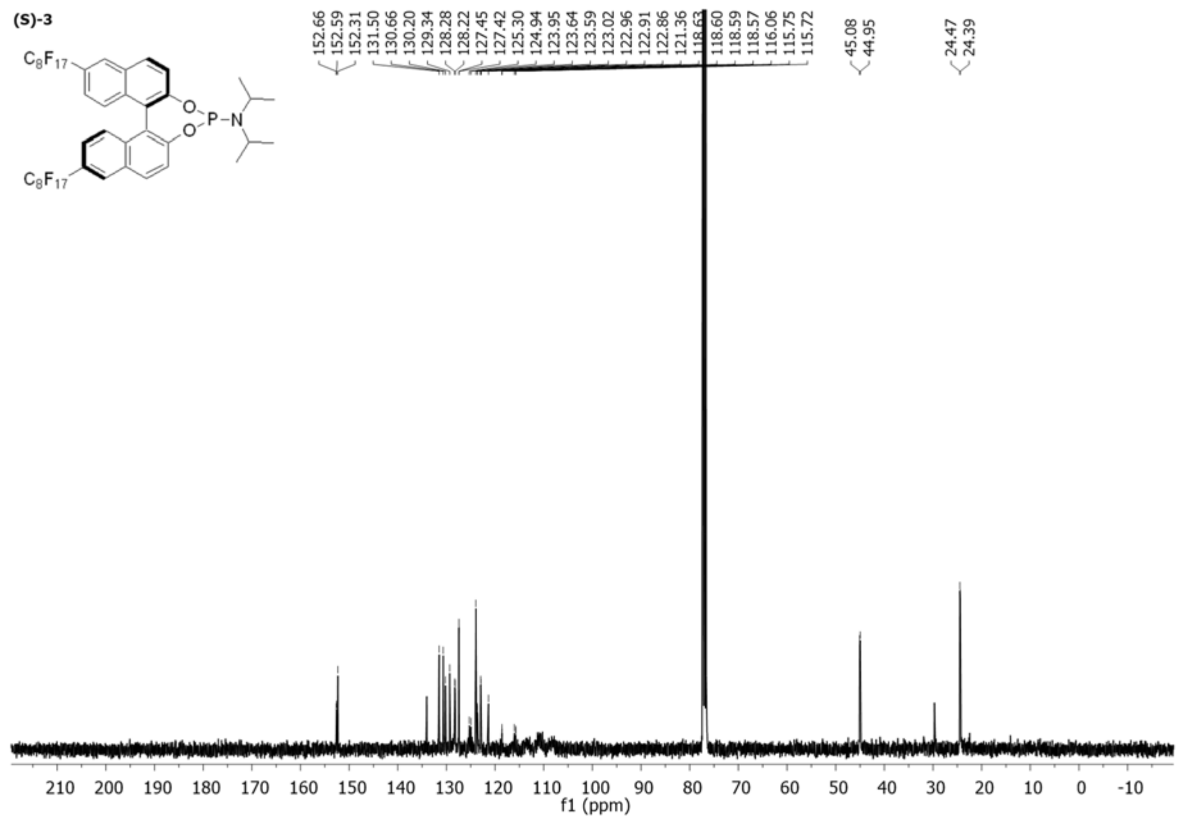

Figure S11: ^{13}C NMR spectra of ligand (S)-3

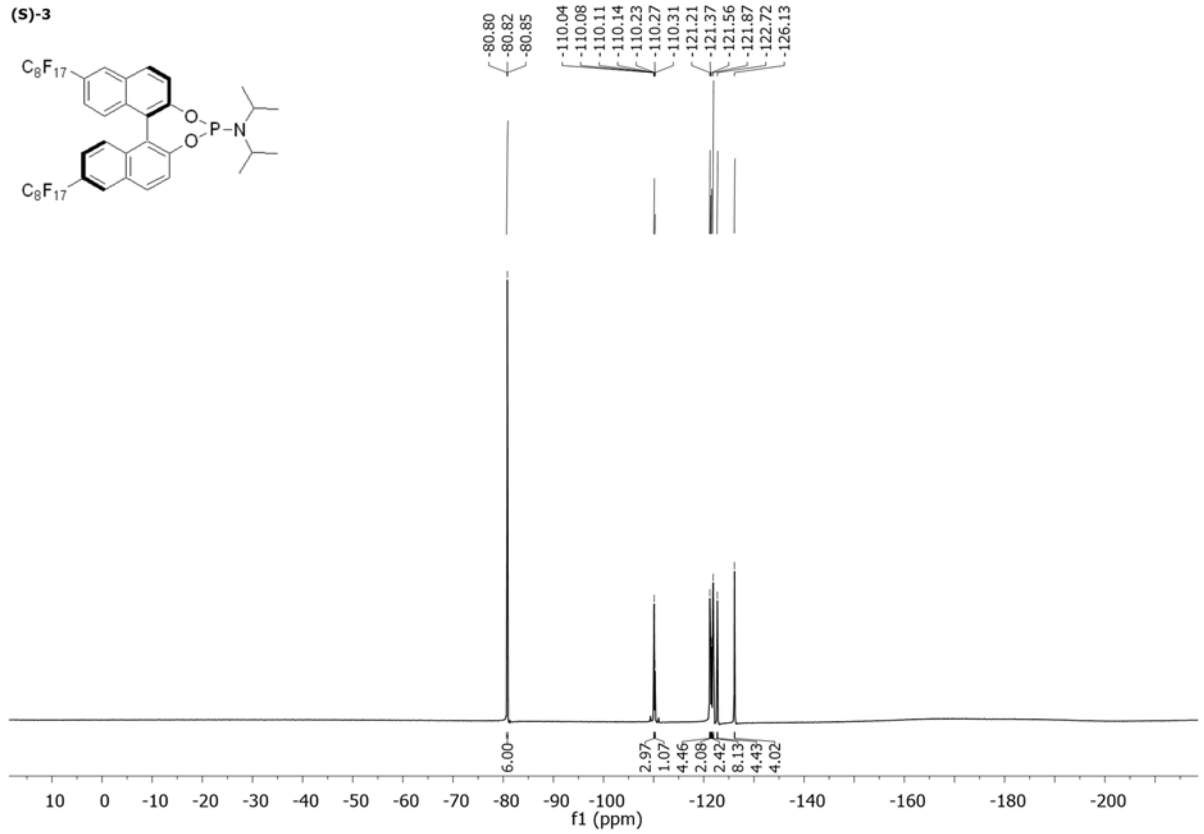

Figure S12: ^{19}F NMR spectra of ligand (S)-3

2. HRMS data of phosphoramidite ligands

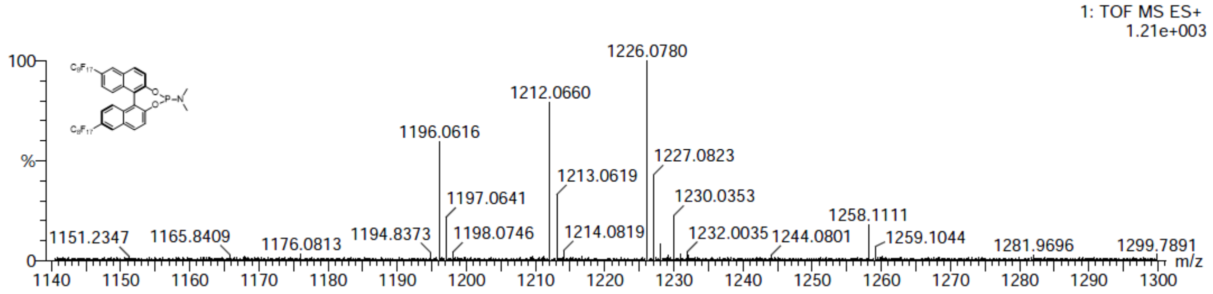

Minimum:				-5.5			
Maximum:		1000.0	5000.0	1000.0			
Mass	Calc. Mass	mDa	PPM	DBE	i-FIT	i-FIT (Norm)	Formula
1196.0616	1196.0454	16.2	13.5	14.5	136.4	0.0	C38 H17 N O2 F34 P

Figure S13: HRMS data of ligand (S)-1

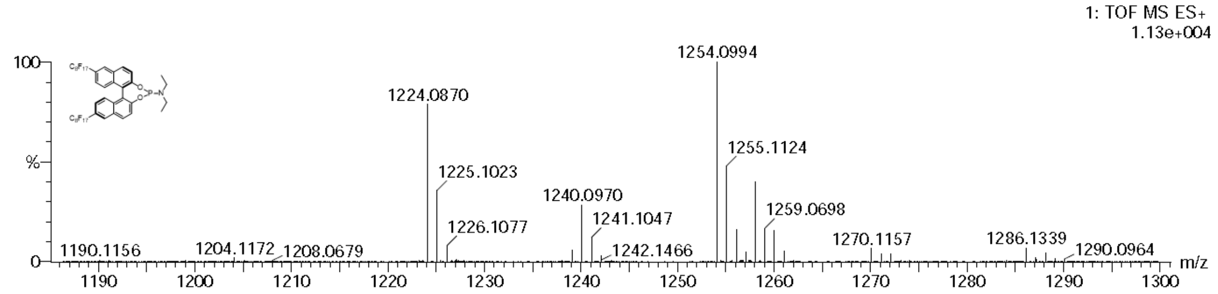

Minimum:				-5.5			
Maximum:		1000.0	5000.0	1000.0			
Mass	Calc. Mass	mDa	PPM	DBE	i-FIT	i-FIT (Norm)	Formula
1224.0870	1224.0767	10.3	8.4	14.5	79.9	0.0	C40 H21 N O2 F34 P

Figure S14: HRMS data of ligand (S)-2

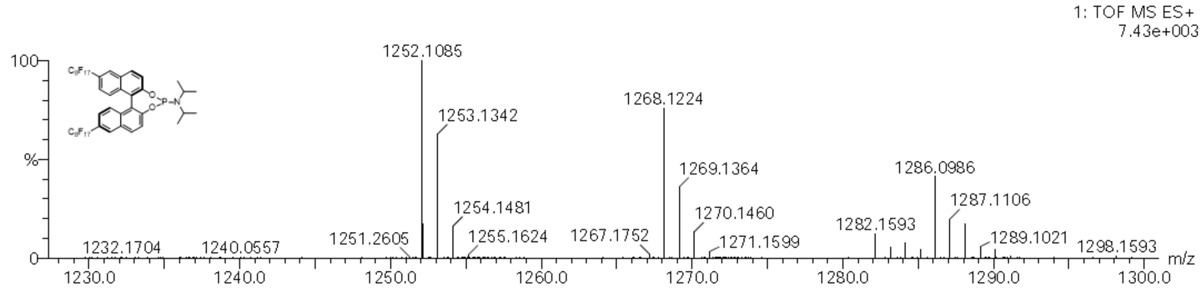

Minimum:				-5.5			
Maximum:		1000.0	5000.0	1000.0			
Mass	Calc. Mass	mDa	PPM	DBE	i-FIT	i-FIT (Norm)	Formula
1252.1085	1252.1080	0.5	0.4	14.5	96.3	0.0	C42 H25 N O2 F34 P

Figure S15: HRMS data of ligand (S)-3

3. HPLC chromatograms for ligands

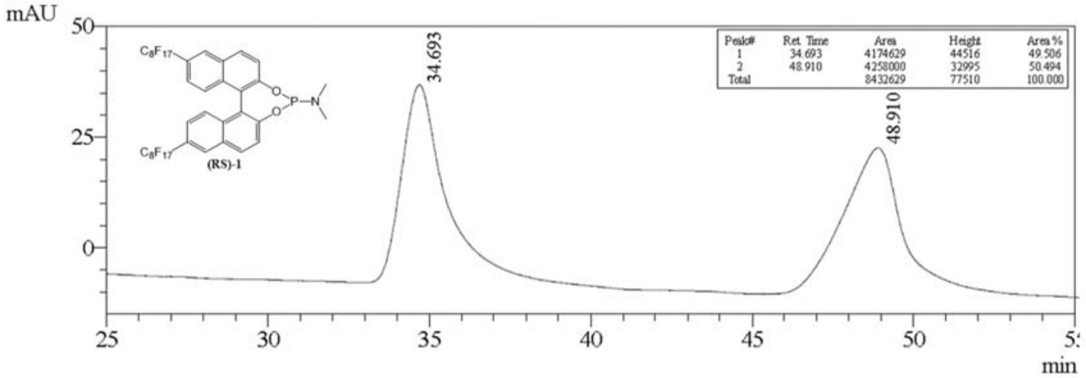

Figure S16: HPLC spectra for (RS)-*O,O*-(6,6'-diperfluorooctyl-1,1'-dinaphthyl-2,2'-diyl)-*N,N*-dimethylphosphoramidite ((RS)-1) ligand (Chiralcel OJ-H, 25°C, 0.3 mL/min, 90:10 n-hexane: i-PrOH, 335 nm).

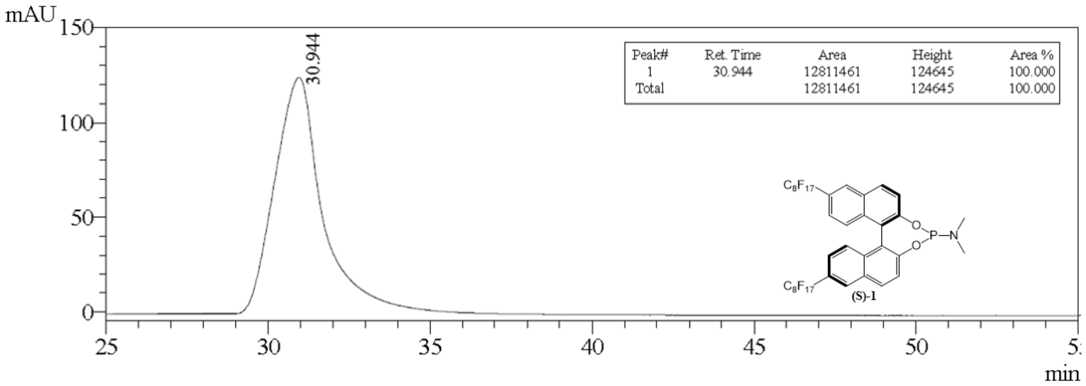

Figure S17: HPLC spectra for (S)-*O,O*-(6,6'-diperfluorooctyl-1,1'-dinaphthyl-2,2'-diyl)-*N,N*-dimethylphosphoramidite ((S)-1) ligand (Chiralcel OJ-H, 25°C, 0.3 mL/min, 90:10 n-hexane: i-PrOH, 335 nm).

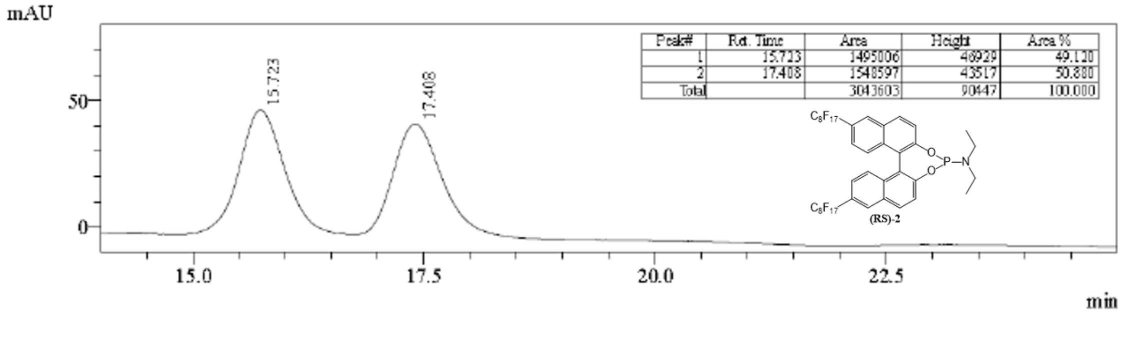

Figure S18: HPLC spectra for (RS)-*O,O*-(6,6'-diperfluorooctyl-1,1'-dinaphthyl-2,2'-diyl)-*N,N*-diethylphosphoramidite ((RS)-2) ligand (Chiralcel OJ-H, 25 °C, 0.3 mL/min, 95:5 n-hexane: i-PrOH, 330 nm).

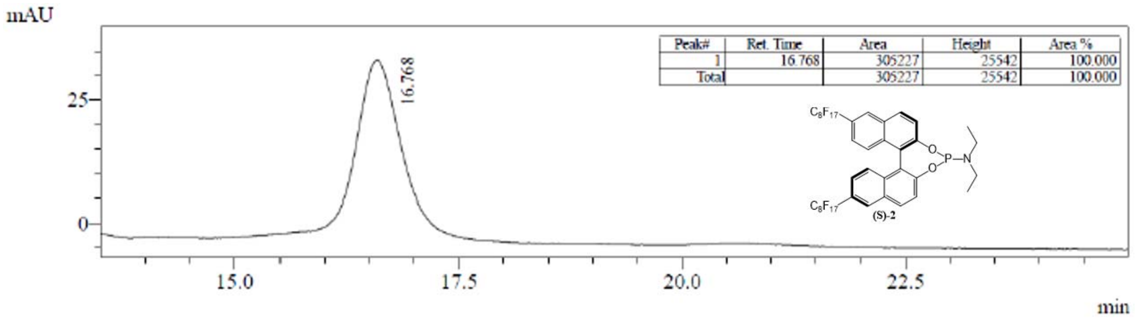

Figure S19: HPLC spectra for (S)-*O,O*-(6,6'-diperfluorooctyl-1,1'-dinaphthyl-2,2'-diyl)-*N,N*-diethylphosphoramidite ((S)-2) ligand (Chiralcel OJ-H, 25 °C, 0.3 mL/min, 95:5 n-hexane: i-PrOH, 330 nm).

4. ^{31}P NMR spectra of PdNPs and oxide forms of ligands

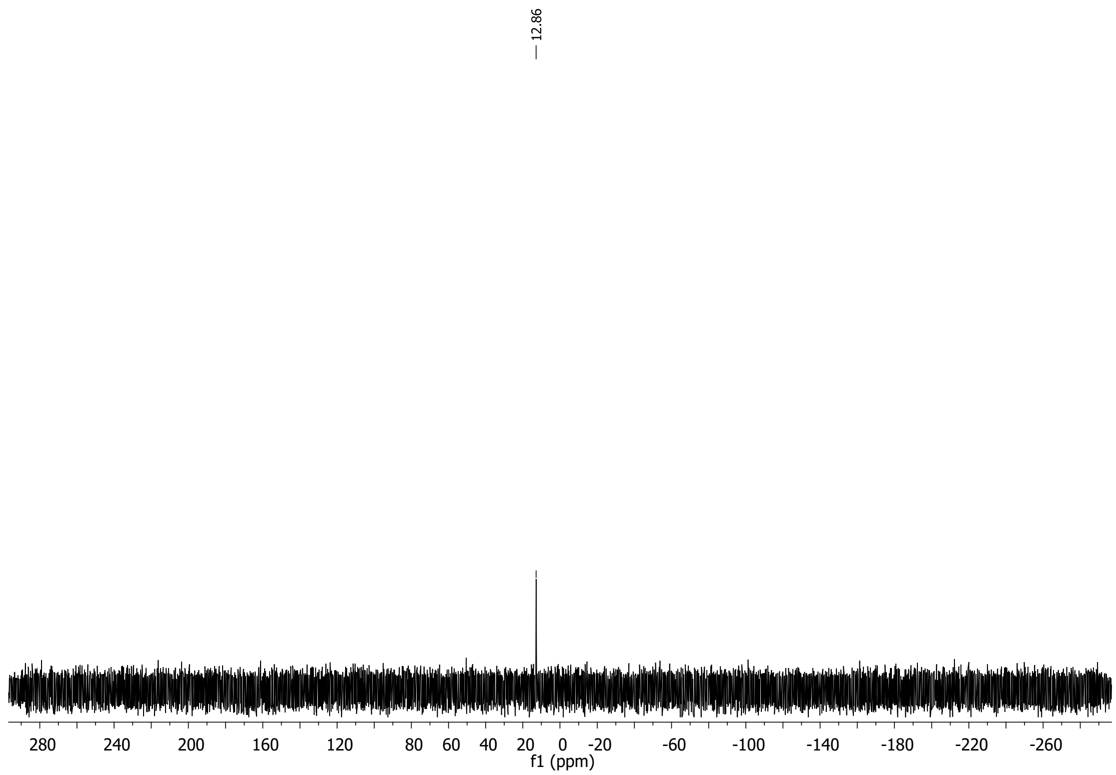

Figure S20: ^{31}P NMR spectra of (S)-1@PdNP after the addition of a few drops H_2O_2 and stirring overnight

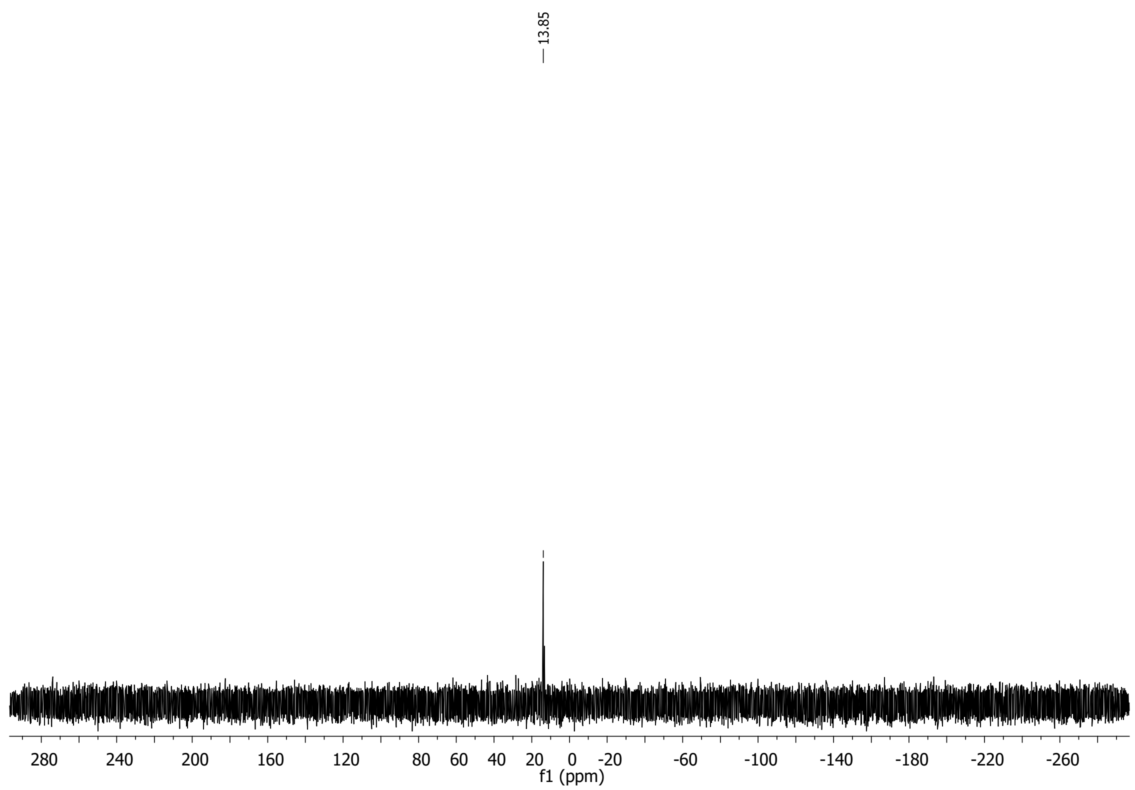

Figure S21: ^{31}P NMR spectra of (S)-1 oxide form after the H_2O_2 addition to the ligand solution

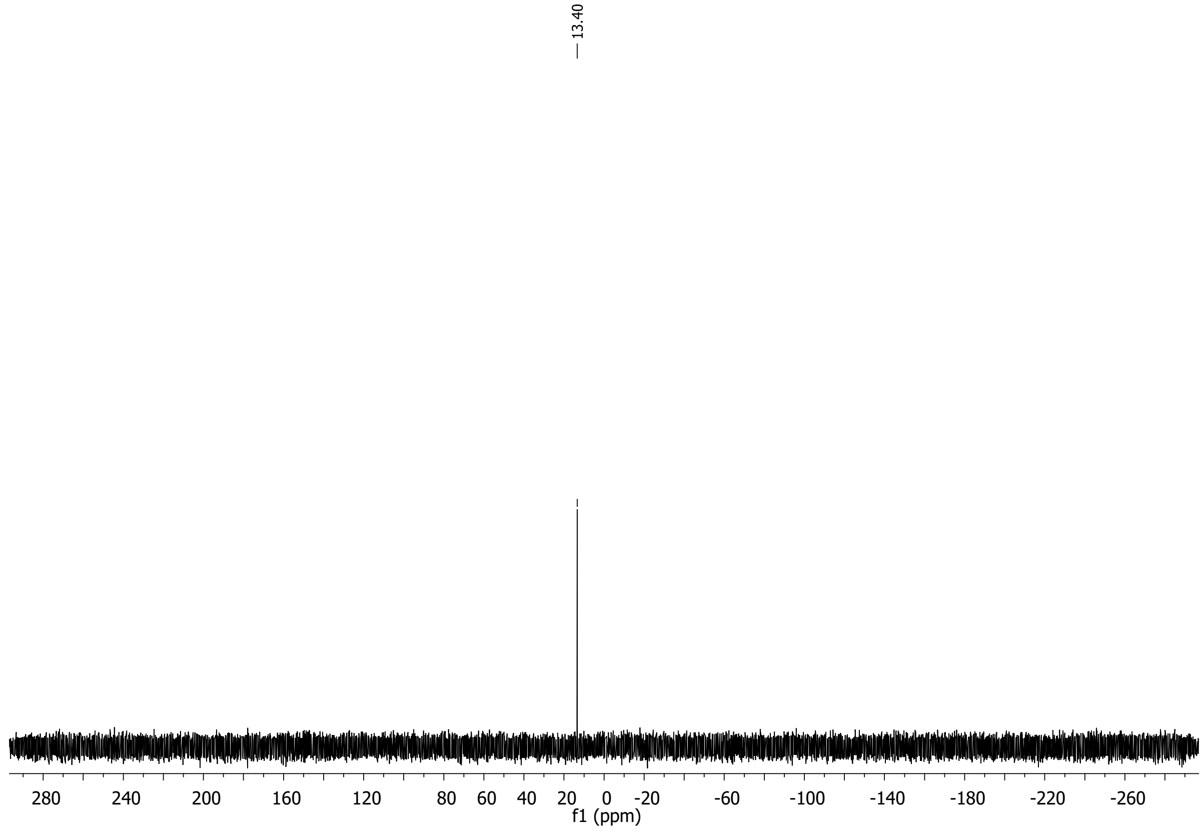

Figure S22: ^{31}P NMR spectra of (S)-2@PdNP after the addition of a few drops H_2O_2 and stirring overnight

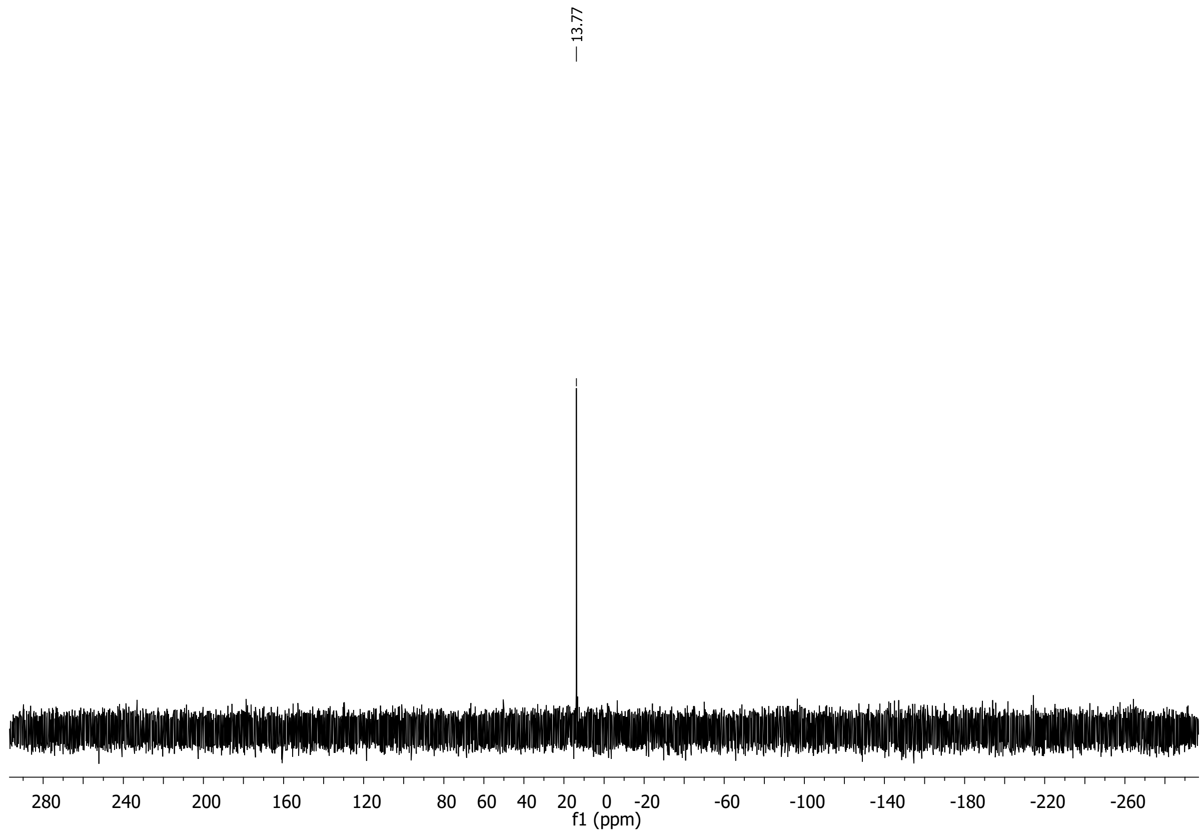

Figure S23: ^{31}P NMR spectra of (S)-2 oxide form after the H_2O_2 addition to the ligand solution

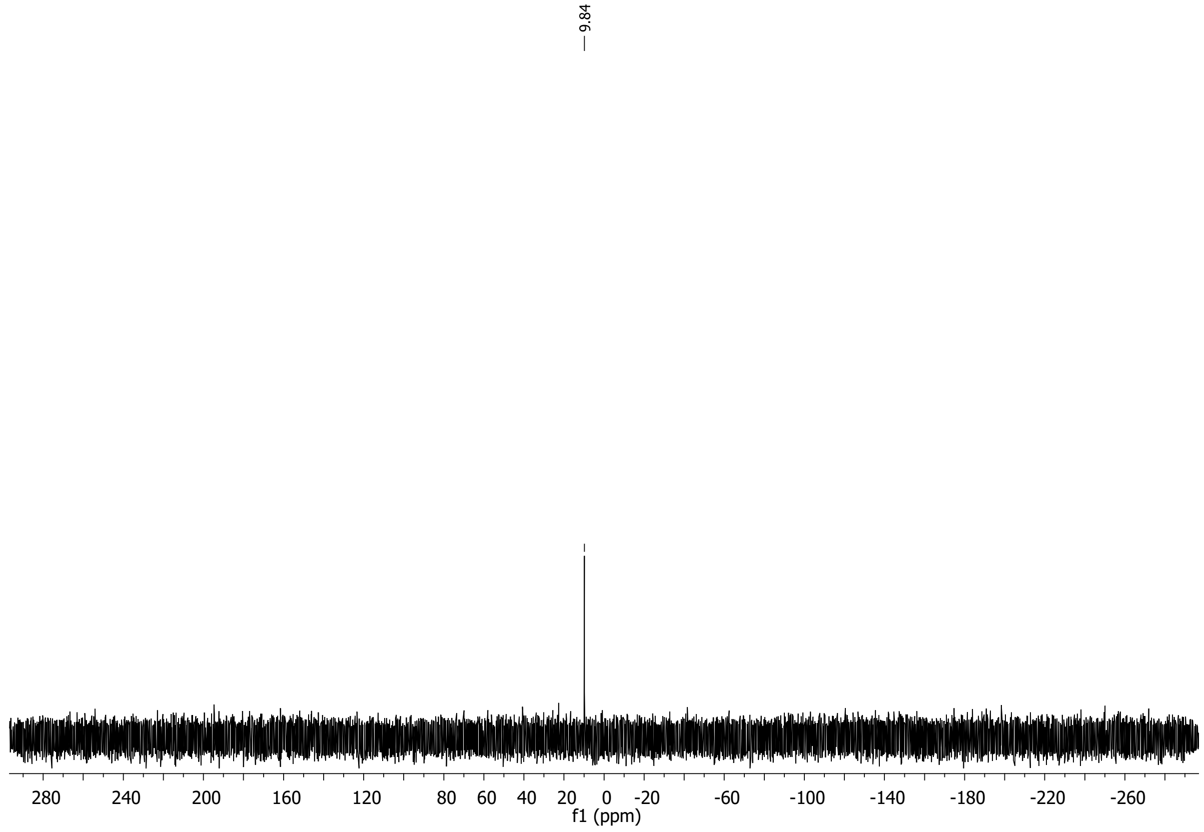

Figure S24: ^{31}P NMR spectra of (S)-3@PdNP after the addition of a few drops H_2O_2 and stirring overnight

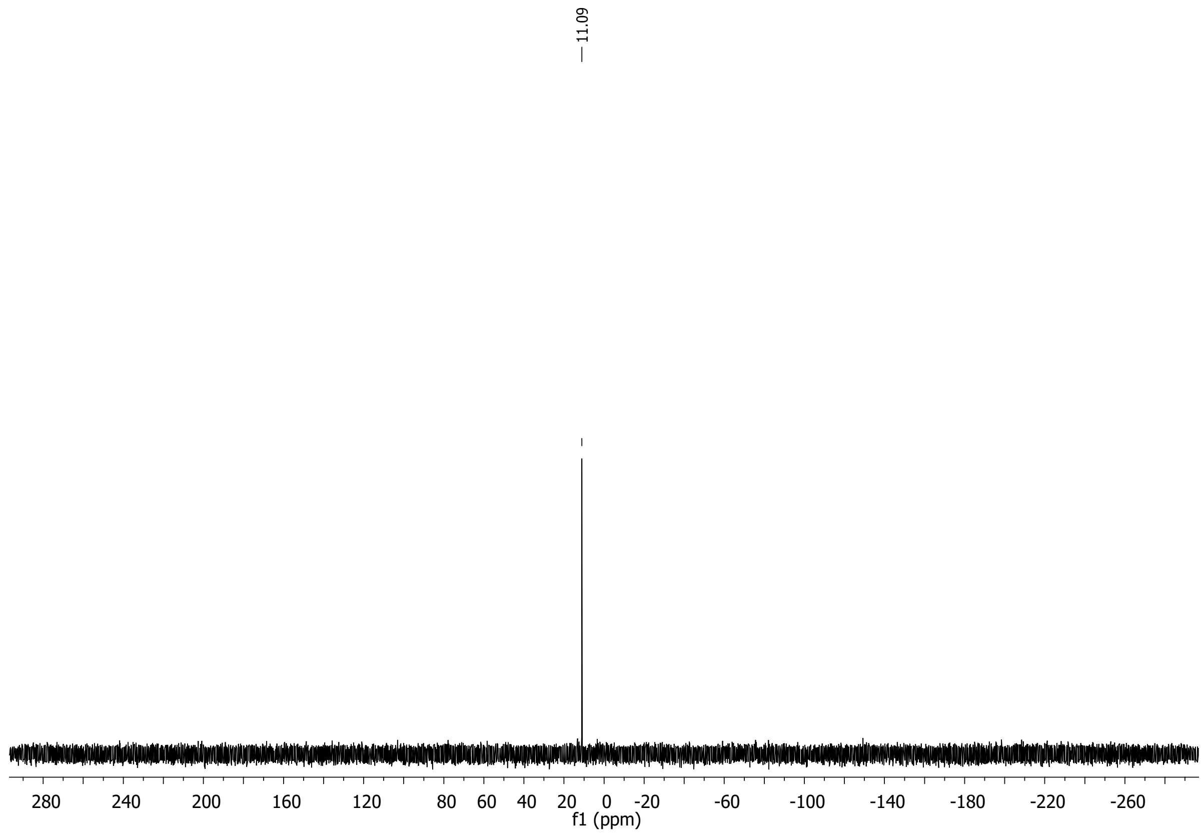

Figure S25: ^{31}P NMR spectra of (S)-3 oxide form after the H_2O_2 addition to the ligand solution

5. TGA data

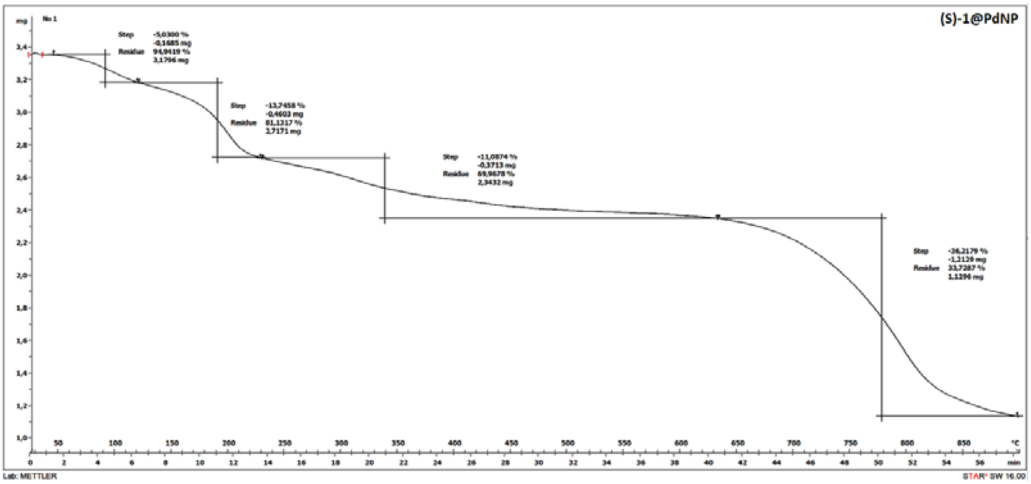

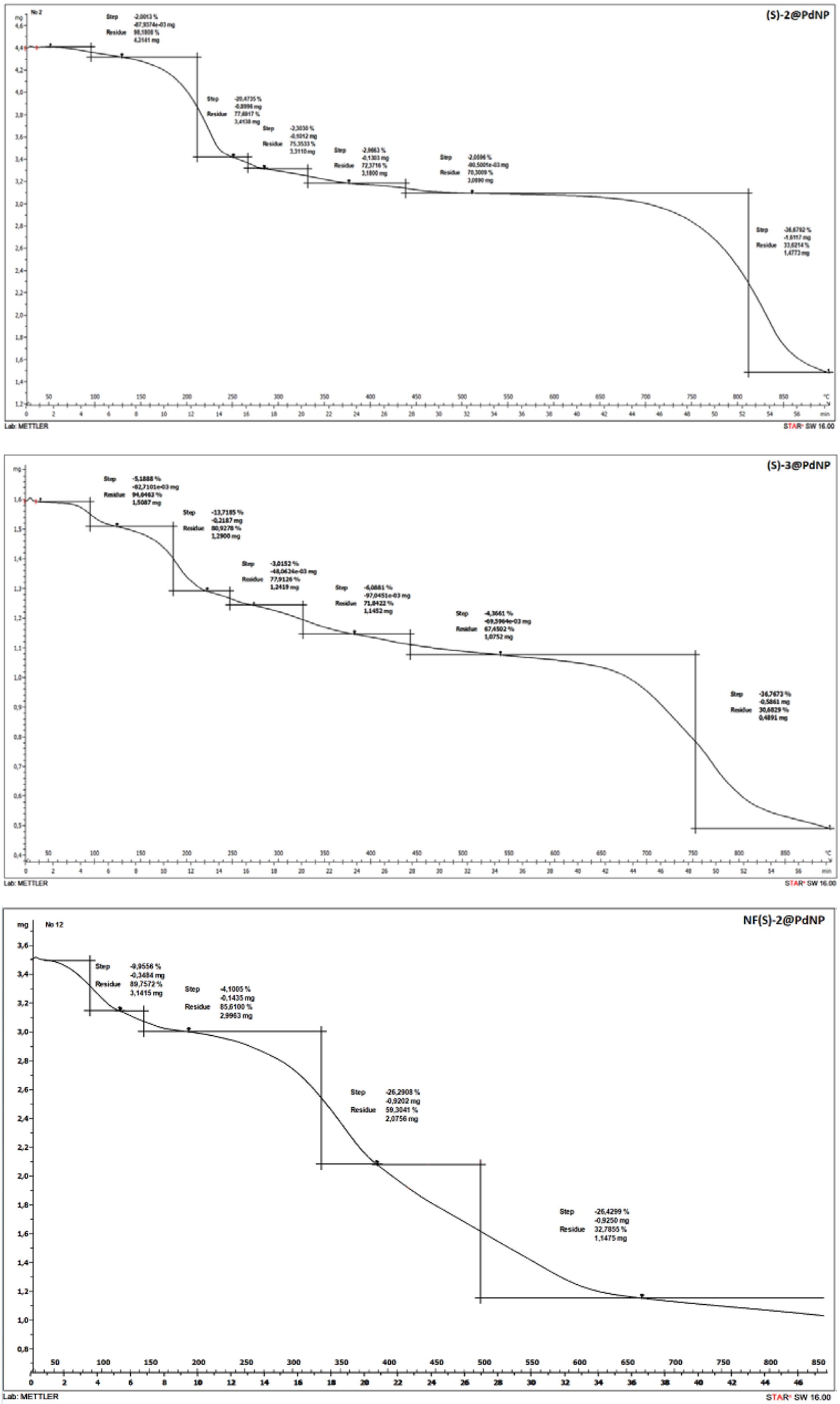

Figure S26: TGA spectra of (S)-1-3@PdNPs and NF-(S)-2@PdNP

6. TEM images of PdNPs after recycling

a) b) c)

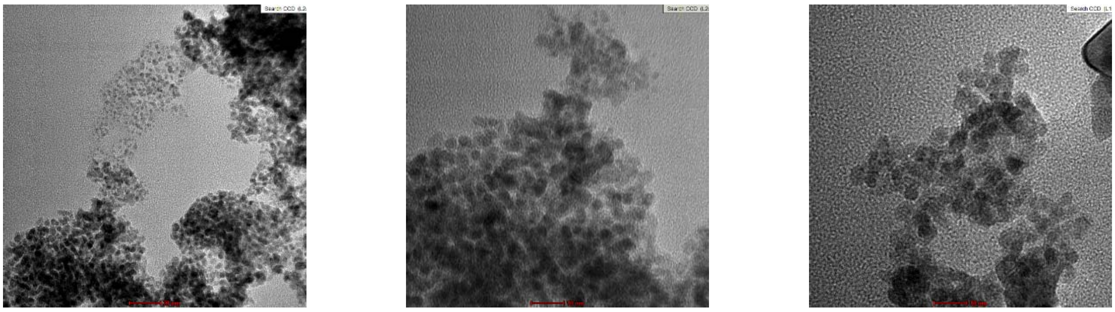

Figure S27: TEM images of (S)-2@PdNP after 2nd **(a)**, 4th **(b)** and 12th **(c)** catalytic cycles

7. HPLC chromatograms for the asymmetric Suzuki C-C coupling reactions

HPLC chromatogram of racemic 2-ethoxy-1,1'-binaphthyl (Chiralpak AD-H, n-hexane/i-PrOH:98/2, 0.25 mL/min, 254 nm)

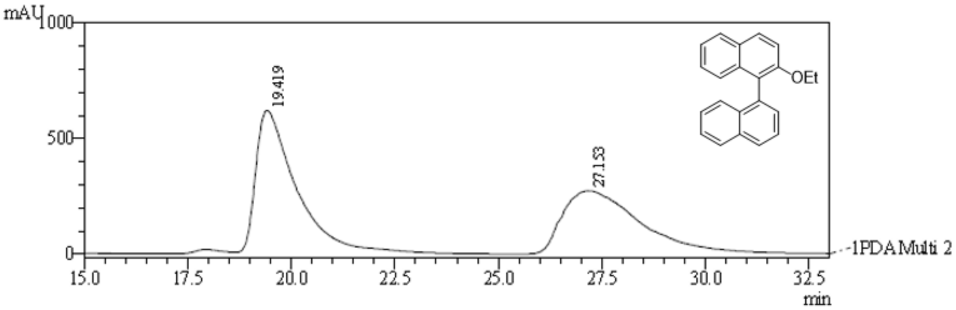

1 PDA Multi 2 / 254nm 4nm

PeakTable

PDA Ch2 254nm 4nm

Peak#	Name	Ret. Time	Area	Height	Area %
1	(S)-2-ethoxy-1,1'-binaphthalene	19.419	38442481	610793	49.647
2	(R)-2-ethoxy-1,1'-binaphthalene	27.153	38989608	278608	50.353
Total			77432089	889401	100.000

HPLC chromatogram of Table 2-Entry 3

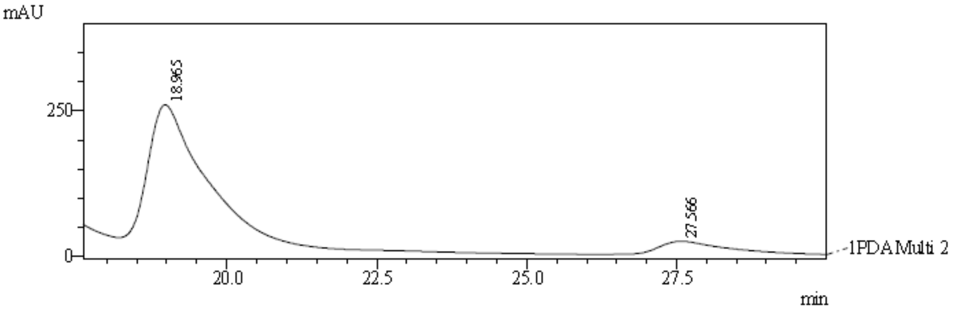

1 PDA Multi 2 / 254nm 4nm

PeakTable

PDA Ch2 254nm 4nm

Peak#	Ret. Time	Area	Height	Area %
1	18.965	14907494	231498	91.244
2	27.566	1430493	21046	8.756
Total		16337987	252544	100.000

HPLC chromatogram of Table 2-Entry 4

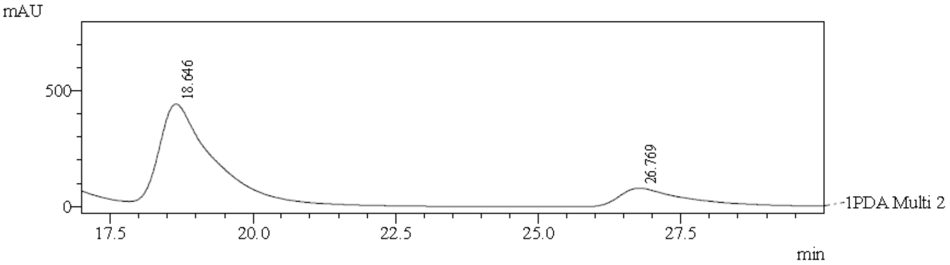

1 PDA Multi 2 / 254nm 4nm

PeakTable

PDA Ch2 254nm 4nm

Peak#	Name	Ret. Time	Area	Height	Area %
1	2-ethoxy-1,1'-binaphthalene	18.646	26406462	424036	83.761
2	2-ethoxy-1,1'-binaphthalene	26.769	5119666	72943	16.239
Total			31526128	496980	100.000

HPLC chromatogram of Table 2-Entry 8

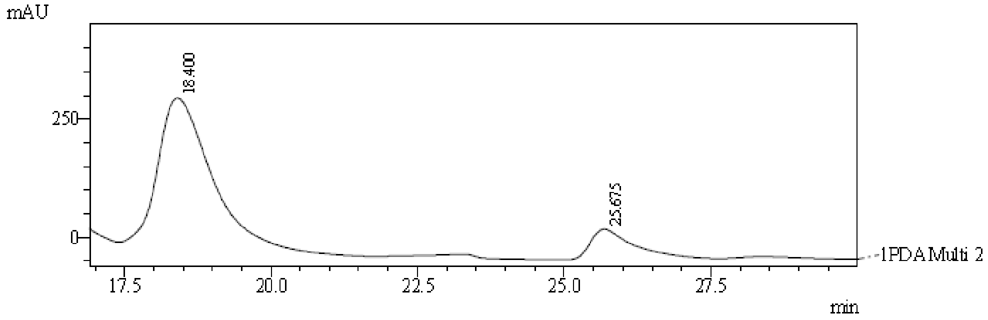

1 PDA Multi 2 / 254nm 4nm

PeakTable

PDA Ch2 254nm 4nm

Peak#	Ret. Time	Area	Height	Area %
1	18.400	17834227	303257	87.862
2	25.675	2463763	59230	12.138
Total		20297990	362487	100.000

HPLC chromatogram of Table 2-Entry 9

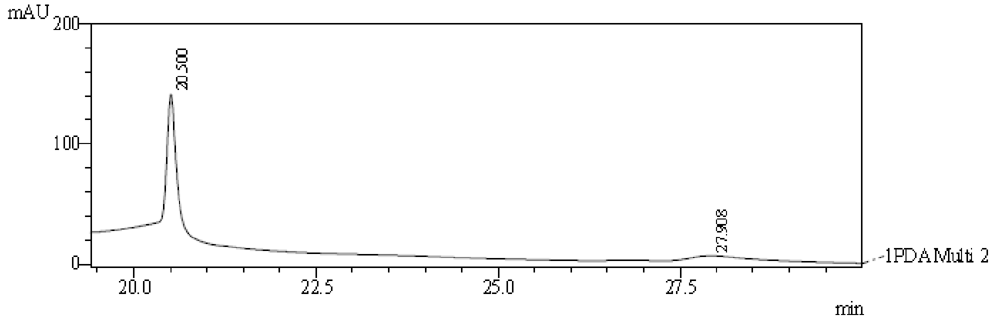

1 PDA Multi 2 / 254nm 4nm

PeakTable

PDA Ch2 254nm 4nm

Peak#	Ret. Time	Area	Height	Area %
1	20.500	1138894	116231	85.711
2	27.908	189867	4211	14.289
Total		1328761	120442	100.000

HPLC chromatogram of Table 2-Entry 11

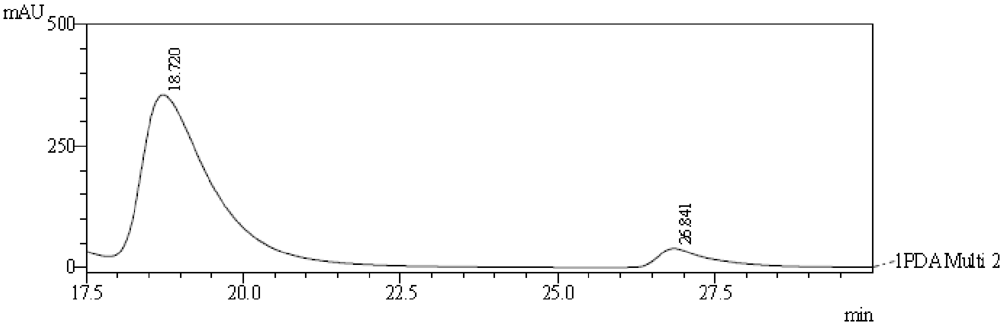

1 PDA Multi 2 / 254nm 4nm

PeakTable

PDA Ch2 254nm 4nm

Peak#	Ret. Time	Area	Height	Area %
1	18.720	23922480	332138	92.012
2	26.841	2076798	37865	7.988
Total		25999278	370002	100.000

HPLC chromatogram of Table 2-Entry 12

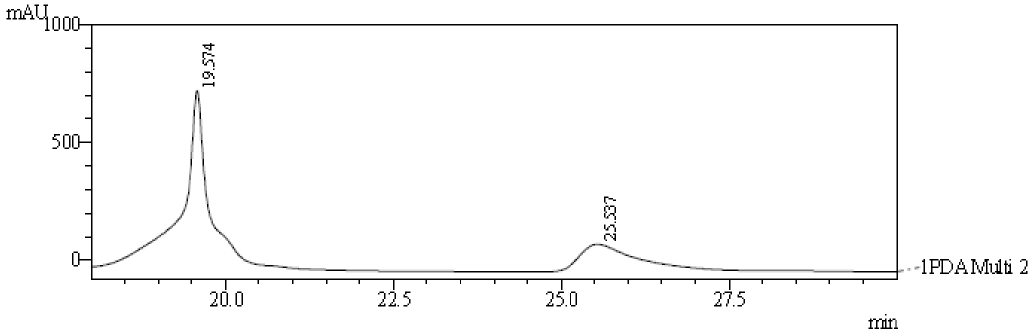

1 PDA Multi 2 / 254nm 4nm

PeakTable

PDA Ch2 254nm 4nm

Peak#	Ret. Time	Area	Height	Area %
1	19.574	21104266	746244	77.687
2	25.537	6061593	110447	22.313
Total		27165858	856691	100.000

HPLC chromatogram of Table 2-Entry 13

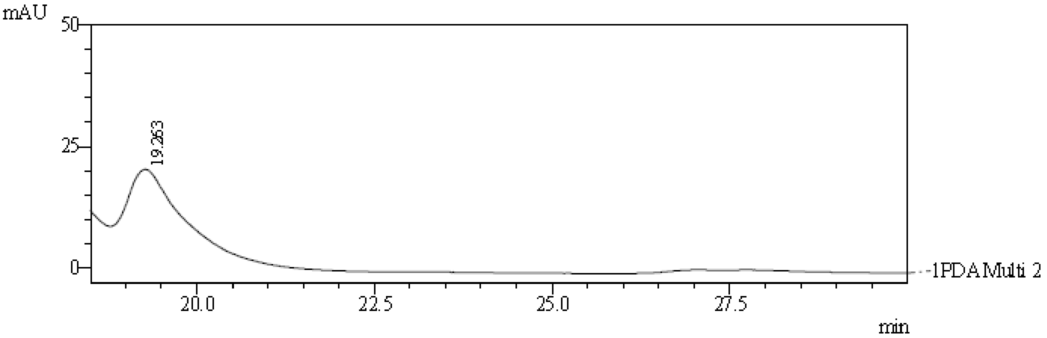

1 PDA Multi 2 / 254nm 4nm

PeakTable

PDA Ch2 254nm 4nm

Peak#	Ret. Time	Area	Height	Area %
1	19.263	584732	13339	100.000
Total		584732	13339	100.000

HPLC chromatogram of Table 2-Entry 14

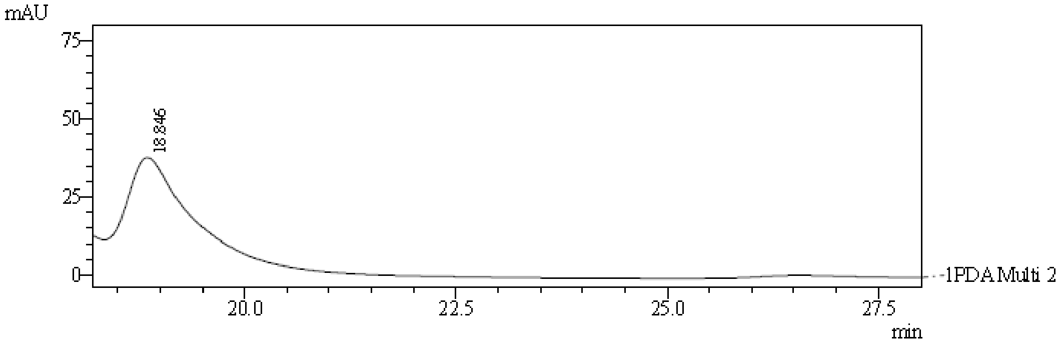

1 PDA Multi 2 / 254nm 4nm

PeakTable

PDA Ch2 254nm 4nm

Peak#	Ret. Time	Area	Height	Area %
1	18.846	3097895	43725	100.000
Total		3097895	43725	100.000

HPLC chromatogram of Table 2-Entry 15

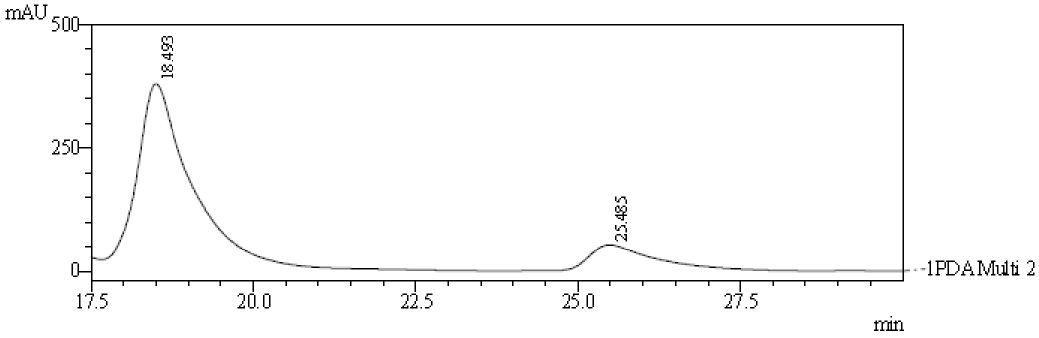

1 PDA Multi 2 / 254nm 4nm

PeakTable

PDA Ch2 254nm 4nm

Peak#	Ret. Time	Area	Height	Area %
1	18.493	18753574	355919	81.923
2	25.485	4138009	55360	18.077
Total		22891583	411278	100.000

HPLC chromatogram of Table 2-Entry 16

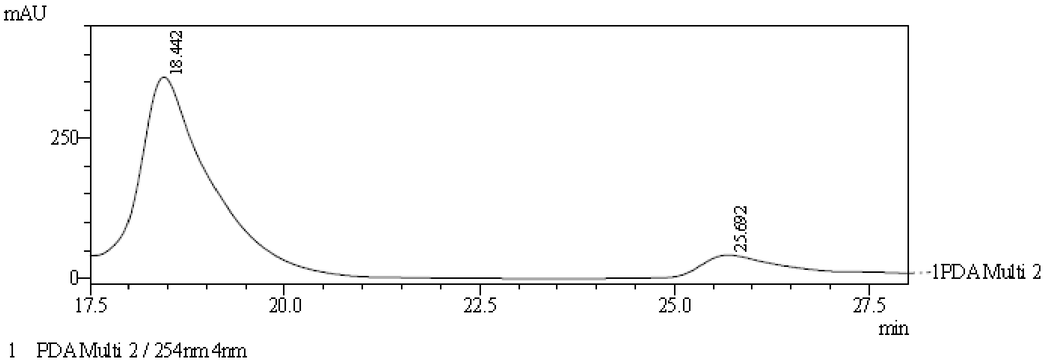

1 PDA Multi 2 / 254nm 4nm

PeakTable

PDA Ch2 254nm 4nm

Peak#	Ret. Time	Area	Height	Area %
1	18.442	19161488	327767	89.911
2	25.692	2150228	36058	10.089
Total		21311716	363825	100.000

HPLC chromatogram of Table 2-Entry 17

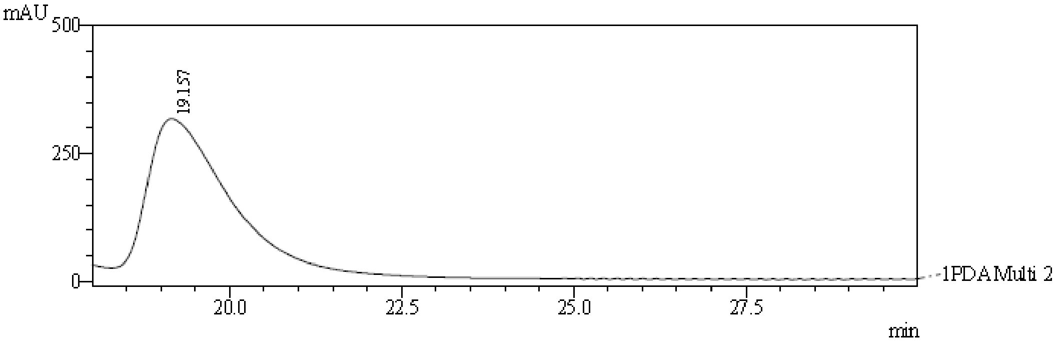

1 PDA Multi 2 / 254nm 4nm

PeakTable

PDA Ch2 254nm 4nm

Peak#	Ret. Time	Area	Height	Area %
1	19.157	22889240	292533	100.000
Total		22889240	292533	100.000

HPLC chromatogram of Table 2-Entry 19

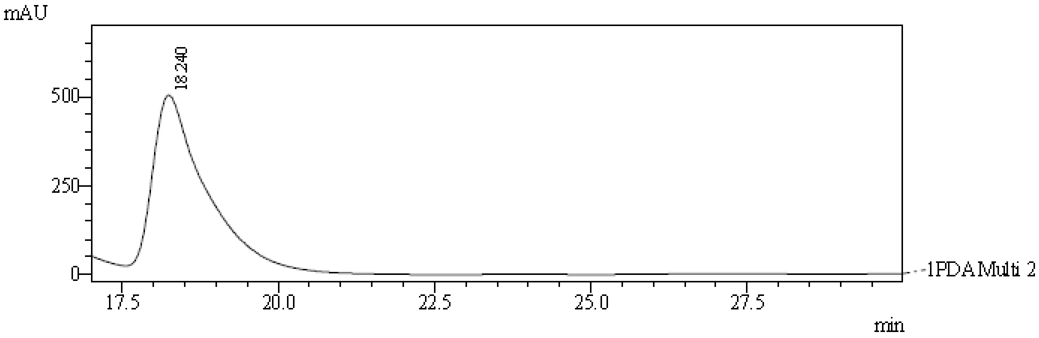

1 PDA Multi 2 / 254nm 4nm

PeakTable

PDA Ch2 254nm 4nm

Peak#	Ret. Time	Area	Height	Area %
1	18.240	27075760	480884	100.000
Total		27075760	480884	100.000

HPLC chromatogram of Table 2-Entry 20

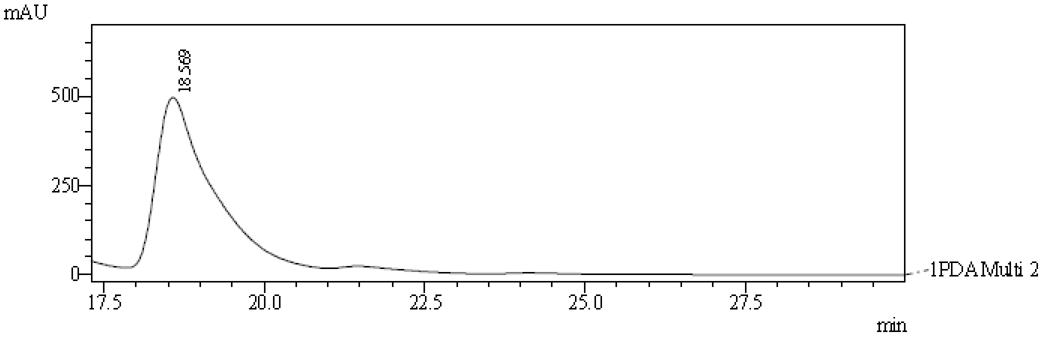

1 PDA Multi 2 / 254nm 4nm

PeakTable

PDA Ch2 254nm 4nm

Peak#	Ret. Time	Area	Height	Area %
1	18.569	28362033	477394	100.000
Total		28362033	477394	100.000

HPLC chromatogram of Table 2-Entry 21

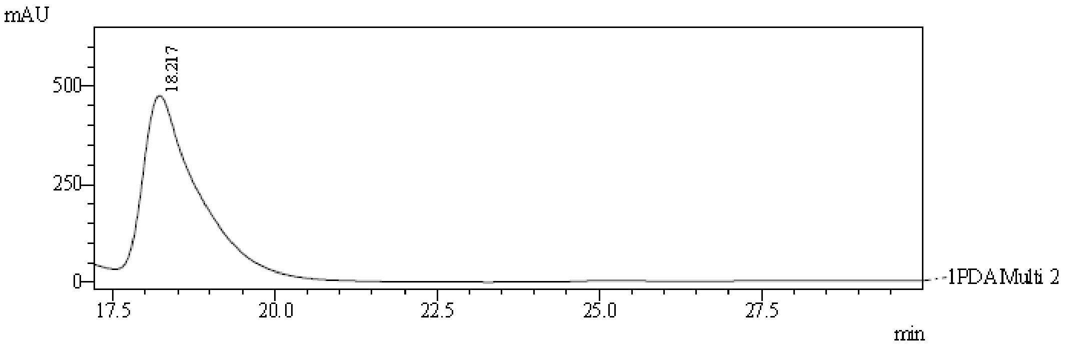

1 PDA Multi 2 / 254nm 4nm

PeakTable

PDA Ch2 254nm 4nm

Peak#	Ret. Time	Area	Height	Area %
1	18.217	24930172	446737	100.000
Total		24930172	446737	100.000

HPLC chromatogram of Table 2-Entry 22

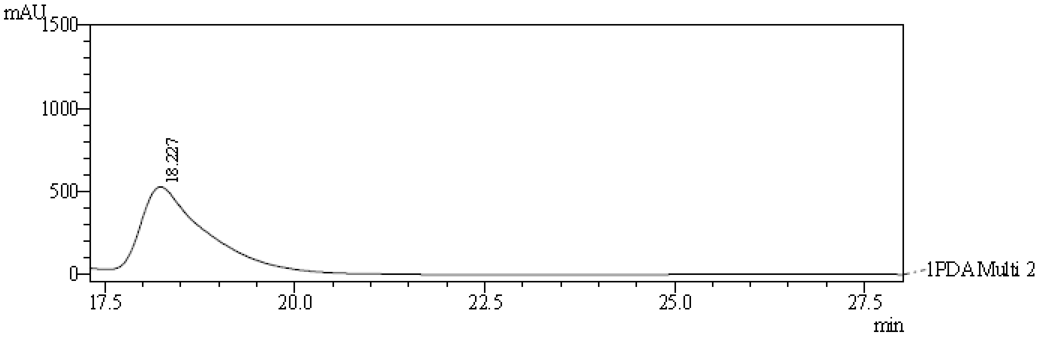

1 PDA Multi 2 / 254nm 4nm

PeakTable

PDA Ch2 254nm 4nm

Peak#	Ret. Time	Area	Height	Area %
1	18.227	28925907	500407	100.000
Total		28925907	500407	100.000

HPLC chromatogram of Table 2-Entry 23

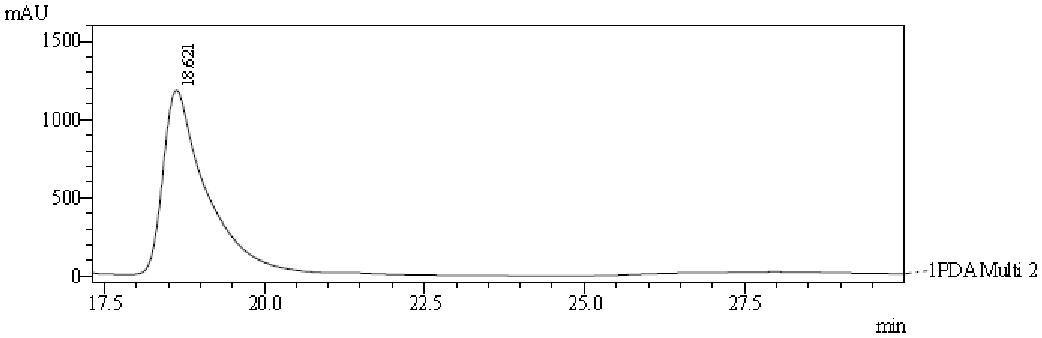

1 PDA Multi 2 / 254nm 4nm

PeakTable

PDA Ch2 254nm 4nm

Peak#	Ret. Time	Area	Height	Area %
1	18.621	54543304	1172049	100.000
Total		54543304	1172049	100.000

HPLC chromatogram of Table 2-Entry 24

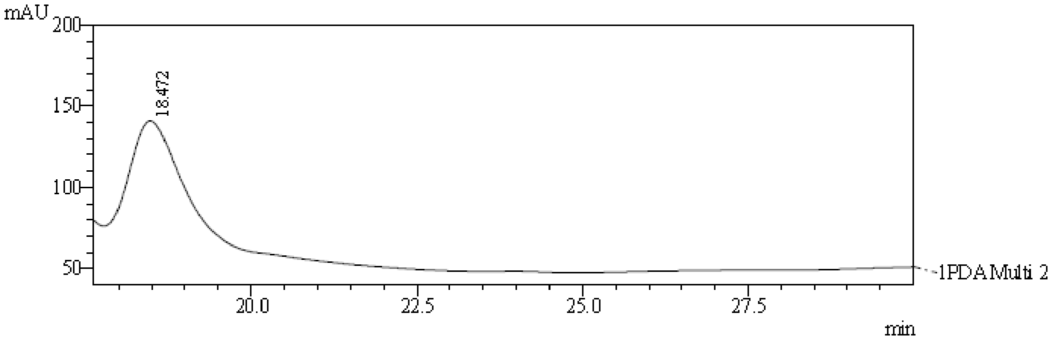

1 PDA Multi 2 / 254nm 4nm

PeakTable

PDA Ch2 254nm 4nm

Peak#	Ret. Time	Area	Height	Area %
1	18.472	2409280	58581	100.000
Total		2409280	58581	100.000

HPLC chromatogram of Table 2-Entry 25

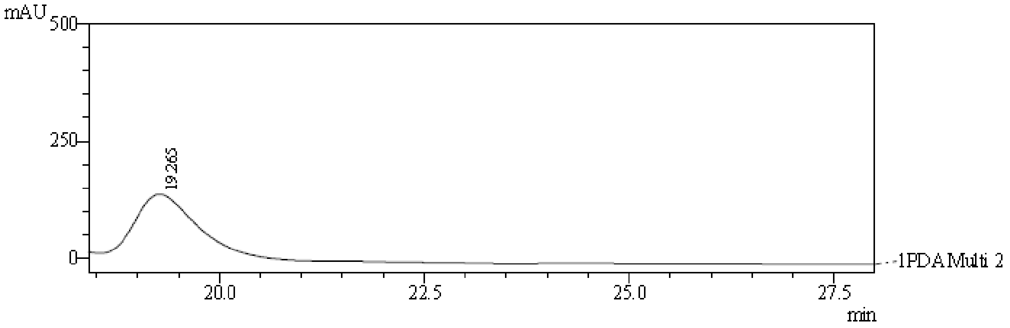

1 PDA Multi 2 / 254nm 4nm

PeakTable

PDA Ch2 254nm 4nm

Peak#	Ret. Time	Area	Height	Area %
1	19.265	6448689	129094	100.000
Total		6448689	129094	100.000

HPLC chromatogram of Table 2-Entry 26

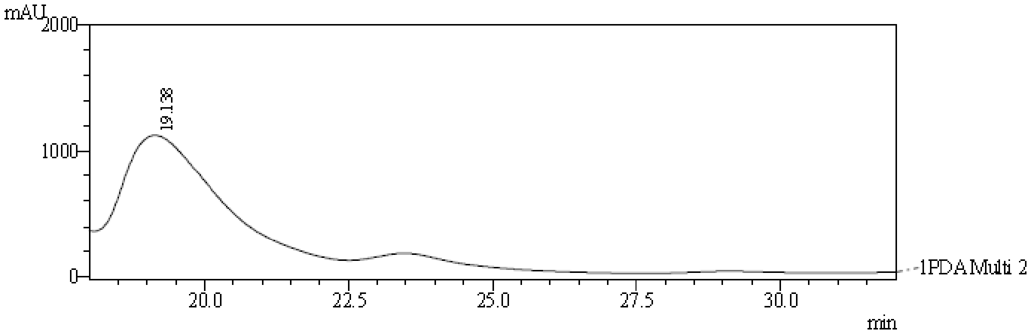

1 PDA Multi 2 / 224nm 4nm

PeakTable

PDA Ch2 224nm 4nm

Peak#	Ret. Time	Area	Height	Area %
1	19.138	92764047	899319	100.000
Total		92764047	899319	100.000

HPLC chromatogram of Table 2-Entry 27

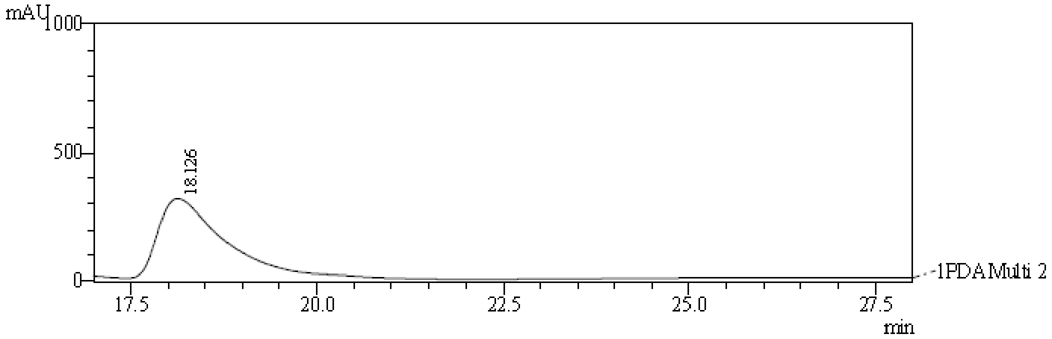

1 PDA Multi 2 / 254nm 4nm

PeakTable

PDA Ch2 254nm 4nm

Peak#	Ret. Time	Area	Height	Area %
1	18.126	19375927	308397	100.000
Total		19375927	308397	100.000

HPLC chromatogram of Table 2-Entry 27-2

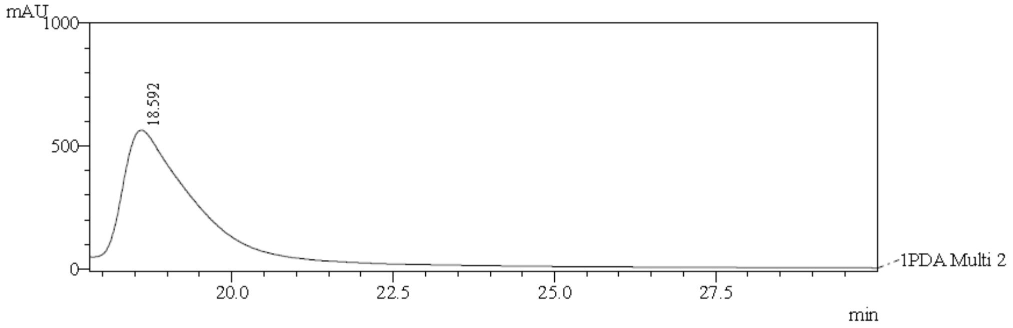

1 PDA Multi 2 / 254nm 4nm

PeakTable

PDA Ch2 254nm 4nm

Peak#	Ret. Time	Area	Height	Area %
1	18.592	33199686	508959	100.000
Total		33199686	508959	100.000

HPLC chromatogram of racemic 2-methoxy-1-(o-tolyl)naphthalene (Chiralpak OJ-H, n-hexane/i-PrOH:96/4, 0.3 mL/min, 224 nm)

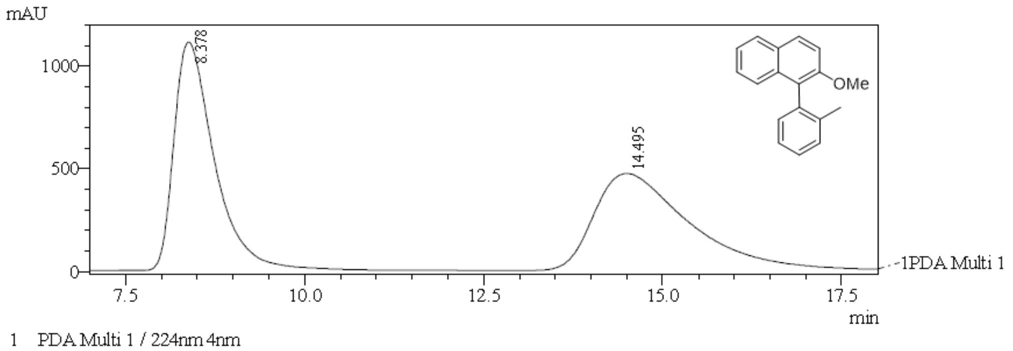

1 PDA Multi 1 / 224nm 4nm

PeakTable

PDA Ch1 224nm 4nm

Peak#	Name	Ret. Time	Area	Height	Area %
1	(S)-2-methoxy-1-(o-tolyl)naphthalene	8.378	43354086	1112617	50.460
2	(R)-2-methoxy-1-(o-tolyl)naphthalene	14.495	42563356	471208	49.540
Total			85917442	1583825	100.000

HPLC chromatogram of Table 3-Entry 1 (for (S)-1@PdNP)

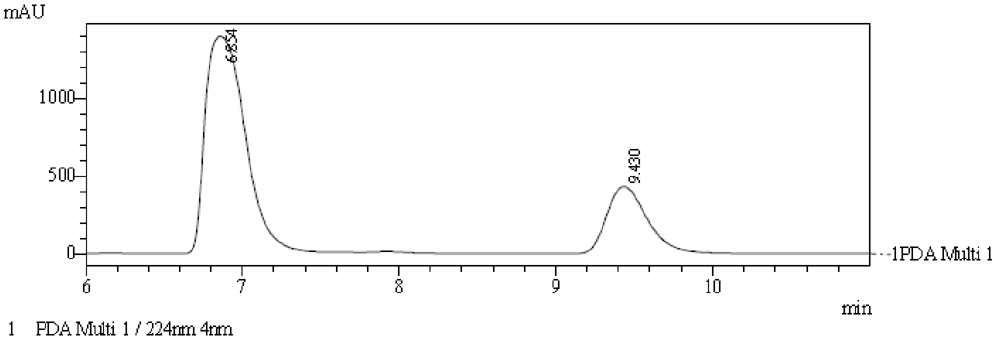

PeakTable

PDA Ch1 224nm 4nm

Peak#	Ret. Time	Area	Height	Area %
1	6.854	24598288	1395609	76.246
2	9.430	7663584	429628	23.754
Total		32261872	1825237	100.000

HPLC chromatogram of Table 3-Entry 1 (for (S)-2@PdNP)

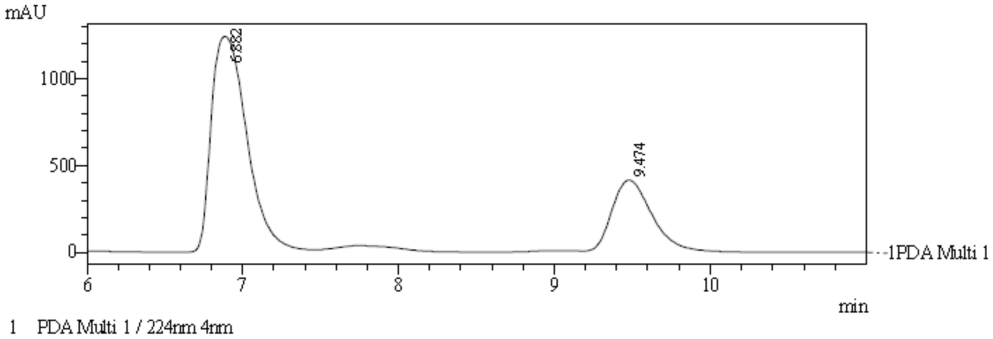

PeakTable

PDA Ch1 224nm 4nm

Peak#	Ret. Time	Area	Height	Area %
1	6.882	19465093	1238589	73.480
2	9.474	7025181	403889	26.520
Total		26490274	1642478	100.000

HPLC chromatogram of Table 3-Entry 1 (for (S)-3@PdNP)

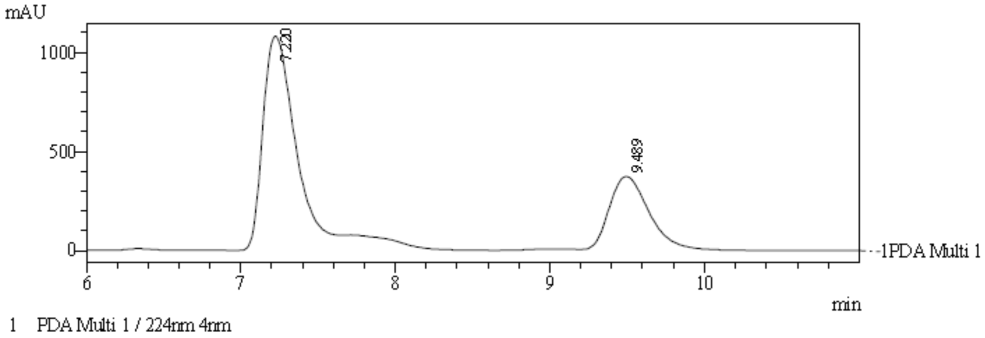

PeakTable

PDA Ch1 224nm 4nm

Peak#	Ret. Time	Area	Height	Area %
1	7.220	17637151	1082141	72.952
2	9.489	6539207	369086	27.048
Total		24176358	1451227	100.000

HPLC chromatogram of racemic 2-ethoxy-1-(o-tolyl)naphthalene (Chiralpak AD-H, n-hexane/i-PrOH:96/4, 0.5 mL/min, 224 nm)

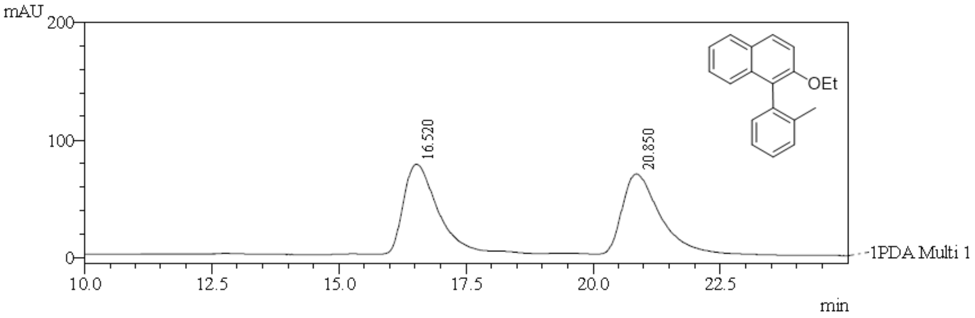

1 PDA Multi 1 / 254nm 4nm

PeakTable

PDA Ch1 254nm 4nm

Peak#	Name	Ret. Time	Area	Height	Area %
1	(S)-2-ethoxy-1-(o-tolyl)naphthalene	16.520	3528466	76303	49.891
2	(R)-2-ethoxy-1-(o-tolyl)naphthalene	20.850	3543832	68419	50.109
Total			7072297	144722	100.000

HPLC chromatogram of Table 3-Entry 2 (for (S)-1@PdNP)

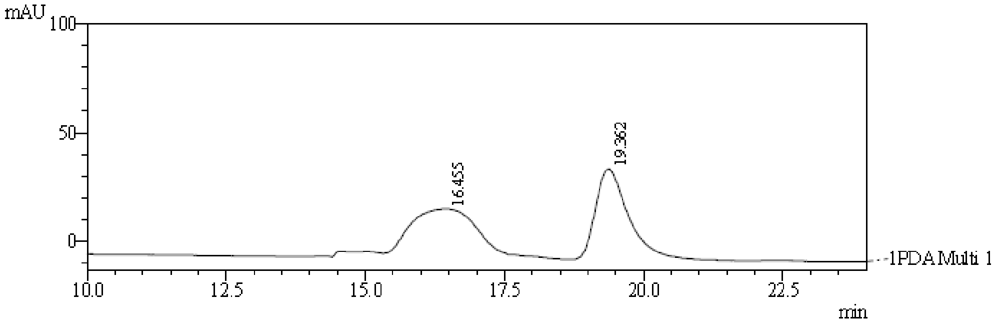

1 PDA Multi 1 / 254nm 4nm

PeakTable

PDA Ch1 254nm 4nm

Peak#	Ret. Time	Area	Height	Area %
1	16.455	2104623	23535	55.256
2	19.362	1704236	41507	44.744
Total		3808859	65043	100.000

HPLC chromatogram of Table 3-Entry 2 (for (S)-2@PdNP)

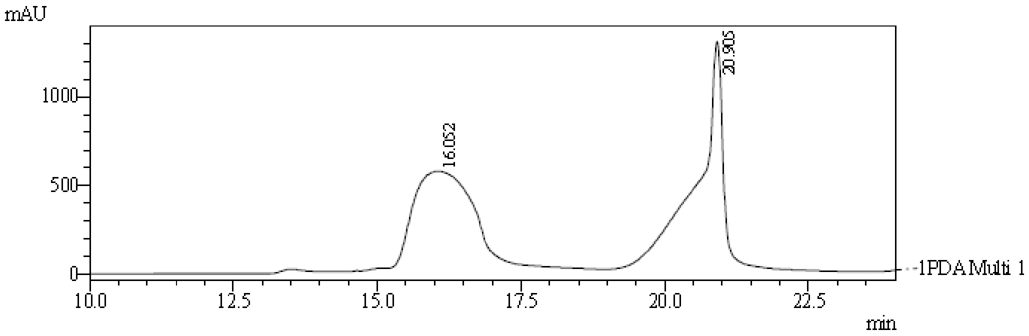

1 PDA Multi 1 / 254nm 4nm

PeakTable

PDA Ch1 254nm 4nm

Peak#	Ret. Time	Area	Height	Area %
1	16.052	42664219	562630	58.023
2	20.905	30865766	1186295	41.977
Total		73529985	1748924	100.000

HPLC chromatogram of Table 3-Entry 2 (for (S)-3@PdNP)

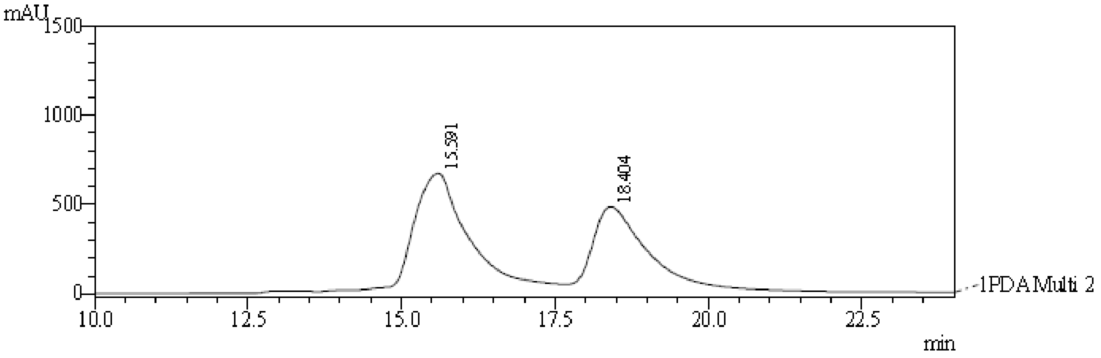

1 PDA Multi 2 / 254nm 4nm

PeakTable

PDA Ch2 254nm 4nm

Peak#	Ret. Time	Area	Height	Area %
1	15.591	35860610	634771	64.485
2	18.404	19749908	413539	35.515
Total		55610518	1048310	100.000

HPLC chromatogram of racemic 2-methoxy-2'-methyl-1,1'-binaphthalene (Chiralpak AD-H, n-hexane/i-PrOH:99/1, 1.0 mL/min, 254 nm)

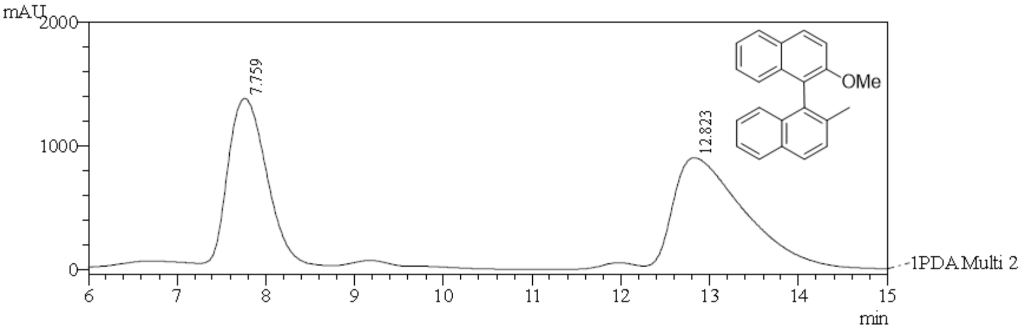

1 PDA Multi 2 / 254nm 4nm

PDA Ch2 254nm 4nm

Peak#	Name	Ret. Time	Area	Height	Area %
1	(S)-2-methoxy-2'-methyl-1,1'-binaphthalene	7.759	39587087	1335846	49.626
2	(R)-2-methoxy-2'-methyl-1,1'-binaphthalene	12.823	40184011	841004	50.374
Total			79771098	2176850	100.000

HPLC chromatogram of Table 3-Entry 3 (for (S)-1@PdNP)

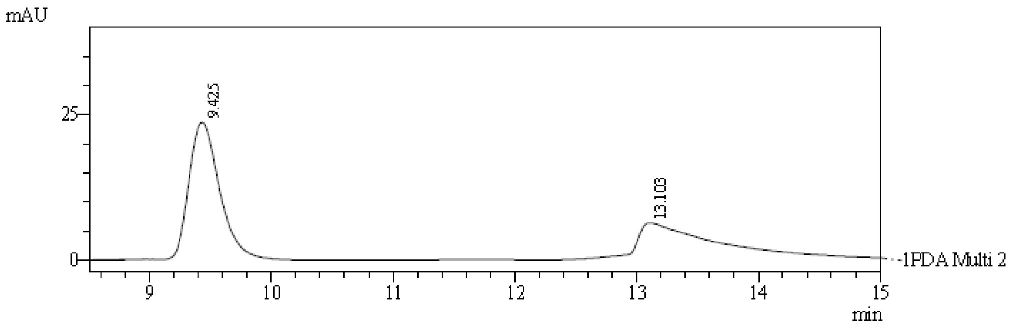

1 PDA Multi 2 / 254nm 4nm

PeakTable

PDA Ch2 254nm 4nm

Peak#	Ret. Time	Area	Height	Area %
1	9.425	409430	23558	83.472
2	13.103	81072	4151	16.528
Total		490502	27709	100.000

HPLC chromatogram of Table 3-Entry 3 (for (S)-2@PdNP)

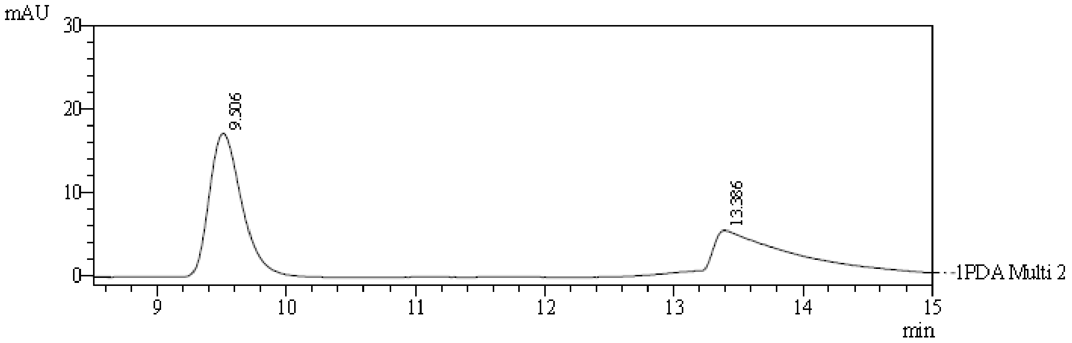

1 PDA Multi 2 / 254nm 4nm

PeakTable

PDA Ch2 254nm 4nm

Peak#	Ret. Time	Area	Height	Area %
1	9.506	303108	17213	79.999
2	13.386	75784	4092	20.001
Total		378892	21305	100.000

HPLC chromatogram of Table 3-Entry 3 (for (S)-3@PdNP)

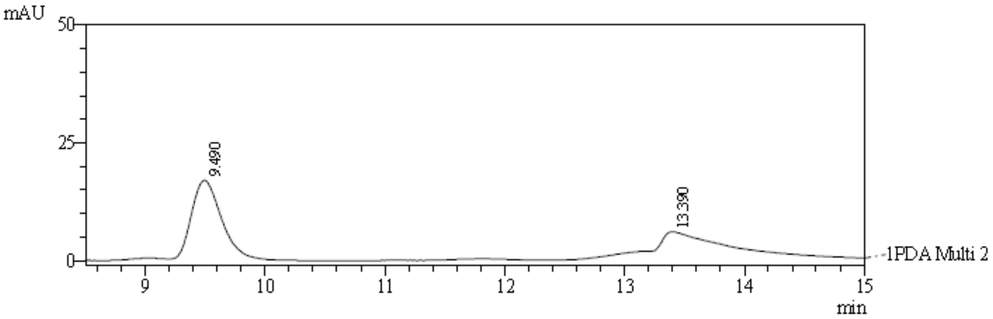

1 PDA Multi 2 / 254nm 4nm

PeakTable

PDA Ch2 254nm 4nm

Peak#	Ret. Time	Area	Height	Area %
1	9.490	287930	16714	81.663
2	13.390	64654	4081	18.337
Total		352584	20795	100.000

HPLC chromatogram of racemic 2-ethoxy-2'-methyl-1,1'-binaphthalene (Chiralpak AD-H, n-hexane/i-PrOH:95/5, 0.5 mL/min, 254 nm)

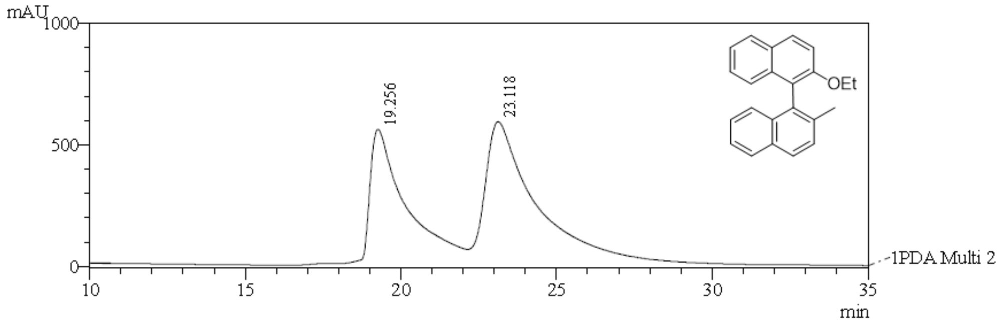

1 PDA Multi 2 / 254nm 4nm

PeakTable

PDA Ch2 254nm 4nm

Peak#	Name	Ret. Time	Area	Height	Area %
1	(S)-2-ethoxy-2'-methyl-1,1'-binaphthalene	19.256	44750894	552530	49.986
2	(R)-2-ethoxy-2'-methyl-1,1'-binaphthalene	23.118	44775882	519331	50.014
Total			89526775	1071861	100.000

HPLC chromatogram of Table 3-Entry 4 (for (S)-1@PdNP)

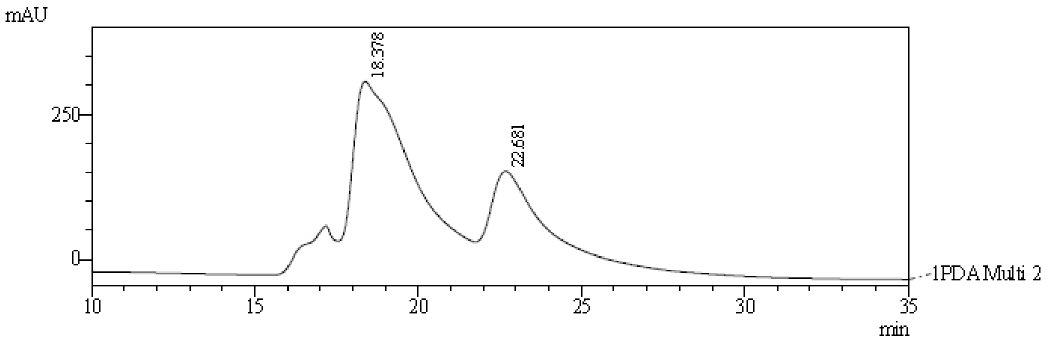

1 PDA Multi 2 / 254nm 4nm

PeakTable

PDA Ch2 254nm 4nm

Peak#	Ret. Time	Area	Height	Area %
1	18.378	29069192	275252	74.051
2	22.681	10186216	125857	25.949
Total		39255409	401108	100.000

HPLC chromatogram of Table 3-Entry 4 (for (S)-2@PdNP)

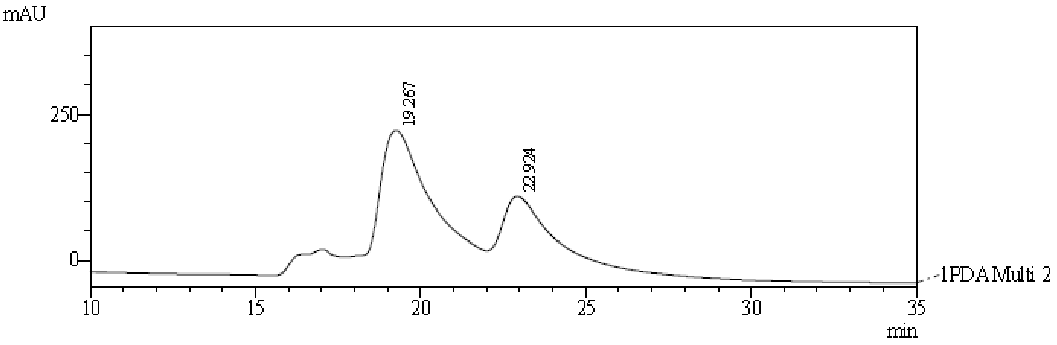

1 PDA Multi 2 / 254nm 4nm

PeakTable

PDA Ch2 254nm 4nm

Peak#	Ret. Time	Area	Height	Area %
1	19.267	23028826	221661	63.532
2	22.924	13218615	120943	36.468
Total		36247441	342604	100.000

HPLC chromatogram of Table 3-Entry 4 (for (S)-3@PdNP)

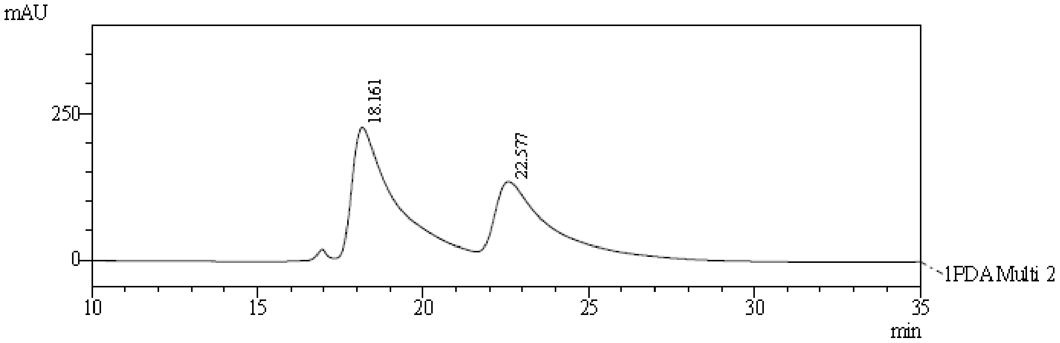

1 PDA Multi 2 / 254nm 4nm

PeakTable

PDA Ch2 254nm 4nm

Peak#	Ret. Time	Area	Height	Area %
1	18.161	16685978	213834	67.198
2	22.577	8145244	103813	32.802
Total		24831222	317648	100.000

HPLC chromatogram of Table 3-Entry 5 (for (S)-1@PdNP)

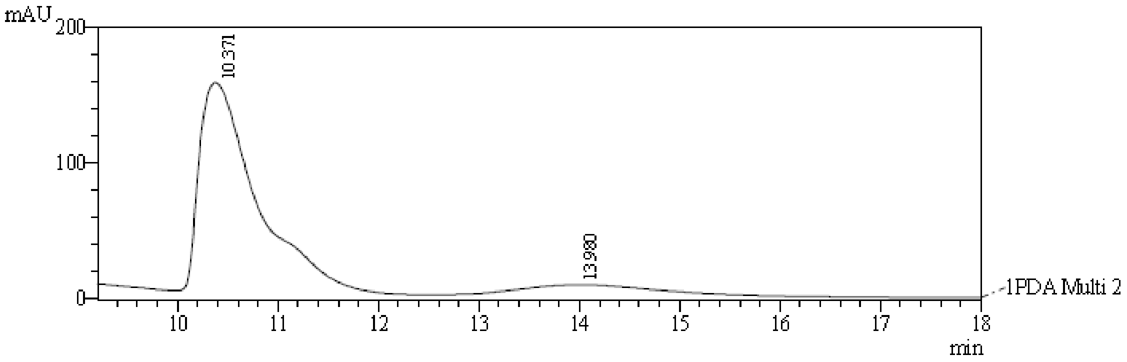

1 PDA Multi 2 / 254nm 4nm

PeakTable

PDA Ch2 254nm 4nm

Peak#	Ret. Time	Area	Height	Area %
1	10.371	6386536	156429	89.974
2	13.980	711678	8151	10.026
Total		7098214	164580	100.000

HPLC chromatogram of Table 3-Entry 5 (for (S)-2@PdNP)

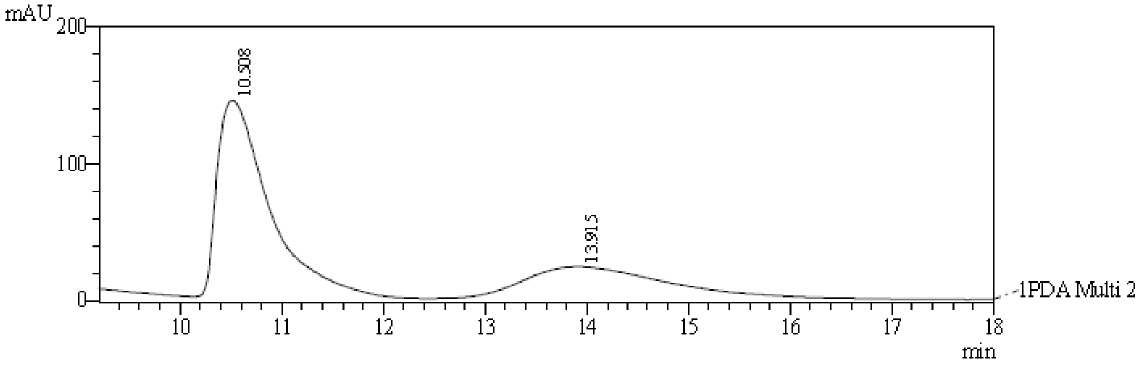

1 PDA Multi 2 / 254nm 4nm

PeakTable

PDA Ch2 254nm 4nm

Peak#	Ret. Time	Area	Height	Area %
1	10.508	4941564	140908	84.672
2	13.915	894555	14703	15.328
Total		5836119	155611	100.000

HPLC chromatogram of Table 3-Entry 5 (for (S)-3@PdNP)

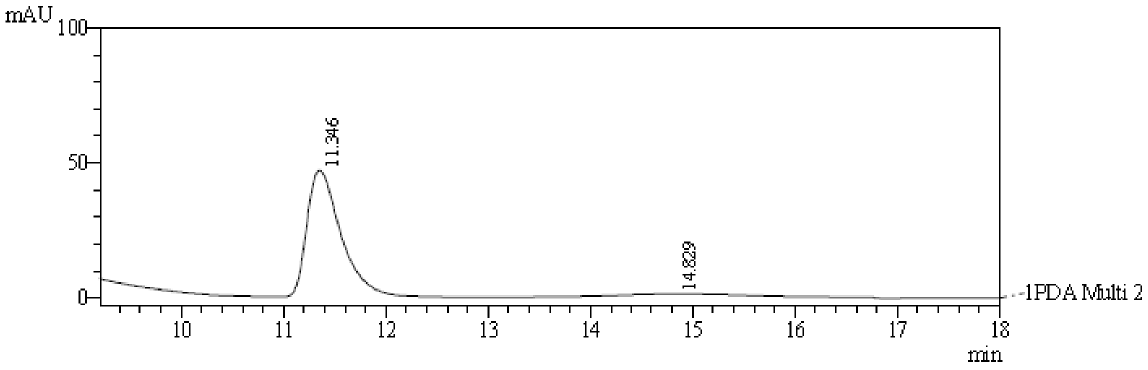

1 PDA Multi 2 / 254nm 4nm

PeakTable

PDA Ch2 254nm 4nm

Peak#	Ret. Time	Area	Height	Area %
1	11.346	1072379	46708	93.042
2	14.829	80200	1129	6.958
Total		1152578	47837	100.000

HPLC chromatogram of racemic 2-methyl-1,1'-binaphthalene (Chiralpak OJ-H, n-hexane/i-PrOH:95/5, 1.0 mL/min, 254 nm)

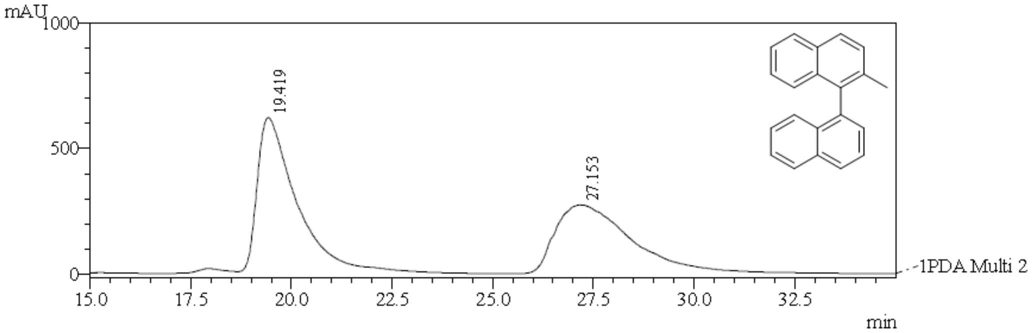

1 PDA Multi 2 / 254nm 4nm

PeakTable

PDA Ch2 254nm 4nm

Peak#	Name	Ret. Time	Area	Height	Area %
1	(S)-2-methyl-1,1'-binaphthalene	19.419	36537854	604487	50.147
2	(R)-2-methyl-1,1'-binaphthalene	27.153	36323764	275365	49.853
Total			72861618	879852	100.000

HPLC chromatogram of Table 3-Entry 6 (for (S)-1@PdNP)

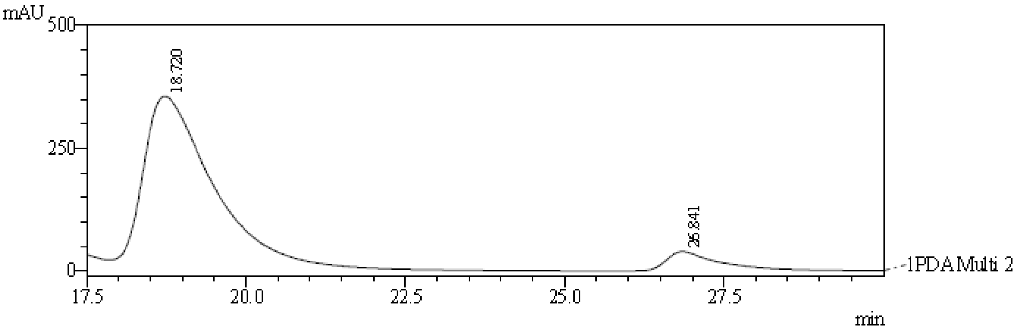

1 PDA Multi 2 / 254nm 4nm

PeakTable

PDA Ch2 254nm 4nm

Peak#	Ret. Time	Area	Height	Area %
1	18.720	23922480	332138	92.012
2	26.841	2076798	37865	7.988
Total		25999278	370002	100.000

HPLC chromatogram of Table 3-Entry 6 (for (S)-2@PdNP)

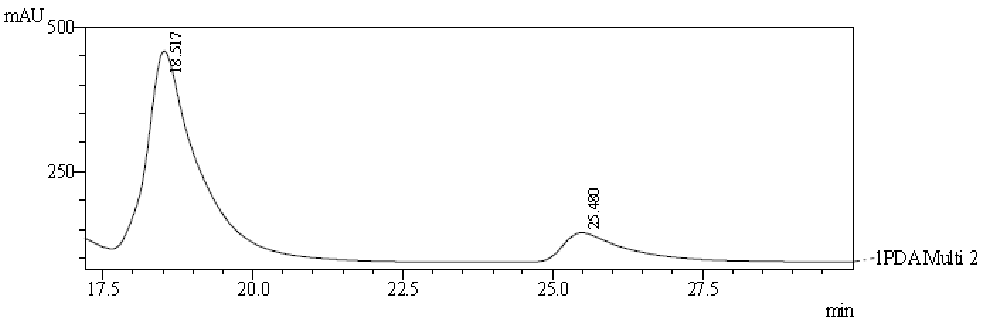

1 PDA Multi 2 / 254nm 4nm

PeakTable

PDA Ch2 254nm 4nm

Peak#	Ret. Time	Area	Height	Area %
1	18.517	16691394	334782	79.808
2	25.480	4223035	50846	20.192
Total		20914429	385628	100.000

HPLC chromatogram of Table 3-Entry 6 (for (S)-3@PdNP)

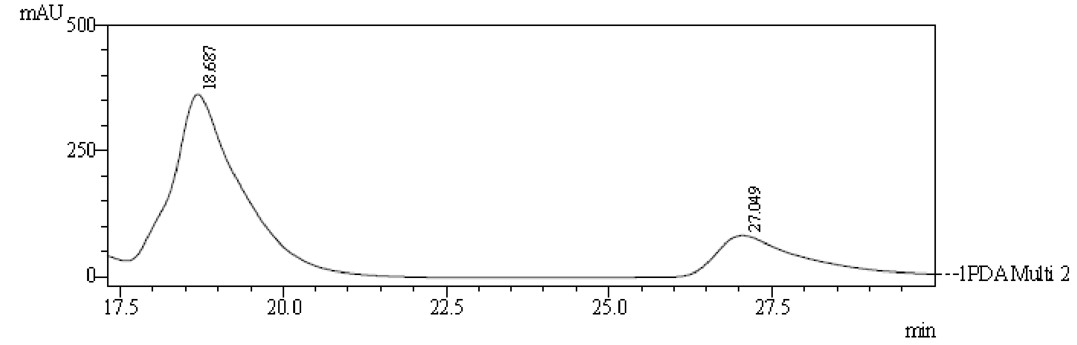

1 PDA Multi 2 / 254nm 4nm

PeakTable

PDA Ch2 254nm 4nm

Peak#	Ret. Time	Area	Height	Area %
1	18.687	23307186	339045	77.151
2	27.049	6902666	78897	22.849
Total		30209852	417941	100.000

HPLC chromatogram of Table 3-Entry 7 (for (S)-1@PdNP)

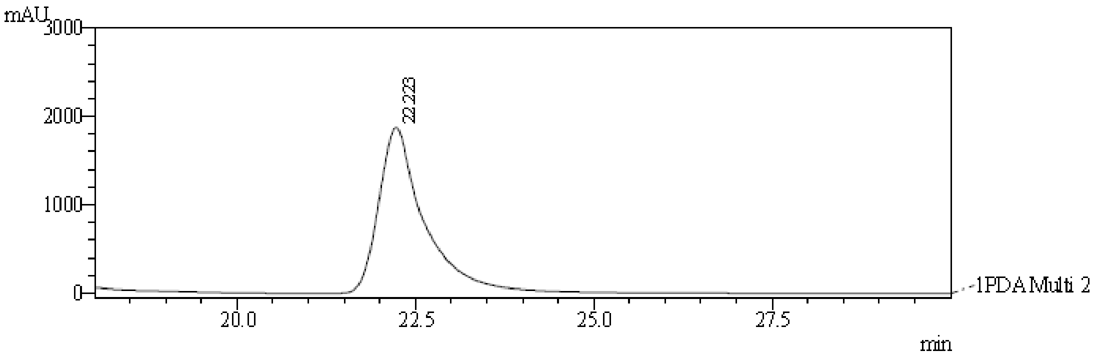

1 PDA Multi 2 / 254nm 4nm

PeakTable

PDA Ch2 254nm 4nm

Peak#	Ret. Time	Area	Height	Area %
1	22.223	81879964	1867269	100.000
Total		81879964	1867269	100.000

HPLC chromatogram of Table 3-Entry 7 (for (S)-2@PdNP)

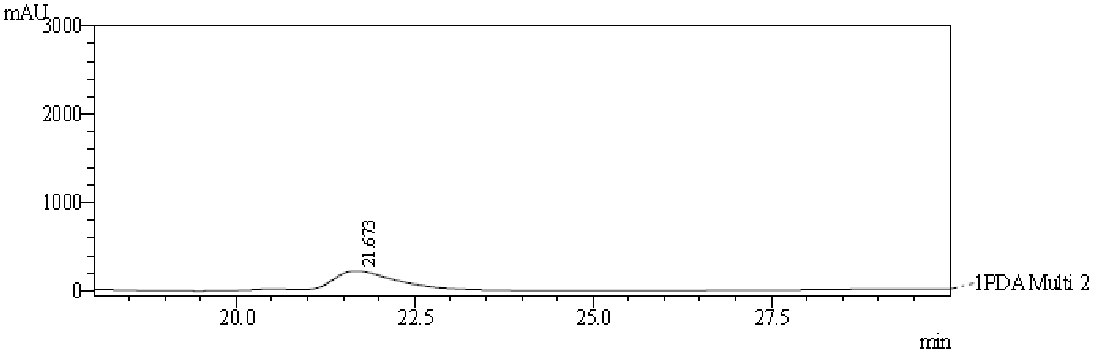

1 PDA Multi 2 / 254nm 4nm

PeakTable

PDA Ch2 254nm 4nm

Peak#	Ret. Time	Area	Height	Area %
1	21.673	12440096	212192	100.000
Total		12440096	212192	100.000

HPLC chromatogram of Table 3-Entry 7 (for (S)-3@PdNP)

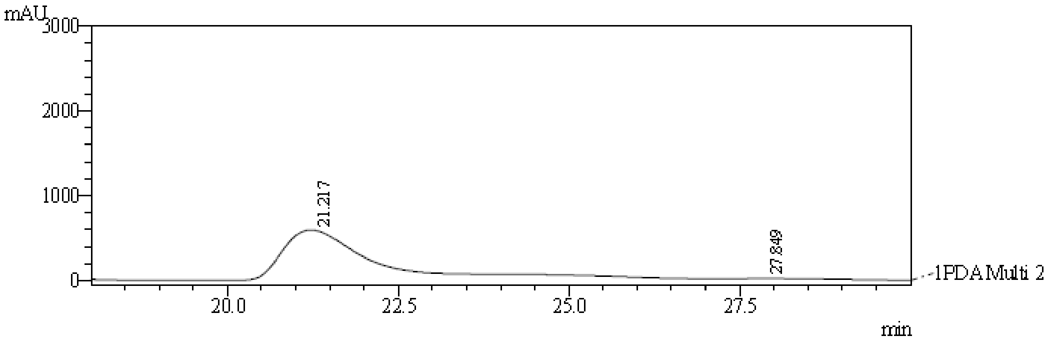

1 PDA Multi 2 / 254nm 4nm

PeakTable

PDA Ch2 254nm 4nm

Peak#	Ret. Time	Area	Height	Area %
1	21.217	38715147	556172	98.322
2	27.849	660565	9430	1.678
Total		39375711	565602	100.000

HPLC chromatogram of racemic 2-ethoxy-1,1'-binaphthalene (Chiralpak AD-H, n-hexane/i-PrOH:98/2, 0.25 mL/min, 254 nm)

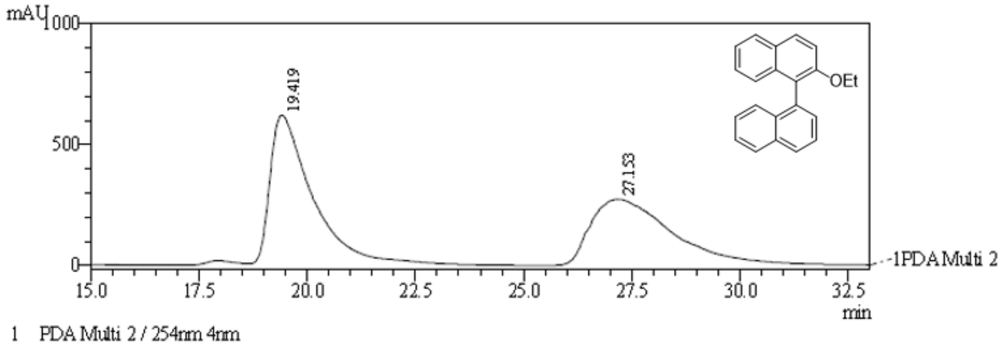

1 PDA Multi 2 / 254nm 4nm

PeakTable

PDA Ch2 254nm 4nm

Peak#	Name	Ret. Time	Area	Height	Area %
1	(S)-2-ethoxy-1,1'-binaphthalene	19.419	38442481	610793	49.647
2	(R)-2-ethoxy-1,1'-binaphthalene	27.153	38989608	278608	50.353
Total			77432089	889401	100.000

HPLC chromatogram of Table 3-Entry 8 (for (S)-1@PdNP)

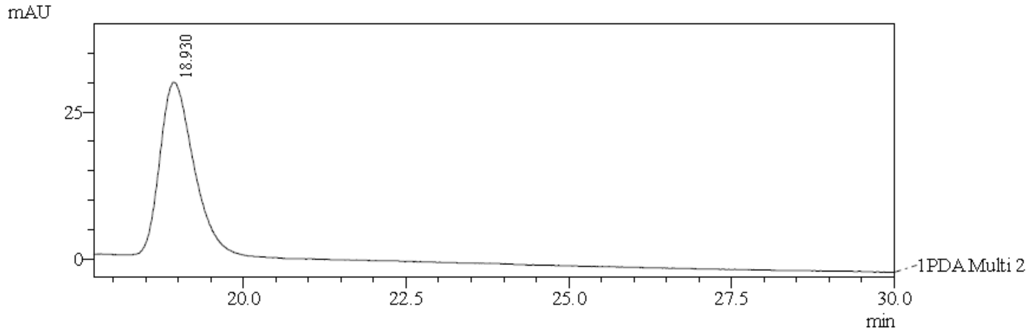

1 PDA Multi 2 / 254nm 4nm

PeakTable

PDA Ch2 254nm 4nm

Peak#	Ret. Time	Area	Height	Area %
1	18.930	1077502	29494	100.000
Total		1077502	29494	100.000

HPLC chromatogram of Table 3-Entry 8 (for (S)-2@PdNP)

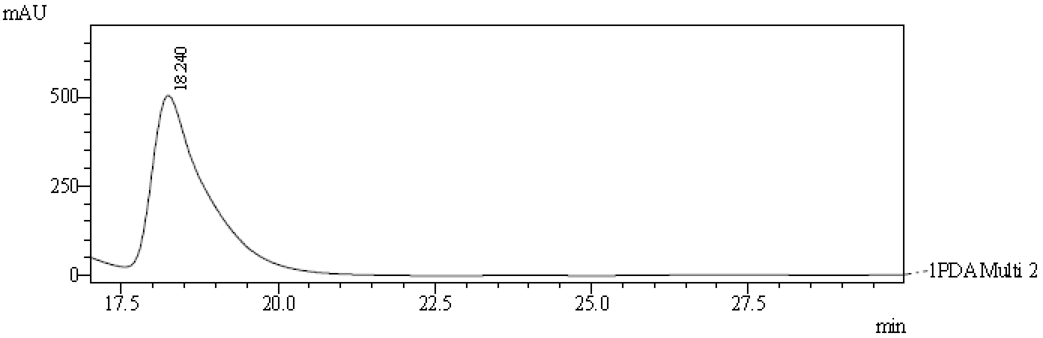

1 PDA Multi 2 / 254nm 4nm

PeakTable

PDA Ch2 254nm 4nm

Peak#	Ret. Time	Area	Height	Area %
1	18.240	27075760	480884	100.000
Total		27075760	480884	100.000

HPLC chromatogram of Table 3-Entry 8 (for (S)-3@PdNP)

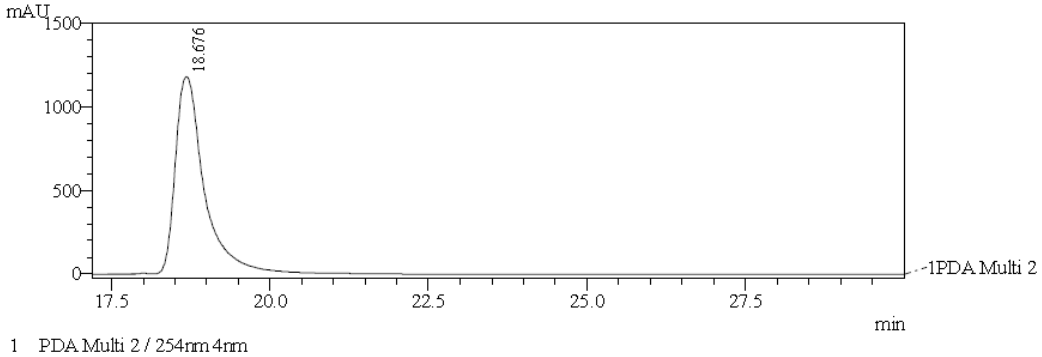

1 PDA Multi 2 / 254nm 4nm

PeakTable

PDA Ch2 254nm 4nm

Peak#	Ret. Time	Area	Height	Area %
1	18.676	36224867	1169253	100.000
Total		36224867	1169253	100.000

HPLC chromatogram of racemic 2-methoxy-1,1'-binaphthalene (Chiralpak OJ-H, n-hexane/i-PrOH:95/5, 1.0 mL/min, 224 nm)

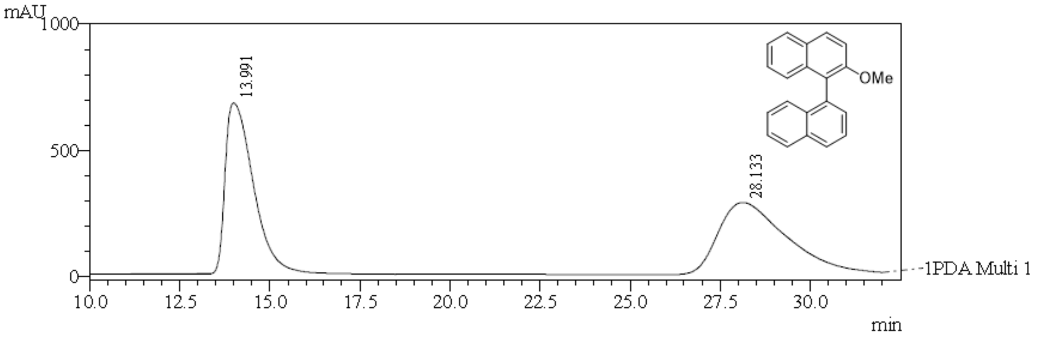

1 PDA Multi 1 / 224nm 4nm

PeakTable

PDA Ch1 224nm 4nm

Peak#	Name	Ret. Time	Area	Height	Area %
1	(S)-2-methoxy-1,1'-binaphthalene	13.991	36099274	674635	49.878
2	(R)-2-methoxy-1,1'-binaphthalene	28.133	36276196	281609	50.122
Total			72375469	956243	100.000

HPLC chromatogram of Table 3-Entry 9 (for (S)-1@PdNP)

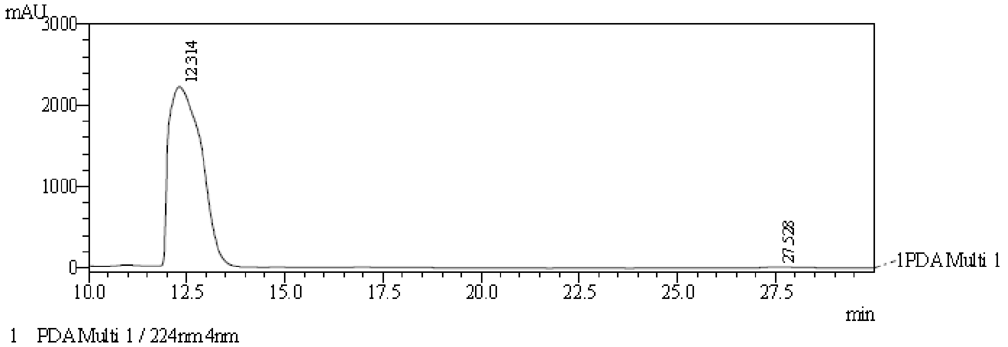

1 PDA Multi 1 / 224nm 4nm

PeakTable

PDA Ch1 224nm 4nm

Peak#	Ret. Time	Area	Height	Area %
1	12.314	127120488	2207179	99.451
2	27.528	702185	13619	0.549
Total		127822673	2220798	100.000

HPLC chromatogram of Table 3-Entry 9 (for (S)-2@PdNP)

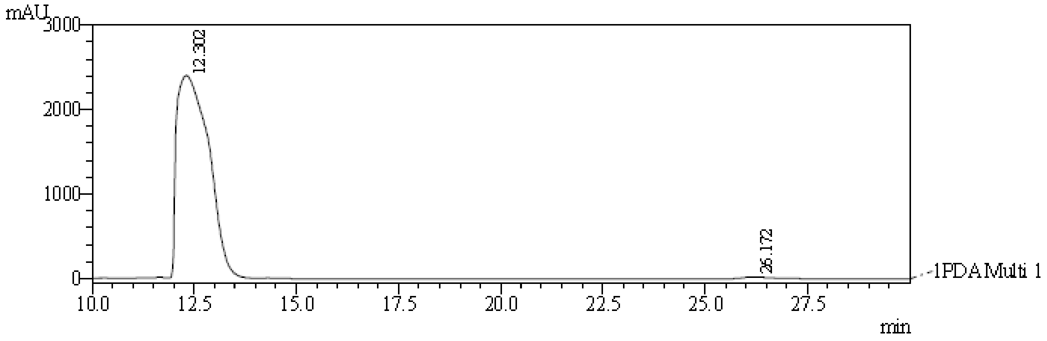

1 PDA Multi 1 / 224nm 4nm

PeakTable

PDA Ch1 224nm 4nm

Peak#	Ret. Time	Area	Height	Area %
1	12.302	130900202	2393574	99.366
2	26.172	835151	19777	0.634
Total		131735353	2413351	100.000

HPLC chromatogram of Table 3-Entry 9 (for (S)-3@PdNP)

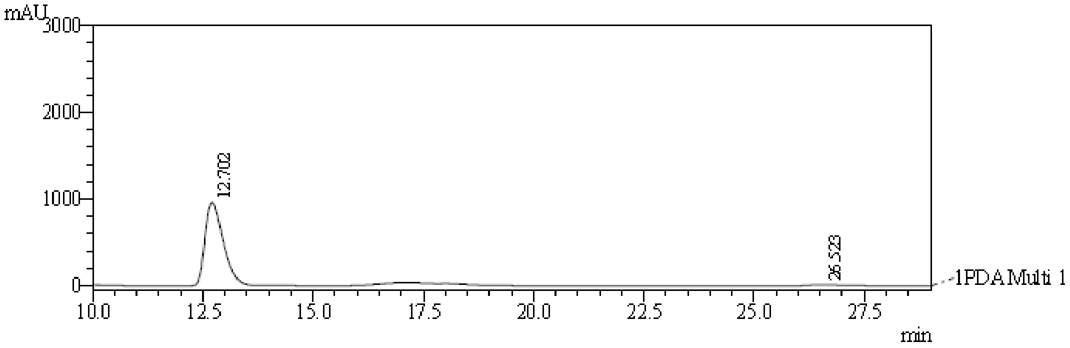

1 PDA Multi 1 / 224nm 4nm

PeakTable

PDA Ch1 224nm 4nm

Peak#	Ret. Time	Area	Height	Area %
1	12.702	27726323	956901	97.927
2	26.523	586839	13411	2.073
Total		28313162	970312	100.000
